# A longitudinal multilevel analysis of individual‐ and contextual‐level predictors of cross‐ethnic friendships in the UK

**DOI:** 10.1111/bjso.70068

**Published:** 2026-03-17

**Authors:** Rose Meleady, Hannah K. Peetz, Shelley McKeown, George Leckie, Jo Broadwood

**Affiliations:** ^1^ School of Psychology University of East Anglia Norwich UK; ^2^ Department of Experimental Psychology University of Oxford Oxford UK; ^3^ School of Education University of Bristol Bristol UK; ^4^ Belong – The Cohesion and Integration Network Manchester UK

**Keywords:** cross‐group friendships, intergroup contact, multilevel analysis, predictors of contact, understanding society

## Abstract

Intergroup contact plays a central role in fostering positive intergroup attitudes; yet, factors promoting intergroup contact are less understood. Using three waves of data from a nationally representative UK household panel study (*N* = 18,807), we applied longitudinal multilevel models to examine how individual‐ and objective neighbourhood‐level indicators jointly predict cross‐ethnic friendships. At the individual level, higher openness and agreeableness, stronger neighbourhood belonging and a left‐leaning political orientation were associated with more cross‐ethnic friendships. At the contextual level, intergroup friendships were more common in neighbourhoods with more structural opportunity for contact (i.e., areas with a lower proportion of same‐ethnic residents), and in areas with lower anti‐immigration norms (as indicated by local Brexit ‘Leave’ vote share). Crucially, cross‐level interactions highlighted the interplay of person and place: neighbourhood diversity fostered more cross‐ethnic friendships, especially among those with strong neighbourhood belonging, suggesting that people who feel embedded in their community are more likely to translate diverse surroundings into meaningful intergroup ties. Differences between the ethnic majority and minority groups also emerged. For example, higher objective area‐level racial hate crime incidence predicted more intergroup friendships among majority members, suggesting a possible repair response, but showed no association for minority members. Findings underscore the multilevel and group‐specific pathways to sustained intergroup friendships.

## BACKGROUND

The United Kingdom has undergone substantial demographic change in recent decades, with the proportion of residents born abroad having increased markedly since the mid‐twentieth century (ONS, 2022a) and ethnic minority groups now comprising nearly one in five people in England and Wales (ONS, 2022b). Public debates over immigration, integration, and national identity have become more intense and divided in recent years. Such issues took on heightened visibility around the Brexit referendum and have continued to dominate public and political discourse, including high‐profile discussions of migrant arrivals via small boats. Signs of growing ethnic divisions are evident: in 2025, 40% of the UK population believed that the declining share of White people in the population is harmful to society (NatCen, 2025), while racially motivated hate crimes have also increased, reaching 98,799 recorded incidents in 2023/2024 (Home Office, 2024).

Extensive psychological research has shown that positive intergroup contact – especially friendships across ethnic lines – can strengthen social cohesion and reduce prejudiced attitudes (Allport, 1954; Davies et al., 2011; Pettigrew, 1997; Pettigrew & Tropp, 2006). Yet, despite these benefits, contact between different ethnic groups remains less common than might be expected. Major governmental inquiries have highlighted persistent patterns of neighbourhood ethnic clustering, with many communities described as living ‘parallel lives’ due to limited cross‐ethnic interaction (Cantle, 2001; Casey, 2016), and nearly half of British adults report that none of the people they spend time with socially are from a different ethnic background (The Challenge, 2019).

Research exploring factors that facilitate or hinder intergroup contact engagement remains limited and fragmented. Much of the existing work focuses on a narrow set of predictors, typically at a single level of analysis, and relies heavily on cross‐sectional data (Kauff et al., 2020; Paolini et al., 2018; Ron et al., 2017). In contrast, recent scholarship emphasizes that intergroup attitudes and behaviour emerge from dynamic interactions between individuals and their social environments (Bou Zeineddine & Leach, 2021; Esses, 2021; Green & Staerklé, 2023; Miller & Laurin, 2025; Pettigrew, 2018). Addressing this gap, the present research draws on a large, nationally representative longitudinal dataset from the UK to better capture the real‐world complexity of intergroup contact by examining how individual‐ and neighbourhood‐level contextual factors jointly shape interethnic friendships.

### Predictors of intergroup contact: individual and contextual drivers

Initial explorations of the antecedents of intergroup contact have identified several individual‐level psychological factors that predict greater intergroup contact engagement. Certain personality traits, such as openness (Stürmer et al., 2013), extraversion (Stürmer et al., 2013; Turner et al., 2014; Vezzali et al., 2018) and conscientiousness (Stürmer et al., 2013) have been linked with a higher likelihood of engaging in intergroup contact. These traits, often classified as endeavour‐related traits (Ashton & Lee, 2007), reflect a general disposition to explore novel social environments, navigate unfamiliar situations, and take social risks. Such characteristics appear particularly important in motivating individuals to initiate interactions with members of other ethnic groups, creating opportunities for new cross‐group connections (Stürmer et al., 2013; see also Paolini et al., 2016). By contrast, altruism and cooperation‐related traits, such as agreeableness, honesty‐humility and emotionality do not appear to be so strongly associated with initiating contact, but they may contribute to the quality of intergroup interactions, supporting the development of friendships once contact occurs (see Jackson & Poulsen, 2005; Vezzali et al., 2018).

Social attitudes and ideological orientations constitute another key set of individual‐level predictors of intergroup contact. Research shows that the relationship between intergroup contact and prejudice is bidirectional indicating that not only does intergroup contact reduce prejudice, but also that prejudiced people tend to avoid intergroup contact (Binder et al., 2009; Swart et al., 2011). Similarly, authoritarian or right‐leaning conservative orientations which emphasize tradition, ingroup loyalty and social hierarchy (such as Right‐Wing Authoritarianism and Social Dominance Orientation) have been linked to reduced approach and increased avoidance of intergroup contact (Dhont & Van Hiel, 2009). By contrast, individuals with more egalitarian beliefs and pro‐diversity attitudes are more likely to seek out intergroup interactions. Tropp and Bianchi (2006) showed that valuing diversity predicted greater interest in intergroup contact, particularly among ethnic majority group members. Similarly, Bahns (2017) found that valuing diversity significantly predicted the formation of diverse friendships in terms of race, religion and sexual orientation.

While individual‐level dispositions are important, structural and normative conditions of the local environment also play a critical role in determining opportunities for cross‐group contact. Structural opportunity is a key foundation for contact, with more ethnically diverse neighbourhoods naturally increasing the probability of intergroup interaction (Kotzur & Wagner, 2021; Kros & Hewstone, 2020; Titzmann et al., 2015; Wagner et al., 2006). However, structural opportunity alone does not guarantee contact. Observations of shared social spaces consistently reveal lower levels of racial integration than would be expected by random mixing (McKeown & Dixon, 2017). Such patterns of informal segregation have been documented across diverse contexts, including schools and universities (Al Ramiah et al., 2014; Clack et al., 2005; Orr et al., 2012), public transport (Davis et al., 1966), beaches (Dixon & Durrheim, 2003) and bars and nightclubs (Tredoux & Dixon, 2009).

Beyond demographics, the political and normative climate of a neighbourhood may shape the perceived safety and desirability of intergroup contact. Norms signal prevailing social attitudes and indicate which behaviours are acceptable or expected. At the individual‐level, perceptions of inclusive norms are associated with more frequent and higher‐quality intergroup contact (McKeown & Taylor, 2018; Meleady, 2021; Tropp et al., 2014, 2016). More objective, contextual indicators of the normative environment also appear to matter. Green et al. (2019), for example, explored migration policies across 25 European countries and found that more tolerant country‐level migration policies – serving as a source of social norms – were associated with more intergroup contact engagement among majority group residents. By contrast, contextual signals of intergroup hostility, such as local attacks or terrorist events, are associated with heightened negative attitudes and reduced willingness to engage across group boundaries (Álvarez‐Benjumea & Winter, 2020; van de Vyver et al., 2016).

### Moderating processes: person × context interactions and group‐specific patterns

Importantly, understanding intergroup friendships requires attention not only to individual‐ and contextual‐level predictors in isolation but also to their intersection. While diverse neighbourhoods and supportive local norms may create conditions that make cross‐group interactions possible and socially acceptable, whether these opportunities develop into intergroup friendships likely depends on individual dispositions that shape how people perceive, interpret, and respond to their local environment. One such characteristic is individuals' sense of *identification* or belonging with their local community. Much research in environmental and social psychology has explored place‐based identity, focusing mainly on its impact on personal well‐being, civic engagement, pride in one's local environment, and behaviours that protect or maintain the place (Brown et al., 2002; Harris et al., 1995; Mesch & Manor, 1998; Tartaglia, 2013). More recently, studies have started to examine how a sense of belonging to a neighbourhood can influence intergroup dynamics (Bernardo & Palma‐Oliveira, 2016; Dixon et al., 2022). This work conceptualizes neighbourhood belonging not merely as a psychological attachment to a place, but as a lens through which residents perceive and navigate social relationships within it. For example, Stevenson et al. (2019, 2020, 2021) found that stronger identification with one's local community was associated with reduced intergroup anxiety, more frequent and positive interactions with members of other ethnic or religious groups, and heightened perceptions of the community's collective capacity for constructive intergroup contact.

We suggest that the strength of individuals' neighbourhood identification may not only have a direct effect on intergroup friendships but may also shape how contextual neighbourhood characteristics impact these relations. Residents who feel a strong connection to their neighbourhood are likely to be more attuned to opportunities for engaging with diverse others in their everyday environments and may respond more positively to local norms promoting inclusion and social cohesion. In contrast, residents with weaker neighbourhood ties may be less attuned to these cues, perceiving fewer opportunities or feeling less motivated to act on them. Personality traits may similarly moderate the impact of local diversity on intergroup engagement. Prior research indicates that individuals higher in openness and agreeableness tend to express more positive intergroup attitudes when exposed to diverse environments (Antonoplis & John, 2022; Danckert et al., 2017; Johnston et al., 2015; Silva et al., 2023; Van Assche et al., 2014). Consistently, we may expect individuals high in these traits to be more likely to translate exposure to diverse and inclusive neighbourhoods into intergroup friendships.

It is also important to consider that the predictors of intergroup contact may vary depending on an individual's ethnic group membership. Prior research on the predictors of intergroup contact has concentrated on majority group members' willingness to engage in contact with minorities (Paolini et al., 2018). However, ethnic majority and minority group members may experience and interpret neighbourhood environments differently (Duden et al., 2025; Prati et al., 2022), and the same predictors may have distinct implications across these groups. Strong neighbourhood belonging, for instance, may encourage majority group members to seek cross‐group friendships when they feel secure and included, whereas for minority group members, strong belonging may primarily reflect bonding within co‐ethnic networks, potentially limiting opportunities or motivation for bridging ties (Putnam, 2007). Similarly, contextual factors such as local anti‐immigrant sentiment or normative support for diversity may exert stronger or weaker effects depending on group membership, with majority residents potentially feeling more strongly represented by the societal institutions that communicate those norms and more empowered to act on them (Kauff et al., 2020). We therefore examine not only how individual‐ and contextual‐level predictors interact to shape intergroup friendships, but also how these influences vary between ethnic groups, highlighting the complex, multilevel dynamics that influence intergroup contact engagement.

### The present research

The present study leverages a large, nationally representative UK longitudinal panel (Understanding Society, Institute for Social and Economic Research, 2024) to examine the role of individual‐ and contextual‐level factors in fostering intergroup friendships. The Understanding Society dataset included repeated measures of intergroup friendship at three timepoints over six years. The dataset combines psychological and socio‐demographic variables with fine‐grained geographic identifiers, allowing individuals to be linked to their local neighbourhood context. The sample includes both ethnic majority and minority participants, with an ethnic minority boost that allows for robust comparisons across groups.

**FIGURE 1:**
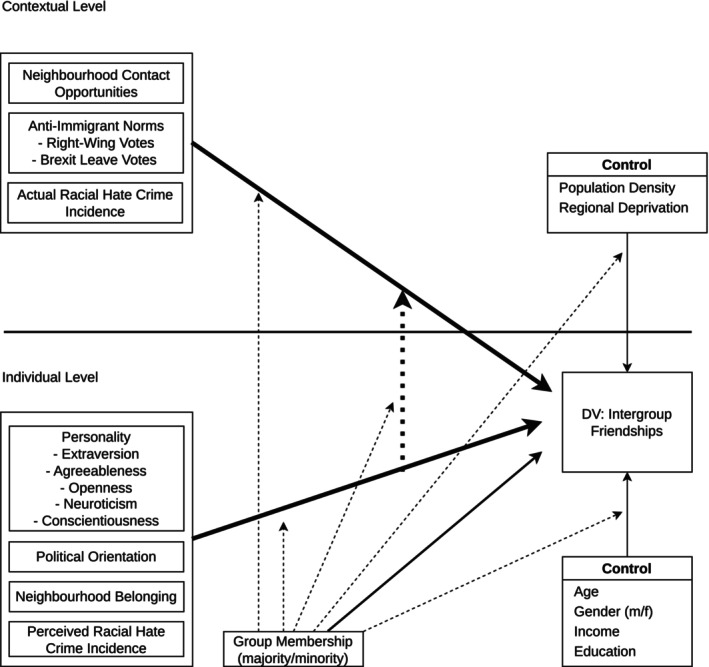
Conceptual overview of the multilevel model. For ease of interpretation, the conceptual figure shows the outcome and the individual‐ and contextual‐level predictors only; the longitudinal, time‐varying aspect of the data is not depicted.

We examined multiple individual‐level psychological factors as predictors of intergroup friendships, alongside objective indicators of participants' neighbourhood environment. At the individual‐level, we focused on personality traits, political orientation, neighbourhood belonging, and perceived local racial crime incidence. To capture neighbourhood‐level influences, we incorporated measures of neighbourhood ethnic composition—reflecting structural opportunities for intergroup contact—as well as broader markers of local intergroup climate, including police‐recorded data on the actual racial hate crime prevalence and local anti‐immigration norms (indexed by right‐wing party vote shares in general elections and the Brexit referendum Leave vote share). In addition to testing main effects of our predictor variables, we examined cross‐level interactions to assess whether individual psychological characteristics moderate the influence of structural opportunities and normative climates on intergroup friendships, as well as group‐level interactions to determine whether predictors operate differently for ethnic majority and minority group members. A conceptual overview of the modelling framework is provided in Figure 1.

## METHODS

### Participants

Understanding Society uses a clustered and stratified, probability sample (i.e., random selection of households in pre‐defined areas), as well as an ethnic minority boost sample of households from areas of high ethnic minority concentration (see Lynn, 2009 for more information on the sampling procedures of Understanding Society). Households are visited yearly for an in‐person interview. For the current project, we analysed at three waves of data (T1: wave 3; T2: wave 6; T3: wave 9^1^) which each included the measure of our dependent variable, cross‐group friendships. We excluded participants who did not have data on our dependent variable on any of the three waves. This left us with a maximum possible sample of 54,626 participants^2^ between 16 and 103 years of age at T1 (*M*_age_ = 47.66, *SD* = 18.23). 54.99% of the sample was female and the majority of participants (84.58%) identified as White (at T1).

We selected participants who identified with the ethnic majority group (White) or who identified with one of the two largest ethnic minority groups (Asian 10.96% or Black 4.55%). Participants identifying with other ethnic groups were excluded from the main analyses due to very small sample sizes – only 1.81% identified as ‘Mixed’ and 0.80% as ‘Other’ making reliable statistical comparisons impractical. For the main analyses, we pooled participants into two categories; ethnic majority (White), and minority (all ethnic minority participants combined). This approach facilitates clear comparisons between majority and minority group experiences, simplifies interpretation, and aligns with common practices in the field when examining broad majority‐minority dynamics (see Tropp & Pettigrew, 2005). A more detailed three‐group model (White, Black, and Asian) is presented in the Supporting Information for the interested reader.

Participants were nested within Lower Super Output Areas (LSOAs), which are nested within Local Authority Districts (LADs). LSOAs are standardized geographical regions comprising 400–1200 households. LADs are a higher order division which also include governmental and administrative functions (ranging between small rural districts of roughly 25,000 households to large metropolitan areas exceeding 400,000 households).

Missing data reduced the size of the final analytic sample. Around 29% of data points were missing, mostly due to non‐participation at one or more survey waves (23% of observations). All models were fitted with Restricted Maximum Likelihood estimation (REML) using lme4 (Bates et al., 2015) in R. The data are structured in long form with one row per observation per individual. Thus, where individuals are missing data at one or more waves, they are not listwise deleted from the estimation sample; rather, only those observations with missing values are excluded. The final analytic sample therefore comprised 18,807 participants contributing 42,431 observations. Given the large sample size even after accounting for missingness, small effects (*β* < .20) could still be estimated with 80% power^3^ Participants were distributed across 11,181 different LSOAs and 320 different LADs reflecting substantial geographic coverage and variation within the sample.

### Procedure and measures

Understanding Society is very well documented and more information on the variables and procedures, including obtaining informed consent can be found on their website (https://www.understandingsociety.ac.uk/).

#### Dependent variable

##### Intergroup friendships

Participants were asked to indicate the proportion of their friends that are of the same‐ethnic group as themselves, ranging from 1 (all the same) to 4 (less than half). Higher scores indicated more intergroup friendships. This variable was assessed at all three time points. The question was identical for ethnic majority and ethnic minority participants, therefore, for minority group members, higher scores may reflect friendships with both ethnic majority members and members of other minority groups.

#### Individual‐level predictors

##### Personality

The Big 5 personality dimensions (agreeableness, openness, extraversion, neuroticism, and conscientiousness) were assessed with three items each based on the questionnaire by John and Srivastava (1999). The items for each personality dimension were averaged and the final scores range between 1 (does not apply to me at all) to 7 (applies to me perfectly). Reliability in the current sample ranged between *α* = .54 (conscientiousness) to *α* = .70 (neuroticism). Each dimension was assessed at T1 only and is treated as time‐invariant.

##### Political orientation

Political orientation was used as an indicator of individual ideology, with left‐leaning views generally reflecting stronger support for egalitarianism and diversity, and right‐leaning views reflecting a greater emphasis on tradition and ingroup loyalty. Participants rated how much they liked the Labour Party and the Conservative Party on a scale from 0 (strongly dislike) to 10 (strongly like). We calculated a difference score by subtracting Labour liking from Conservative liking, so higher scores indicate a preference for the Conservative Party and a more conservative ideological orientation. This variable was measured at T1 and T2 only, and the mean across the two timepoints was used as a time‐invariant predictor in our analyses to avoid loss of data. The correlation between T1 and T2 was high (*r* = .75), indicating that political orientation is fairly stable across timepoints.

##### Neighbourhood belonging

Neighbourhood belonging was measured with 8 items capturing people's sense of connection, inclusion and support within their local community. Example items include ‘I feel like I belong to this neighbourhood’ and ‘I think of myself as similar to the people that live in this neighbourhood’. The items were averaged and range between 1 (low belonging) to 5 (high belonging). The measure had a good reliability (*α* = .88 at T1). This variable was assessed at all three time points and is treated as time‐varying.

##### Perceived racial hate crime incidence

Participants were asked how common it is that insults or attacks in their local area are due to someone's race or colour. The item ranged from 1 (very common) to 4 (not common at all) and was assessed at all three time points. We reverse coded this item so that higher scores reflect greater perceived local intergroup hostility. This variable was assessed at all three time points and is treated as time‐varying.

#### Contextual‐level predictors

##### Contact opportunities

Using data from the 2011 Census, we calculated, for each participant, the proportion of individuals in their LSOA who are of the same‐ethnic group as they are. This approach was chosen to align with the dependent variable: for majority group members, cross‐group contact entails contact with ethnic minority group members, whereas for minority group members, cross‐group contact may include both the majority group and other minority groups. By calculating the proportion of same‐ethnic group members, we can infer the relative availability of outgroup members: a lower proportion of same‐ethnic residents indicates a higher potential for contact, while a higher proportion indicates fewer opportunities for cross‐group interaction. Using general diversity indices, such as Simpson's index, would capture overall heterogeneity in the area but would not indicate how many people belong to the participant's own ethnic group. Consequently, such indices are less informative about the number of available outgroup contacts for any given individual, making them less directly comparable to a measure focused on cross‐ethnic interactions. This variable was assessed at a single time point (2011), corresponding to T1, and was treated as time‐invariant in the analyses.

##### Anti‐immigration norms

We used two voting‐based indicators as objective indicators of local anti‐immigrant/ diversity norms. First, drawing on general election data from 2010, 2015, and 2017, we aggregated the vote share for the two most prominent right‐wing parties at the time — the British National Party (BNP) and the United Kingdom Independence Party (UKIP) – at the constituency level. These vote shares have been used in previous research as indicators of local anti‐immigration sentiment and exclusionary political attitudes (Kaufmann, 2017; Kaufmann & Harris, 2015). Second, we incorporated the 2016 Brexit referendum vote share for Leave (at the LAD level) which likewise captured stronger local anti‐immigrant norms. Voting data were obtained from https://www.data.gov.uk/

##### Actual racial hate crime incidence

Using UK police data (obtained from https://www.data.gov.uk/) we also measured the number of racial based hate crimes per Police Force Area. This variable served as an objective, contextual indicator of intergroup hostility. We selected data from 2010, 2015, 2017 to match the general election data, and treat it as time‐varying.

#### Control variables

Within all our analyses, we included control variables at both the individual‐ and contextual‐levels to reduce potential confounding. At the individual‐level, we controlled for age (in years), gender (male/female), personal income (GBPs per month) and education level (1 = secondary education, 2 = post‐secondary education, and 3 = higher education). These socio‐demographic factors were included because they are plausibly related to both our psychological predictor variables (e.g., political orientation, neighbourhood belonging, see for example: Boschman, 2018; Furnham & Fenton‐O'Creevy, 2018) and the likelihood of engaging in intergroup friendships (Ceobanu & Escandell, 2010; Damen et al., 2021; Hello et al., 2006; van Tubergen, 2015). At the contextual‐level, we controlled for population density (number of people per hectare in each LSOA) and regional deprivation (LSOAs ranked in quintiles using the Index of Multiple Deprivation, IMD) to account for local‐area characteristics that may influence the distribution of our contextual predictors (e.g., neighbourhood ethnic composition, racial hate crime incidence; see for example Belgioioso et al., 2023; Wickes et al., 2013) and the probability of forming intergroup friendships (Abascal & Baldassarri, 2015; Bynner, 2019; Letki, 2008). Controlling for these factors helps to rule out spurious associations between our key predictors and intergroup friendships, thereby providing a more accurate estimate of the relationships of interest. Comparable control variables are commonly included in multivariate studies of intergroup contact (Christ et al., 2014; Green et al., 2019; Kros & Hewstone, 2020; Laurence et al., 2018).

### Analytic strategy

We ran multilevel models using lme4 (Bates et al., 2015) in R (R Core Team, 2024). Analyses were pre‐registered at https://osf.io/9bnt6/?view_only=874225779b664ba88ae9d9b2bc21117f.^4^ Our model examined individual‐ and contextual‐level predictors of cross‐group friendships across three timepoints. It includes both time‐varying (i.e., assessed at all three timepoints) and time‐invariant (i.e., assessed at only one timepoint) variables on both the individual‐ and contextual‐level. Repeated observations were nested within individuals, and individuals were further nested within geographical units. We specified random intercepts at the participant level, at the LSOA level (small geographical nesting unit), and for the LAD level (overarching geographical nesting unit). This structure allows the model to estimate how within‐person and within‐area changes in predictors over time relate to changes in cross‐group friendship while controlling for stable differences across individuals and areas. The selection of geographical levels was based on the ICC as well as model parsimony (see Appendix S1 for more details). The dependent variable, intergroup friendship, was treated as continuous in the multilevel models. Supplementary analyses using cumulative link mixed models (CLMM) produced comparable results, indicating robustness to the modelling approach (see Appendix S2). All analyses were conducted with standardized variables and included individual‐level and contextual‐level control variables.

The model was built in a stepwise fashion: starting with main effects, then adding cross‐level interactions, examining majority and minority groups separately, and finally including group interactions. The model reported here is the fullest specification, incorporating all covariates, main effects, and interactions (for the partial models, please see Appendix S3). The cross‐level interactions test whether the relationship between individual‐level predictors and intergroup friendship varies depending on contextual‐level characteristics. We conceptualize these interactions with the individual‐level predictor as the moderator, either strengthening (i.e., steeper slope) or weakening (i.e., less steep slope) the relation between the contextual‐level characteristics and intergroup friendships. By including interactions between each predictor and group membership, we also tested whether the effects of individual‐ and contextual‐level predictors differ significantly between majority and minority group members. Because main effects are estimated with the majority group as the reference, they describe predictors for majority members. Minority‐group effects equal the main effect plus the relevant group × predictor interaction. Non‐significant interactions indicate similar effects across groups, allowing cautious generalization. This approach tests overall predictors while explicitly assessing group differences. Full group‐specific estimates as pre‐registered are provided in the Appendix S3.^5^ Three‐way interactions between individual‐ and contextual‐level predictors with group membership (minority/majority) are also reported in Appendix S4.

To complement the multilevel analyses and further unpack the temporal dynamics observed, we also explored potential bidirectional relationships between intergroup friendships and key time‐varying individual‐level predictors that emerged as significant in the multilevel model. Specifically, we estimated Random‐Intercept Cross‐Lagged Panel Models (RI‐CLPMs, Hamaker et al., 2015) using *lavaan* (Rosseel, 2012) in R. RI‐CLPMs extend traditional cross‐lagged models by separating stable between‐person differences from within‐person fluctuations. Latent random intercepts were specified for each significant time‐varying predictor and for intergroup friendship; their covariances capture whether individuals who, on average, score higher on a given predictor also tend to report more intergroup friendships. Within‐person latent factors represent each person's deviation from their own mean at each wave. Autoregressive and cross‐lagged paths among these factors reveal whether deviations at one timepoint predict deviations at the next, providing a test of pure within‐person effects. Whereas the multilevel model considers multiple predictors simultaneously, including cross‐level interactions, the RI‐CLPM isolates the role of each significant time‐varying predictor, disentangling between‐ and within‐person processes. This approach allows us to assess whether changes in a given predictor over time are associated with subsequent changes in intergroup friendship—or vice versa—while accounting for each individual's stable baseline.

## RESULTS

### Individual‐ and contextual‐level predictors of intergroup friendship: multilevel model

Table 1 presents the correlations among the main study variables at T1 (corresponding correlations at T2 and T3 are similar and can be found in the Appendix S5). Given the large sample size, correlations as small as ±.02 reached statistical significance, resulting in nearly all variables being significantly correlated. Results of the full multilevel models can be found in Table 2 (see Appendix S4 for the complete results table, including interactions between control variables and focal variables). In these models, majority group members serve as the reference category for estimating main effects, effects therefore represent estimates for majority group members at average levels of the other covariates. To account for multiple testing, we focus on interpreting only effects significant at *α* < .01. As our variables were standardized, model estimates represent standardized coefficients. On the individual‐level, people who tended to score higher on openness (*β* = .050, *p* < .001) and agreeableness (*β* = .019, *p* = .010) tended to have more intergroup friendships. In addition, a preference for the Labour party (*β* = −.030, *p* < .001) and higher perceived racial hate crime prevalence (*β* = .026, *p* < .001) were also related to more intergroup friendships. Furthermore, neighbourhood‐belonging (*β* = .022, *p* < .001) was related to more intergroup friendships for majority group members in the multilevel model.^6^ On the contextual‐level, the strongest predictor was contact opportunities, with living in more diverse areas having more intergroup friendships (*β* = .290, *p* < .001). In addition, participants living in areas with higher anti‐immigrant norms—reflected by a higher percentage of Brexit ‘Leave’ votes—reported fewer intergroup friendships (*β* = −.044, *p* = .002). Finally, living in areas with higher incidence of racial hate crimes predicted more intergroup friendships (*β* = .041, *p* = .001).

**TABLE 1:** Pearson correlation table of focal predictors at T1.

		*M*	*SD*	1	2	3	4	5	6	7	8	9	10	11	12	13
	1 Intergroup friendships	1.77	0.89													
Individual‐level predictors																
	2 Openness	4.71	1.25	.**08**												
	3 Agreeableness	5.60	1.02	.01	.**19**											
	4 Extraversion	4.63	1.30	.**03**	.**24**	.**15**										
	5 Neuroticism	3.59	1.41	−.01	**−.11**	**−.07**	**−.18**									
	6 Conscientiousness	5.47	1.07	−.01	.**18**	.**30**	.**18**	**−.17**								
	7 Political Orientation	−0.43	3.70	**−.09**	**−.03**	**−.02**	.01	**−.06**	.**07**							
	8 Neighbourhood Belonging	3.52	0.71	**−.05**	.**05**	.**18**	.**16**	**−.14**	.**16**	.**06**						
	9 Perceived Racial Hate Crime Incidence	1.27	0.51	.**11**	.00	**−.02**	−.01	.**05**	**−.06**	**−.11**	**−.11**					
Contextual‐level predictors																
	10 Contact Opportunities	0.16	0.24	.**37**	.**02**	.**02**	**−.05**	**−.03**	**−.03**	**−.15**	**−.08**	.**22**				
	11 Brexit Leave Vote	0.53	0.10	**−.18**	**−.08**	.**02**	.01	.**02**	.**02**	.**06**	.01	**−.08**	**−.35**			
	12 Right‐Wing Votes	0.05	0.02	**−.11**	**−.05**	.**02**	.01	.01	.01	.**02**	−.01	**−.02**	**−.21**	.**69**		
	13 Actual Racial Hate Crimes	1950.41	2558.19	.**25**	.**03**	−.01	**−.03**	−.01	**−.04**	**−.09**	**−.04**	.**17**	.**57**	**−.50**	**−.30**	

*Note*: *n* = 18,488; boldface correlations indicate statistical significance at *α* ≤ .05.

**TABLE 2:** Multilevel predictors of cross‐group friendship: fixed effects, cross‐level interactions, and group differences (see Appendix S4 for full table, including control variables).

	*β*	*SE*	*t*	*p*
Intercept	.154	0.017	8.881	<.001
Time	−.003	0.005	−0.503	.615
Group membership (ref. majority)	.069	0.075	0.919	.358
Individual‐level				
Extraversion	.006	0.007	0.787	.431
Agreeableness	.019	0.007	2.583	.010
Openness	.050	0.008	6.622	<.001
Neuroticism	−.015	0.007	−2.071	.038
Conscientiousness	.007	0.008	0.858	.391
Preference Conservative Party	−.030	0.007	−4.232	<.001
Neighbourhood Belonging	.022	0.006	3.567	<.001
Perceived Racial Hate Crime Incidence	.026	0.006	4.674	<.001
Contextual‐level				
Contact Opportunities	.290	0.019	15.158	<.001
Actual Racial Hate Crime Incidence	.041	0.012	3.458	.001
Right‐Wing Votes	.003	0.011	0.300	.765
Brexit Leave Votes	−.044	0.014	−3.131	.002
Cross‐level interactions				
Contact opportunities				
× Extraversion	−.023	0.015	−1.479	.139
× Agreeableness	.029	0.015	1.976	.048
× Openness	−.032	0.015	−2.088	.037
× Neuroticism	−.016	0.015	−1.075	.282
× Conscientiousness	.039	0.016	2.443	.015
× Preference Conservative Party	.019	0.014	1.366	.172
× Neighbourhood Belonging	.041	0.013	3.260	.001
× Perceived Racial Hate Crime Incidence	−.016	0.010	−1.580	.114
Actual Racial Hate Crimes Incidence				
× Extraversion	.010	0.009	1.151	.250
×Agreeableness	−.010	0.009	−1.104	.270
× Openness	−.001	0.009	−0.148	.883
× Neuroticism	.009	0.009	1.004	.316
× Conscientiousness	−.010	0.009	−1.055	.291
× Preference Conservative Party	−.009	0.008	−1.107	.268
× Neighbourhood Belonging	.013	0.008	1.708	.088
× Perceived Racial Hate Crime Incidence	.007	0.007	1.005	.315
Right‐Wing Votes				
× Extraversion	−.004	0.009	−0.450	.652
× Agreeableness	.001	0.009	0.074	.941
× Openness	.011	0.009	1.293	.196
× Neuroticism	−.012	0.009	−1.365	.172
× Conscientiousness	−.016	0.009	−1.708	.088
× Preference Conservative Party	.015	0.009	1.680	.093
× Neighbourhood Belonging	−.005	0.008	−0.639	.523
× Perceived Racial Hate Crime Incidence	.003	0.007	0.483	.629
Brexit Leave Votes				
× Extraversion	−.012	0.010	−1.221	.222
× Agreeableness	−.014	0.010	−1.441	.150
× Openness	.002	0.010	0.172	.864
× Neuroticism	.018	0.009	1.959	.050
× Conscientiousness	.035	0.010	3.358	.001
× Preference Conservative Party	−.002	0.009	−0.163	.871
× Neighbourhood Belonging	.014	0.008	1.686	.092
× Perceived Racial Hate Crime Incidence	−.004	0.008	−0.480	.632
Two‐way Group membership interactions				
Group (ref. Majority) × individual‐level				
× Extraversion	.060	0.051	1.178	.239
× Agreeableness	.030	0.047	0.640	.522
× Openness	−.004	0.052	−0.086	.931
× Neuroticism	.008	0.047	0.160	.873
×Conscientiousness	.007	0.047	0.158	.875
× Preference Conservative Party	.033	0.047	0.701	.483
× Neighbourhood Belonging	−.161	0.040	−3.992	<.001
× Perceived Racial Hate Crime Incidence	−.033	0.028	−1.162	.245
Group (ref. Majority) × contextual‐level				
× Contact Opportunities	−.027	0.031	−0.886	.375
× Actual Racial Hate Crime Incidence	−.063	0.019	−3.337	.001
× Right‐Wing Votes	−.068	0.027	−2.471	.013
× Brexit Leave Votes	−.015	0.032	−0.463	.643

Variance components	Variance		*R* ^2^	
Random Intercept LAD	.016			
Random Intercept LSOA	.031			
Random Intercept Participant	.234			
Residual	.545			
Random effects (combined)			.447	
Fixed effects			.162	

*Note*: *n* observations = 42,430; *n* individual‐level = 18,806; *n* LSOA level = 11,181; *n* LAD level = 320; all estimates are based on standardized regression coefficients; the model included control variables as well as the three‐way interactions between group memberships, individual‐level predictors and contextual‐level predictors, the results of which can be found in the Supporting Information.

**FIGURE 2:**
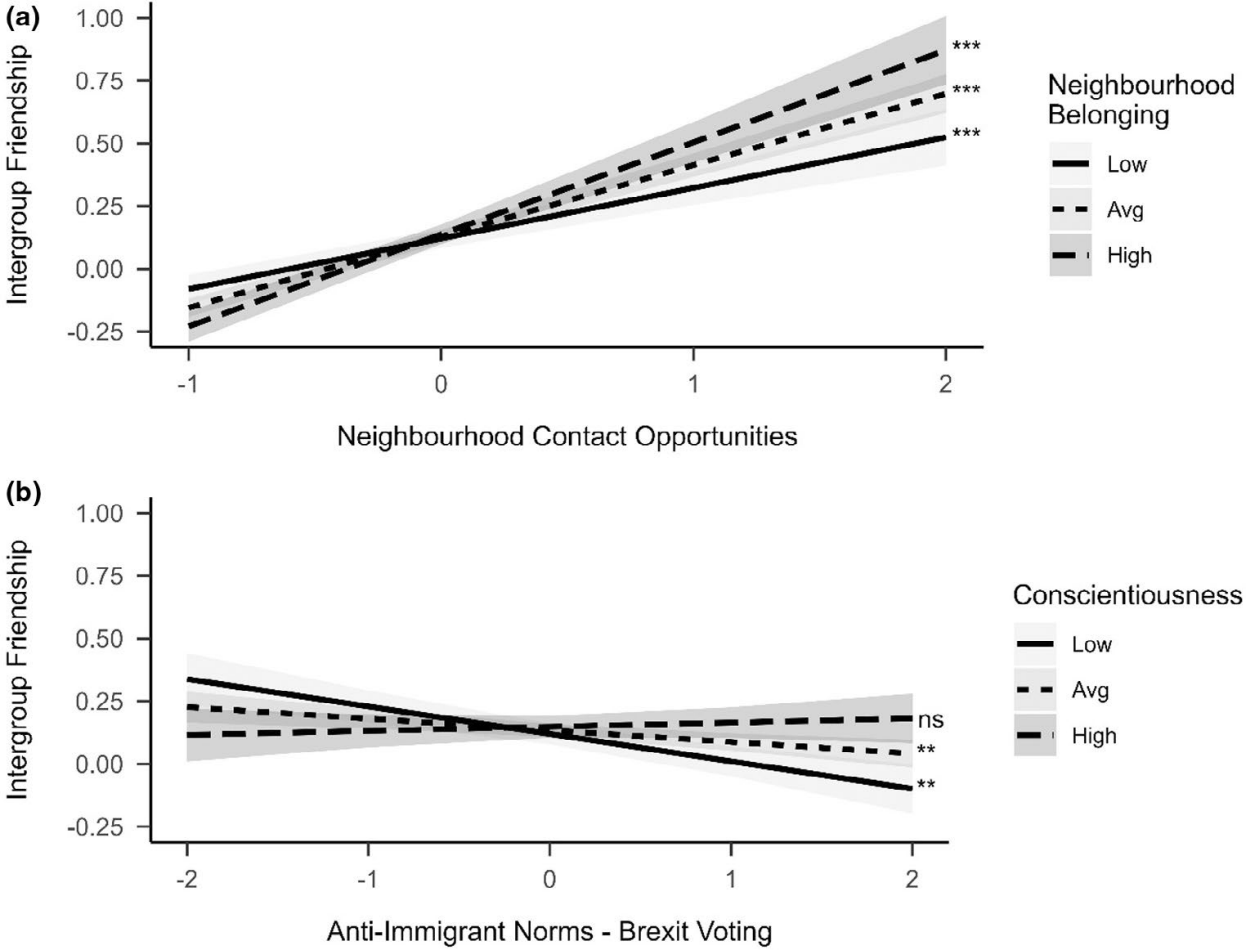
Cross‐level interactions predicting intergroup friendships from a combination of individual‐ and contextual‐level predictors. The *x*‐axis shows standardized predictor values, and the *y*‐axis shows model‐predicted intergroup friendship scores. Significance indicates whether slopes are significantly different from 0 at *p < .05, **p < .01, ***p < .001.

In addition, there were some significant cross‐level interactions between individual‐ and contextual‐level predictors (see Figure 2). There was a significant interaction between neighbourhood belonging and neighbourhood contact opportunities (*β* = .041, *p* = .001). Specifically, the association between neighbourhood contact opportunities and intergroup friendships was stronger for individuals higher in neighbourhood belonging (Figure 2, panel a). Simple slope analyses of high (+2*SD; β* = .360, 95% CI [0.309, 0.411]), average (*M; β* = .275, 95% CI [0.246, 0.304]), and low (−2*SD*; *β* = .190, 95% CI [0.144, 0.237]) neighbourhood belonging were all significantly different from 0. This finding suggests that while contact opportunities matter broadly, individuals higher in neighbourhood belonging are particularly likely to engage in intergroup interactions when opportunities arise. There was also a significant interaction between conscientiousness and local anti‐immigrant norms – measured via Brexit voting (*β* = .035, *p* = .001). Simple slopes indicated that the influence of anti‐immigrant norms and intergroup friendships was significant only for individuals with low (*β* = −.090, 95% CI [−0.152, −0.029]) and average (*β* = −.055, 95% CI [−0.091, −0.019]) levels of conscientiousness but not for those with high conscientiousness (*β* = −.020, 95% CI [−0.084, 0.044]; Figure 2, panel b).

**FIGURE 3:**
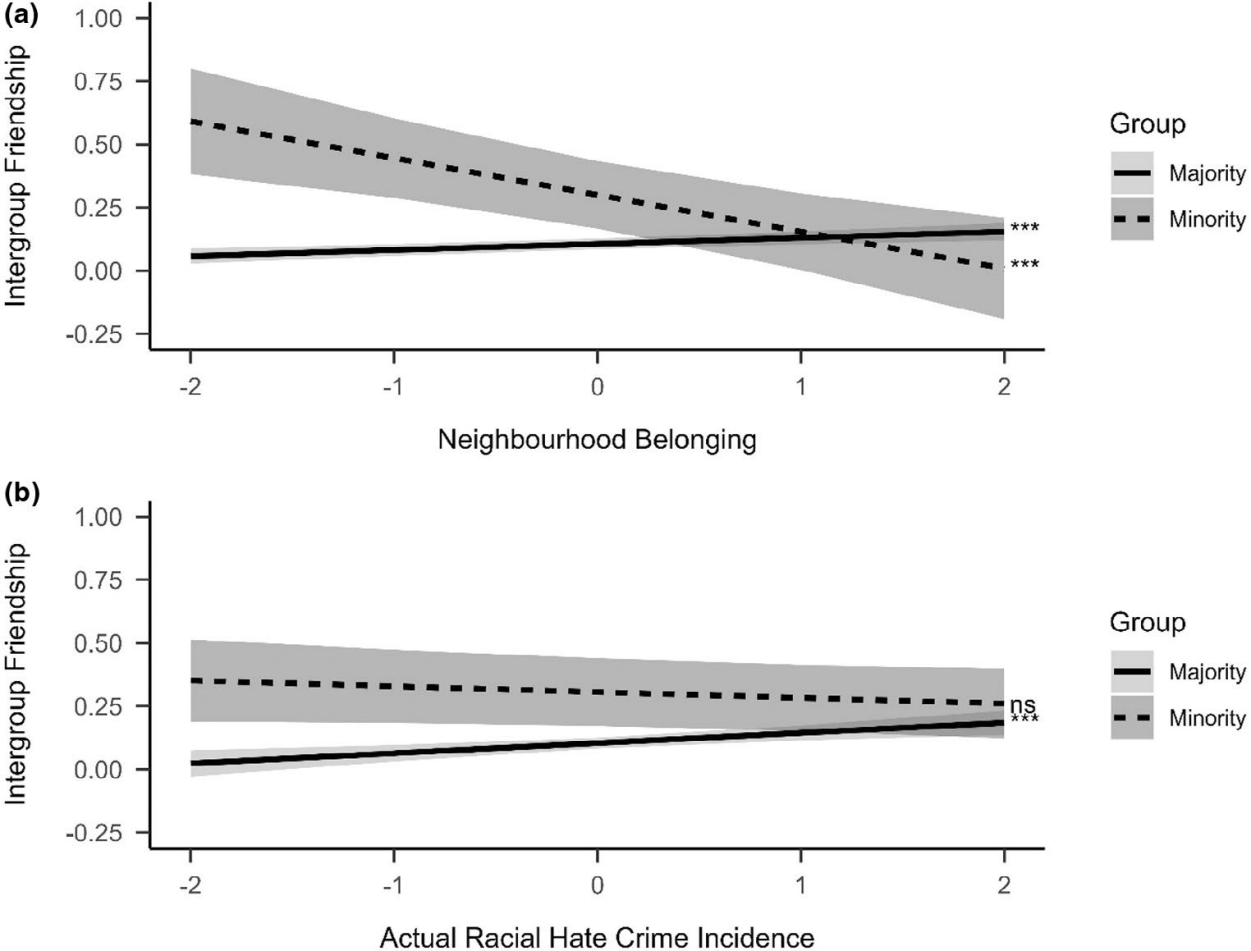
Group‐by‐predictor interactions on intergroup friendships. The *x*‐axis shows standardized predictor values, and the *y*‐axis shows model‐predicted intergroup friendship scores. Significance indicates whether slopes are significantly different from 0 at ***p < .001.

When looking at the differences between majority and minority group members, a number of significant two‐way interactions occurred with group membership (Figure 3). First, the relation between neighbourhood belonging and intergroup friendships differed between majority and minority group members (*β* = −.161, *p* < .001, Figure 3, panel a). Simple slopes examination indicated that whereas higher neighbourhood belonging was significantly related to more intergroup friendships for majority group members (*β* = .024, 95% CI [0.012, 0.036]), the opposite emerged for minority group members (*β* = −.145, 95% CI [−0.223, −0.067]): higher neighbourhood belonging was significantly related to less intergroup friendships for minority group members. Additionally, the relation between actual local hate crime incidence and intergroup friendships differed between groups (*β* = −.063, *p* = .001). While higher objective local racial hate crime incidence was significantly related to more intergroup friendships for majority group members (*β* = .040, 95% CI [0.018, 0.063]), it was unrelated to the outcome for minority group members (*β* = −.023, 95% CI [−0.058, 0.011]; Figure 3, panel b).

There were some three‐way interactions between individual‐ and contextual‐level with group membership. We report on these in the Appendix S4.

### Time‐varying predictors of intergroup friendship: random intercept cross‐lagged panel models

According to the multilevel model, two time‐varying individual‐level predictors were significantly associated with intergroup friendship: neighbourhood belonging and perceived local racial hate crime prevalence. To examine whether there are potential bidirectional links, we ran a two‐group Random Intercept Cross‐Lagged Panel Model (RI‐CLPM, see Table 3). We included the T1 control variables as time‐invariant predictors of the random intercepts (i.e. the between‐person variables). We furthermore included cluster‐robust standard errors to account for the geographical dependence at the LSOA level. The model fit the data well (robust fit indices: CFI = .995, TLI = .989, RMSEA = .015, SRMR = .010).^7^ For majority group members, on a within‐person level, there were no significant cross‐lagged paths with intergroup friendship, indicating that changes from a person's average levels of intergroup friendship are not followed or preceded by changes from a person's average in neighbourhood belonging or perceived local racial hate crime prevalence. For minority group members, at the within‐person level, cross‐lagged paths with intergroup friendship were also non‐significant. At the between‐person level, intergroup friendships were positively associated with perceived racial hate crime prevalence for majority group members,^8^ whereas for minority group members, there was a significant negative correlation between intergroup friendships and neighbourhood belonging.

**TABLE 3:** Random‐intercept cross‐lagged panel model estimates for intergroup friendships, neighbourhood belonging, and perceived racial hate crime incidence.

		*β*	*SE*	*z*	*p*
Majority group					
Between‐person differences					
	RI Intergroup Friendships ↔ RI Neighbourhood Belonging	.020	0.005	0.842	.400
	RI Intergroup Friendships ↔ RI Perceived Racial Hate Crime Incidence	.189	0.003	4.283	<.001
	RI Neighbourhood Belonging ↔ RI Perceived Racial Hate Crime Incidence	085	0.003	.251	.024
Within‐person autocorrelations					
	T1 Intergroup Friendship → T2 Intergroup Friendship	.078	0.021	3.795	< .001
	T2 Intergroup Friendship → T3 Intergroup Friendship	.115	0.021	4.968	< .001
	T1 Neighbourhood Belonging → T2 Neighbourhood Belonging	.166	0.034	5.159	< .001
	T2 Neighbourhood Belonging → T3 Neighbourhood Belonging	.219	0.027	9.058	< .001
	T1 Perceived Racial Hate Crime Incidence → T2 Perceived Racial Hate Crime Incidence	.015	0.026	0.520	.603
	T2 Perceived Racial Hate Crime Incidence → T3 Perceived Racial Hate Crime Incidence	.031	0.030	1.112	.266
Within‐person cross‐lags					
	T1 Neighbourhood Belonging → T2 Intergroup Friendship	−.020	0.030	−0.995	.320
	T2 Neighbourhood Belonging → T3 Intergroup Friendship	−.002	0.023	−0.103	.918
	T1 Perceived Racial Hate Crime Incidence → T2 Intergroup Friendship	.006	0.024	0.410	.682
	T2 Perceived Racial Hate Crime Incidence → T3 Intergroup Friendship	.002	0.031	0.091	.928
	T1 Intergroup Friendship → T2 Neighbourhood Belonging	.019	0.011	1.150	.250
	T2 Intergroup Friendship → T3 Neighbourhood Belonging	.006	0.013	0.373	.709
	T1 Intergroup Friendship → T2 Perceived Racial Hate Crime Incidence	.021	0.010	1.224	.221
	T2 Intergroup Friendship → T3 Perceived Racial Hate Crime Incidence	.013	0.010	0.745	.456
	T1 Neighbourhood Belonging → T2 Perceived Racial Hate Crime Incidence	−.044	0.021	−1.752	.080
	T2 Neighbourhood Belonging → T3 Perceived Racial Hate Crime Incidence	−.027	0.017	−1.375	.169
	T1 Perceived Racial Hate Crime Incidence → T2 Neighbourhood Belonging	−.014	0.020	−0.836	.403
	T2 Perceived Racial Hate Crime Incidence → T3 Neighbourhood Belonging	−.032	0.026	−1.672	.094
Minority group					
Between‐person differences					
	RI Intergroup Friendships ↔ RI Neighbourhood Belonging	−.160	0.016	−3.256	.001
	RI Intergroup Friendships ↔ RI Perceived Racial Hate Crime Incidence	−.024	0.015	−0.351	.726
	RI Neighbourhood Belonging ↔ RI Perceived Racial Hate Crime Incidence	−.025	0.011	−0.332	.740
Within‐person autocorrelations					
	T1 Intergroup Friendship → T2 Intergroup Friendship	−.006	0.065	−0.095	.924
	T2 Intergroup Friendship → T3 Intergroup Friendship	.150	0.055	2.744	.006
	T1 Neighbourhood Belonging → T2 Neighbourhood Belonging	.060	0.075	0.776	.438
	T2 Neighbourhood Belonging → T3 Neighbourhood Belonging	.075	0.072	1.208	.227
	T1 Perceived Racial Hate Crime Incidence → T2 Perceived Racial Hate Crime Incidence	−.049	0.063	−0.712	.477
	T2 Perceived Racial Hate Crime Incidence → T3 Perceived Racial Hate Crime Incidence	−.003	0.079	−0.035	.972
Within‐person cross‐lags					
	T1 Neighbourhood Belonging → T2 Intergroup Friendship	−.004	0.077	−0.067	.947
	T2 Neighbourhood Belonging → T3 Intergroup Friendship	−.023	0.074	−0.469	.639
	T1 Perceived Racial Hate Crime Incidence → T2 Intergroup Friendship	−.052	0.057	−1.171	.242
	T2 Perceived Racial Hate Crime Incidence → T3 Intergroup Friendship	−.003	0.070	−0.063	.949
	T1 Intergroup Friendship → T2 Neighbourhood Belonging	.051	0.037	0.970	.332
	T2 Intergroup Friendship → T3 Neighbourhood Belonging	−.005	0.039	−0.099	.921
	T1 Intergroup Friendship → T2 Perceived Racial Hate Crime Incidence	−.019	0.041	−0.344	.731
	T2 Intergroup Friendship → T3 Perceived Racial Hate Crime Incidence	−.046	0.035	−0.984	.325
	T1 Neighbourhood Belonging → T2 Perceived Racial Hate Crime Incidence	−.042	0.060	−0.714	.475
	T2 Neighbourhood Belonging → T3 Perceived Racial Hate Crime Incidence	.027	0.061	0.486	.627
	T1 Perceived Racial Hate Crime Incidence → T2 Neighbourhood Belonging	−.050	0.043	−1.020	.308
	T2 Perceived Racial Hate Crime Incidence → T3 Neighbourhood Belonging	−.047	0.061	−0.859	.390

*Note*: Robust fit indices: CFI = .995, TLI = .989, RMSEA = .015, SRMR = .010; *n* participants = 18,488; cluster‐robust standard errors for the LSOA level were included.

## DISCUSSION

The present study examined how individual‐ and contextual‐level factors jointly predict cross‐ethnic friendships in a nationally representative sample of UK adults over time, using three waves of longitudinal data from the Understanding Society panel. By integrating multilevel and longitudinal approaches, we were able to explore not only the main predictors of intergroup friendships but also the ways in which the predictors of interethnic friendship interact across levels and differ between ethnic majority and minority group members. Our findings provide nuanced insights into the complex, multidetermined processes shaping intergroup friendships.

Consistent with expectations, individual dispositions mattered. Openness, an endeavour‐related trait, emerged as a robust predictor of intergroup friendship. Conscientiousness and extraversion were unrelated to intergroup friendship, suggesting that while these traits may facilitate initial contact (Stürmer et al., 2013; Turner et al., 2014), they do not necessarily predict the deeper, sustained intergroup ties captured by our outcome variable. By contrast, agreeableness, a cooperation‐oriented trait, positively predicted cross‐group friendships, consistent with prior evidence linking it to higher‐quality intergroup contact (Vezzali et al., 2018). Political orientation was also related to intergroup friendships, with individuals with more left‐leaning preferences reporting more cross‐ethnic friendships. This finding aligns with research linking egalitarian beliefs and pro‐diversity attitudes to intergroup engagement (Bahns, 2017; Tropp & Bianchi, 2006), suggesting that left‐leaning individuals may be more inclined to form friendships across ethnic boundaries.

At the contextual‐level, structural opportunity remained paramount. Living in neighbourhoods with a higher proportion of other ethnic residents—indicating greater potential outgroup contact – had more cross‐ethnic friendships (Kotzur & Wagner, 2021; Kros & Hewstone, 2020; Titzmann et al., 2015; Wagner et al., 2006). An objective measure of local anti‐immigration norms also predicted prevalence of cross‐group friendships. Individuals residing in areas with lower percentages of Brexit ‘Leave’ votes reported more intergroup friendships. Interestingly, right‐wing voting, as indicated by aggregated BNP and UKIP support, was unrelated to the prevalence of intergroup friendships. This may reflect the marginal and fluctuating nature of these parties' support across elections, making them a less salient marker of local norms. The Brexit vote, by contrast, was a single, highly visible event that captured widespread public attention and served as a salient barometer of prevailing social attitudes towards diversity and inclusion (Meleady et al., 2017).

Importantly, a central contribution of this study lies in demonstrating that structural opportunities and contextual normative climates do not operate in isolation from individual characteristics. Overall, contact opportunities promoted intergroup friendship, but the degree of responsiveness depended on the individual's sense of neighbourhood belonging. Those who felt more connected and invested in their local community were particularly likely to translate the presence of diversity into meaningful intergroup friendships. Such findings suggest that while structural diversity provides the necessary context for intergroup contact, feelings of connection and investment in one's local community may provide the motivation and social confidence to reach out across group boundaries. In contrast, the negative main effect of local anti‐immigrant norms—measured by Brexit voting—was weaker among individuals higher in conscientiousness. This finding suggests that highly conscientious individuals may navigate social environments in ways that preserve stable relationships, making their intergroup friendships more resilient to shifts in local norms. Taken together, these findings highlight the value of a multilevel perspective: neither individual predispositions nor neighbourhood characteristics alone fully account for intergroup engagement; the interplay between the two is critical.

Importantly, while the main effects reported above are interpreted for ethnic majority group members, we also observed differences between minority and majority group members. Specifically, higher neighbourhood belonging was associated with more intergroup friendships for majority group members, but with fewer intergroup friendships for minority group members. These patterns echo debates in the literature on bonding vs. bridging social capital (Putnam, 2007), where strong intragroup networks can sometimes come at the expense of intergroup ties. For majority members, a strong sense of neighbourhood belonging may signal inclusion and security and encourage resilience and exploration (Stevenson et al., 2019, 2020), whereas for minorities it may reflect protective bonding within co‐ethnic networks, consistent with work showing that minority individuals sometimes cultivate strong intragroup ties as a protective strategy in response to perceived discrimination or marginalization (Branscombe et al., 1999).

At the contextual‐level, the association between local racial hate crime incidence and intergroup friendship also varied by group membership. Specifically, for majority group members, living in an area with higher objective levels of racial hate crimes predicted more intergroup friendships (even after controlling for ethnic composition of the area, population density and regional deprivation), whereas such associations were absent for minorities. Unlike our voting‐based measures of anti‐immigration norms, which reflect the prevailing attitudes and preferences of the local community, this measure captures racially motivated incidents arising from a fringe of criminal ingroup perpetrators. Our findings suggest that greater prevalence of these incidents may, somewhat paradoxically, create conditions that encourage bridge‐building: when intergroup tension escalates into violence, advantaged ethnic majority residents may engage across group lines to reduce community tensions, express solidarity, and repair relations with targeted groups (Doosje et al., 1998; Hässler et al., 2020; Mazziotta et al., 2014). Likewise, at the individual‐level a higher perceived prevalence of racially motivated incidents predicted more cross‐ethnic friendships among majority participants, reinforcing the idea that personal recognition of intergroup wrongdoings may motivate engagement rather than avoidance.

### Limitations and future directions

Despite the contributions of this study, several limitations warrant consideration and suggest directions for future research. First, the use of secondary data imposes several measurement limitations. Intergroup friendships were measured with a single proportional item, reflecting the share of individuals' friends who are from a different ethnic group. Although consistent with prior work (see Davies et al., 2011), this approach conflates increases in cross‐group ties with decreases in same‐group ties and provides no information on the total number of friendships. Collecting independent counts of cross‐group and same‐group friendships or employing full social‐network data that capture the number, duration and closeness of friendships could yield a more nuanced understanding of intergroup relationships. Similarly, many of the predictor variables, including personality traits, were measured with brief scales, resulting in relatively low internal reliabilities for some measures. Future research would benefit from more comprehensive assessments to improve methodological precision.

Future research would also benefit from richer theorization and more varied indicators of the neighbourhood context, including the normative and historical meanings attached to place. In the present research, neighbourhood belonging was positively associated with intergroup friendships among ethnic majority group members, and this association did not vary with neighbourhood‐level support for diversity. However, the effect of neighbourhood belonging may depend on how the neighbourhood is normatively and symbolically structured and regulated. For example, when place‐based neighbourhood identities are framed as inclusive, belonging may support cross‐group friendship formation. By contrast, in communities with histories of segregation or conflict or where demographic changes have been experienced as a threat, strong neighbourhood attachment may instead reinforce ingroup territoriality and reduce engagement with outgroups (Bernardo & Palma‐Oliveira, 2016; Dixon et al., 2022).

Relatedly, despite the strengths of multilevel and cross‐lagged models in reducing certain biases, unobserved confounding variables remain a concern. For instance, individuals predisposed to forming intergroup friendships may self‐select into more diverse or egalitarian neighbourhoods, potentially contributing to observed associations. Quasi‐experimental designs, such as natural experiments following local policy changes or demographic shifts, would strengthen causal inference in future research.

Finally, although this study employed a longitudinal design, the three waves of data spanned several years which may obscure shorter‐term dynamics in how individual or contextual change affect intergroup friendship formation. While our RI‐CLPMs allowed us to disentangle stable between‐person differences from within‐person fluctuations; the lack of significant within‐person cross‐lagged effects suggests that changes in individual‐level predictors such as neighbourhood belonging or perceived racial hate crime prevalence over the observed intervals did not meaningfully drive subsequent changes in intergroup friendships. This does not negate the broader relationship: the multilevel models indicate that individuals with higher overall neighbourhood belonging report more intergroup friendships – but our design is not able to capture whether shifts in belonging translate into shifts in friendships. This pattern may reflect the fact that intergroup friendships themselves are relatively stable over time and therefore less sensitive to short‐term changes in the predictors, or that key within‐person mechanisms occur over shorter time scales than our design can capture (see Dormann & Griffin, 2015). Future studies could employ higher‐frequency panel data or event‐based designs to better assess these dynamic processes.

## CONCLUSION

Using rich longitudinal data and multilevel modelling, this study provides a comprehensive examination of how individual dispositions and neighbourhood‐level factors together facilitate intergroup friendships. The findings show that intergroup friendships are the product of both opportunity and motivation, conditioned by the interaction of person and place. The impact of local diversity and social norms vary depending on residents' characteristics, and processes operate differently for majority and minority residents. Taken together, the results highlight how individual and neighbourhood factors combine to determine when cross‐group neighbours become friends.

## AUTHOR CONTRIBUTIONS

**Rose Meleady:** Conceptualization; funding acquisition; writing – original draft; methodology; validation; project administration; supervision; investigation. **Hannah K. Peetz:** Methodology; visualization; writing – review and editing; formal analysis; data curation; conceptualization; investigation; validation. **Shelley McKeown:** Conceptualization; funding acquisition; writing – review and editing; methodology; investigation; supervision. **George Leckie:** Conceptualization; funding acquisition; writing – review and editing; methodology; formal analysis; investigation; validation; supervision. **Jo Broadwood:** Conceptualization; funding acquisition; investigation.

## CONFLICT OF INTEREST STATEMENT

One of the co‐authors of this manuscript, Dr. Shelley McKeown, is the Editor‐in‐Chief of *The British Journal of Social Psychology*.

## Supporting information


Data S1.

## Data Availability

This study uses data from *Understanding Society: the UK Household Longitudinal Study*, which are available to registered users through the UK Data Service. Due to licensing restrictions, the data cannot be shared directly by the authors.

## Footnotes

^1^
While participants had yearly assessments, the total data collection period for each wave was 2 years (e.g., for some participants wave 1 was in 2009 and wave 2 in 2010 whereas for others wave 1 was in 2010 and wave 2 in 2011). Therefore, waves do not correspond to calendar years. Wave 3 was around 2011–2013, wave 6 around 2014–2016 and wave 9 around 2017–2019.

^2^
Due to missingness, the sample for analyses is substantially smaller. See the section on missingness for more information.

^3^
This sensitivity analysis was conducted using a simulation‐based approach for the main effects of the full model reported in the Results section. Specifically, the effect size of each main effect was varied one at a time from *β* = .01 to *β* = .25 in increments of .01, while all other fixed effects and the random‐effects covariance structure were held constant at their model‐estimated values. For each effect size, statistical power was estimated via 30 model‐based simulations.

^4^
For deviations from our pre‐registration, please see the Supporting Information (Appendix S3).

^5^
The separate group models are included in the Appendix S3, but we caution against comparing majority vs. minority group models as differences in significance or effect size do not necessarily mean that the differences are significant.

^6^
In the multilevel model, the association between neighbourhood belonging and intergroup friendship shifted from a small negative bivariate correlation to a small positive effect. This change likely reflects a minor suppression effect arising from shared variance with other predictors in the multivariate model, rather than a meaningful reversal in direction. No single predictor was responsible for this effect, which is a common occurrence in models with correlated predictors.

^7^
One RI‐CLPM freely estimated within‐ and between‐person paths for each group while the second RI‐CLPM constrained within‐ and between‐person paths to be equal across groups. Likelihood‐ratio tests comparing the unconstrained to the constrained model showed a significant change in fit, indicating that the unconstrained model is a better fit for the data and hence there are differences between majority and minority group members. We therefore report the results of the unconstrained model here.

^8^
The RI‐CLPM showed no significant between‐person association between neighbourhood belonging and intergroup friendships, despite the positive effect observed in the multilevel model. This difference reflects how the models partition variance and handle covariates: the RI‐CLPM separates stable, trait‐like between‐person differences from within‐person fluctuations and focuses on a smaller set of variables, whereas the multilevel model simultaneously accounts for multiple covariates and captures both stable differences and longer‐term variation across individuals. The lack of a significant between‐person association in the RI‐CLPM does not imply the absence of a broader link; rather, it suggests that the association is influenced by other predictors and is not driven solely by perfectly stable, time‐invariant differences.

## References

[bjso70068-bib-0001] Abascal, M. , & Baldassarri, D. (2015). Love thy neighbor? Ethnoracial diversity and trust reexamined. American Journal of Sociology, 121(3), 722–782. 10.1086/683144 26900618

[bjso70068-bib-0002] Al Ramiah, A. , Schmid, K. , Hewstone, M. , & Floe, C. (2014). Why are all the White (Asian) kids sitting together in the cafeteria? Resegregation and the role of intergroup attributions and norms. British Journal of Social Psychology, 54(1), 100–124. 10.1111/bjso.12064 24597949

[bjso70068-bib-0003] Allport, G. (1954). The nature of prejudice. Addison‐Wesley.

[bjso70068-bib-0004] Álvarez‐Benjumea, A. , & Winter, F. (2020). The breakdown of antiracist norms: A natural experiment on hate speech after terrorist attacks. Proceedings of the National Academy of Sciences, 117(37), 22800–22804. 10.1073/pnas.2007977117 PMC7502775 32873640

[bjso70068-bib-0005] Antonoplis, S. , & John, O. P. (2022). Who has different‐race friends, and does it depend on context? Openness (to other), but not agreeableness, predicts lower racial homophily in friendship networks. Journal of Personality and Social Psychology, 122(5), 894–919. 10.1037/pspp0000413 35404642

[bjso70068-bib-0006] Ashton, M. C. , & Lee, K. (2007). Empirical, theoretical, and practical advantages of the HEXACO model of personality structure. Personality and Social Psychology Review, 11(2), 150–166. 10.1177/1088868306294907 18453460

[bjso70068-bib-0007] Bahns, A. J. (2017). Threat as justification of prejudice. Group Processes & Intergroup Relations, 20(1), 52–74. 10.1177/1368430215591042

[bjso70068-bib-0008] Bates, D. , Mächler, M. , Bolker, B. , & Walker, S. (2015). Fitting linear mixed‐effects models using lme4. Journal of Statistical Software, 67, 1–48. 10.18637/jss.v067.i01

[bjso70068-bib-0009] Belgioioso, M. , Dworschak, C. , & Gleditsch, K. S. (2023). Local deprivation predicts right‐wing hate crime in England. PLoS One, 18(9), e0289423. 10.1371/journal.pone.0289423 37672541 PMC10482284

[bjso70068-bib-0010] Bernardo, F. , & Palma‐Oliveira, J. M. (2016). Urban neighbourhoods and intergroup relations: The importance of place identity. Journal of Environmental Psychology, 45, 239–251. 10.1016/j.jenvp.2016.01.006

[bjso70068-bib-0011] Binder, J. , Zagefka, H. , Brown, R. , Funke, F. , Kessler, T. , Mummendey, A. , Maquil, A. , Demoulin, S. , & Leyens, J.‐P. (2009). Does contact reduce prejudice or does prejudice reduce contact? A longitudinal test of the contact hypothesis among majority and minority groups in three European countries. Journal of Personality and Social Psychology, 96(4), 843–856. 10.1037/a0013470 19309206

[bjso70068-bib-0012] Boschman, S. (2018). Individual differences in the neighbourhood level determinants of residential satisfaction. Housing Studies, 33(7), 1127–1143. 10.1080/02673037.2018.1424804

[bjso70068-bib-0013] Bou Zeineddine, F. , & Leach, C. W. (2021). Feeling and thought in collective action on social issues: Toward a systems perspective. Social and Personality Psychology Compass, 15(7), e12622. 10.1111/spc3.12622

[bjso70068-bib-0014] Branscombe, N. R. , Schmitt, M. T. , & Harvey, R. D. (1999). Perceiving pervasive discrimination among African Americans: Implications for group identification and well‐being. Journal of Personality and Social Psychology, 77(1), 135–149. 10.1037/0022-3514.77.1.135

[bjso70068-bib-0015] Brown, G. G. , Reed, P. , & Harris, C. C. (2002). Testing a place‐based theory for environmental evaluation: An Alaska case study. Applied Geography, 22(1), 49–76. 10.1016/S0143-6228(01)00019-4

[bjso70068-bib-0016] Bynner, C. (2019). Intergroup relations in a super‐diverse neighbourhood: The dynamics of population composition, context and community. Urban Studies, 56(2), 335–351. 10.1177/0042098017740287

[bjso70068-bib-0017] Cantle, T. (2001). Community cohesion: Report of the independent review team – The ‘Cantle Report’. https://tedcantle.co.uk/pdf/communitycohesion%20cantlereport.pdf

[bjso70068-bib-0094] Casey, L. (2016). The Casey Review: a review into opportunity and integration (Report). Department for Communities and Local Government. https://www.gov.uk/government/publications/the‐casey‐review‐a‐review‐into‐opportunity‐and‐integration

[bjso70068-bib-0018] Ceobanu, A. M. , & Escandell, X. (2010). Comparative analyses of public attitudes toward immigrants and immigration using multinational survey data: A review of theories and research. Annual Review of Sociology, 36(1), 309–328. 10.1146/annurev.soc.012809.102651

[bjso70068-bib-0019] Christ, O. , Schmid, K. , Lolliot, S. , Swart, H. , Stolle, D. , Tausch, N. , Al‐Ramiah, A. , Wagner, U. , Vertovec, S. , & Hewstone, M. (2014). Contextual effect of positive intergroup contact on outgroup prejudice. Proceedings of the National Academy of Sciences of the United States of America, 111(11), 3996–4000. 10.1073/pnas.1320901111 24591627 PMC3964129

[bjso70068-bib-0020] Clack, B. , Dixon, J. , & Tredoux, C. (2005). Eating together apart: Patterns of segregation in a multi‐ethnic cafeteria. Journal of Community & Applied Social Psychology, 15(1), 1–16. 10.1002/casp.787

[bjso70068-bib-0021] Damen, R. E. C. , Martinović, B. , & Stark, T. H. (2021). Explaining the relationship between socio‐economic status and interethnic friendships: The mediating role of preferences, opportunities, and third parties. International Journal of Intercultural Relations, 80, 40–50. 10.1016/j.ijintrel.2020.11.005

[bjso70068-bib-0022] Danckert, B. , Dinesen, P. T. , Klemmensen, R. , Nørgaard, A. S. , Stolle, D. , & Sønderskov, K. M. (2017). With an open mind: Openness to experience moderates the effect of interethnic encounters on support for immigration. European Sociological Review, 33(5), 721–733. 10.1093/esr/jcx070

[bjso70068-bib-0023] Davies, K. , Tropp, L. R. , Aron, A. , Pettigrew, T. F. , & Wright, S. C. (2011). Cross‐group friendships and intergroup attitudes: A meta‐analytic review. Personality and Social Psychology Review, 15(4), 332–351. 10.1177/1088868311411103 21844287

[bjso70068-bib-0024] Davis, M. , Seibert, R. , & Breed, W. (1966). Interracial seating patterns on New Orleans public transit. Social Problems, 13(3), 298–306. 10.2307/799256

[bjso70068-bib-0025] Dhont, K. , & Van Hiel, A. (2009). We must not be enemies: Interracial contact and the reduction of prejudice among authoritarians. Personality and Individual Differences, 46(2), 172–177. 10.1016/j.paid.2008.09.022

[bjso70068-bib-0026] Dixon, J. , & Durrheim, K. (2003). Contact and the ecology of racial division: Some varieties of informal segregation. British Journal of Social Psychology, 42(1), 1–23. 10.1348/014466603763276090 12713753

[bjso70068-bib-0027] Dixon, J. , Sturgeon, B. , Huck, J. , Hocking, B. , Jarman, N. , Bryan, D. , Whyatt, D. , Davies, G. , & Tredoux, C. (2022). Navigating the divided city: Place identity and the time‐geography of segregation. Journal of Environmental Psychology, 84, 101908. 10.1016/j.jenvp.2022.101908

[bjso70068-bib-0028] Doosje, B. , Branscombe, N. R. , Spears, R. , & Manstead, A. S. (1998). Guilty by association: When one's group has a negative history. Journal of Personality and Social Psychology, 75(4), 872.

[bjso70068-bib-0029] Dormann, C. , & Griffin, M. A. (2015). Optimal time lags in panel studies. Psychological Methods, 20(4), 489–505. 10.1037/met0000041 26322999

[bjso70068-bib-0030] Duden, G. S. , Reiter, J. , Bauer, C. , Haslinger, C. , Mroz, M. , & Rohmann, A. (2025). “Knowing my way around” – The role of urban spaces for migrant identity content and sense of belonging in Germany. Identity, 25(1), 75–96. 10.1080/15283488.2024.2330909

[bjso70068-bib-0031] Esses, V. M. (2021). Prejudice and discrimination toward immigrants. Annual Review of Psychology, 72, 503–531. 10.1146/annurev-psych-080520-102803 32916080

[bjso70068-bib-0032] Furnham, A. , & Fenton‐O'Creevy, M. (2018). Personality and political orientation. Personality and Individual Differences, 129, 88–91. 10.1016/j.paid.2018.03.020

[bjso70068-bib-0033] Green, E. G. T. , & Staerklé, C. (2023). Migration and multiculturalism. In L. Huddy , D. O. Sears , J. S. Levy , & J. Jerit (Eds.), The Oxford handbook of political psychology (3rd ed., pp. 1016–1061). Oxford University Press. 10.1093/oxfordhb/9780197541302.013.27

[bjso70068-bib-0034] Green, E. G. T. , Visintin, E. P. , Sarrasin, O. , & Hewstone, M. (2019). When integration policies shape the impact of intergroup contact on threat perceptions: A multilevel study across 20 European countries. Journal of Ethnic and Migration Studies, 46(3), 631–648. 10.1080/1369183X.2018.1550159

[bjso70068-bib-0035] Hamaker, E. L. , Kuiper, R. M. , & Grasman, R. P. P. P. (2015). A critique of the cross‐lagged panel model. Psychological Methods, 20(1), 102–116. 10.1037/a0038889 25822208

[bjso70068-bib-0036] Harris, P. B. , Werner, C. M. , Brown, B. B. , & Ingebritsen, D. (1995). Relocation and privacy regulation: A cross‐cultural analysis. Journal of Environmental Psychology, 15(4), 311–320. 10.1006/jevp.1995.0027

[bjso70068-bib-0037] Hässler, T. , Ullrich, J. , Bernardino, M. , Shnabel, N. , Van Laar, C. , Valdenegro, D. , Sebben, S. , Tropp, L. R. , Visintin, E. P. , González, R. , Ditlmann, R. K. , Abrams, D. , Selvanathan, H. P. , Branković, M. , Wright, S. , von Zimmermann, J. , Pasek, M. , Aydin, A. L. , Žeželj, I. , … Ugarte, L. M. (2020). A large‐scale test of the link between intergroup contact and support for social change. Nature Human Behaviour, 4(4), 380–386. 10.1038/s41562-019-0815-z 31988440

[bjso70068-bib-0038] Hello, E. , Scheepers, P. , & Sleegers, P. (2006). Why the more educated are less inclined to keep ethnic distance: An empirical test of four explanations. Ethnic and Racial Studies, 29(5), 959–985. 10.1080/01419870600814015

[bjso70068-bib-0039] HomeOffice . (2024). Hate crime, England and Wales: Year ending March 2024 . https://www.gov.uk/government/statistics/hate‐crime‐england‐and‐wales‐year‐ending‐march‐2024/hate‐crime‐england‐and‐wales‐year‐ending‐march‐2024

[bjso70068-bib-0040] Jackson, J. W. , & Poulsen, J. R. (2005). Contact experiences mediate the relationship between five‐factor model personality traits and ethnic prejudice. Journal of Applied Social Psychology, 35(4), 667–685. 10.1111/j.1559-1816.2005.tb02140.x

[bjso70068-bib-0041] John, O. P. , & Srivastava, S. (1999). The big five trait taxonomy: History, measurement, and theoretical perspectives. In L. A. Pervin & O. P. John (Eds.), Handbook of personality: Theory and research (2nd ed., pp. 102–138). Guilford Press.

[bjso70068-bib-0042] Johnston, C. D. , Newman, B. J. , & Velez, Y. R. (2015). Ethnic change, personality, and polarization over immigration in the American public. Public Opinion Quarterly, 79(3), 662–686. 10.1093/poq/nfv022

[bjso70068-bib-0043] Kauff, M. , Beneda, M. , Paolini, S. , Bilewicz, M. , Kotzur, P. , O'Donnell, A. W. , Stevenson, C. , Wagner, U. , & Christ, O. (2020). How do we get people into contact? Predictors of intergroup contact and drivers of contact seeking. Journal of Social Issues, 77(1), 38–63. 10.1111/josi.12398

[bjso70068-bib-0044] Kaufmann, E. J. (2017). Levels or changes?: Ethnic context, immigration and the UK Independence party vote. Electoral Studies, 48, 57–69. 10.1016/j.electstud.2017.05.002

[bjso70068-bib-0045] Kaufmann, E. J. , & Harris, G. (2015). “White flight” or positive contact? Local diversity and attitudes to immigration in Britain. Comparative Political Studies, 48(12), 1563–1590. 10.1177/0010414015581684

[bjso70068-bib-0046] Kotzur, P. F. , & Wagner, U. (2021). The dynamic relationship between contact opportunities, positive and negative intergroup contact, and prejudice: A longitudinal investigation. Journal of Personality and Social Psychology, 120(2), 418–442. 10.1037/pspi0000258 32700961

[bjso70068-bib-0047] Kros, M. , & Hewstone, M. (2020). Negative and positive interethnic contact and the association of ethnic neighbourhood composition with trust, cohesion, and prejudice. European Sociological Review, 36(6), 937–956. 10.1093/esr/jcaa032

[bjso70068-bib-0048] Laurence, J. , Schmid, K. , & Hewstone, M. (2018). Ethnic diversity, inter‐group attitudes and countervailing pathways of positive and negative inter‐group contact: An analysis across workplaces and neighborhoods. Social Indicators Research, 136(2), 719–749. 10.1007/s11205-017-1570-z 29563660 PMC5842268

[bjso70068-bib-0049] Letki, N. (2008). Does diversity erode social cohesion? Social capital and race in British neighbourhoods. Political Studies, 56(1), 99–126. 10.1111/j.1467-9248.2007.00692.x

[bjso70068-bib-0050] Lynn, P. (2009). Sample design for Understanding Society. *Understanding Society Working Paper Series, 2009*.

[bjso70068-bib-0051] Mazziotta, A. , Feuchte, F. , Gausel, N. , & Nadler, A. (2014). Does remembering past ingroup harmdoing promote postwar cross‐group contact? Insights from a field‐experiment in Liberia. European Journal of Social Psychology, 44(1), 43–52. 10.1002/ejsp.1986

[bjso70068-bib-0052] McKeown, S. , & Dixon, J. (2017). The “contact hypothesis”: Critical reflections and future directions. Social and Personality Psychology Compass, 11(1), e12295. 10.1111/spc3.12295

[bjso70068-bib-0053] McKeown, S. , & Taylor, L. K. (2018). Perceived peer and school norm effects on youth antisocial and prosocial behaviours through intergroup contact in Northern Ireland. British Journal of Social Psychology, 57(3), 652–665. 10.1111/bjso.12257 29663432

[bjso70068-bib-0054] Meleady, R. (2021). “Nudging” intergroup contact: Normative social influences on intergroup contact engagement. Group Processes & Intergroup Relations, 24(7), 1180–1199. 10.1177/13684302211016047

[bjso70068-bib-0055] Meleady, R. , Seger, C. R. , & Vermue, M. (2017). Examining the role of positive and negative intergroup contact and anti‐immigrant prejudice in B rexit. British Journal of Social Psychology, 56(4), 799–808. 28639419 10.1111/bjso.12203

[bjso70068-bib-0056] Mesch, G. S. , & Manor, O. (1998). Social ties, environmental perception, and local attachment. Environment and Behavior, 30(4), 504–519. 10.1177/001391659803000405

[bjso70068-bib-0057] Miller, D. T. , & Laurin, K. (2025). History of social psychology: Four enduring tensions. In D. T. Gilbert , S. T. Fiske , E. J. Finkel , & W. B. Mendes (Eds.), The handbook of social psychology (6th ed.). Situational Press. 10.70400/DCSX1997

[bjso70068-bib-0058] National Centre for Social Research . (2025). People in the UK prefer a tougher approach on immigration compared with the US, but Americans more conservative on social issues . https://natcen.ac.uk/news/people‐uk‐prefer‐tougher‐approach‐immigration‐compared‐us‐americans‐more‐conservative‐social

[bjso70068-bib-0059] Office for National Statistics . (2022a). International migration, England and Wales: Census 2021 . https://www.ons.gov.uk/peoplepopulationandcommunity/populationandmigration/internationalmigration/bulletins/internationalmigrationenglandandwales/census2021

[bjso70068-bib-0060] Office for National Statistics . (2022b). *Population of England and Wales* (Ethnicity facts and figures). https://www.ethnicity‐facts‐figures.service.gov.uk/uk‐population‐by‐ethnicity/national‐and‐regional‐populations/population‐of‐england‐and‐wales/latest/

[bjso70068-bib-0061] Orr, R. , McKeown, S. , Cairns, E. , & Stringer, M. (2012). Examining non‐racial segregation: A micro‐ecological approach. British Journal of Social Psychology, 51(4), 717–723. 10.1111/j.2044-8309.2011.02080.x 22118404

[bjso70068-bib-0062] Paolini, S. , Harwood, J. , Hewstone, M. , & Neumann, D. L. (2018). Seeking and avoiding intergroup contact: Future frontiers of research on building social integration. Social and Personality Psychology Compass, 12(12), e12422. 10.1111/spc3.12422

[bjso70068-bib-0063] Paolini, S. , Wright, S. C. , Dys‐Steenbergen, O. , & Favara, I. (2016). Self‐expansion and intergroup contact: Expectancies and motives to self‐expand lead to greater interest in outgroup contact and more positive intergroup relations. Journal of Social Issues, 72(3), 450–471. 10.1111/josi.12176

[bjso70068-bib-0064] Pettigrew, T. F. (1997). Generalized intergroup contact effects on prejudice. Personality and Social Psychology Bulletin, 23(2), 173–185. 10.1177/0146167297232006

[bjso70068-bib-0065] Pettigrew, T. F. (2018). The emergence of contextual social psychology. Personality and Social Psychology Bulletin, 44(7), 963–971. 10.1177/0146167218756033 29528782

[bjso70068-bib-0066] Pettigrew, T. F. , & Tropp, L. R. (2006). A meta‐analytic test of intergroup contact theory. Journal of Personality and Social Psychology, 90(5), 751–783. 10.1037/0022-3514.90.5.751 16737372

[bjso70068-bib-0067] Prati, F. , Schaefer, S. J. , Hewstone, M. , & Christ, O. (2022). Antecedents of positive and negative intergroup contact: Evidence from a diary study. International Journal of Psychology, 57(4), 524–534. 10.1002/ijop.12841 35263456 PMC10286651

[bjso70068-bib-0068] Putnam, R. D. (2007). E pluribus unum: Diversity and community in the twenty‐first century. The 2006 Johan Skytte prize lecture. Scandinavian Political Studies, 30(2), 137–174. 10.1111/j.1467-9477.2007.00176.x

[bjso70068-bib-0069] R Core Team . (2024). *R: A language and environment for statistical computing* [Computer software]. https://www.R‐project.org/

[bjso70068-bib-0070] Ron, Y. , Solomon, J. , Halperin, E. , & Saguy, T. (2017). Willingness to engage in intergroup contact: A multilevel approach. Peace and Conflict: Journal of Peace Psychology, 23(3), 210–218. 10.1037/pac0000204

[bjso70068-bib-0071] Rosseel, Y. (2012). Lavaan: An r package for structural equation modeling. Journal of Statistical Software, 48, 1–36. 10.18637/jss.v048.i02

[bjso70068-bib-0072] Silva, L. , Bonomi Bezzo, F. , Laurence, J. , & Schmid, K. (2023). Effects of absolute levels of neighbourhood ethnic diversity vs. changes in neighbourhood diversity on prejudice: Moderation by individual differences in personality. Social Science Research, 115, 102919. 10.1016/j.ssresearch.2023.102919 37858365

[bjso70068-bib-0073] Stevenson, C. , Costa, S. , Easterbrook, M. J. , McNamara, N. , & Kellezi, B. (2020). Social cure processes help lower intergroup anxiety among neighborhood residents. Political Psychology, 41(6), 1093–1111. 10.1111/pops.12667

[bjso70068-bib-0074] Stevenson, C. , Easterbrook, M. , Harkin, L. , McNamara, N. , Kellezi, B. , & Shuttleworth, I. (2019). Neighborhood identity helps residents cope with residential diversification: Contact in increasingly mixed neighborhoods of Northern Ireland. Political Psychology, 40(2), 277–295. 10.1111/pops.12510

[bjso70068-bib-0075] Stevenson, C. , Turner, R. , & Costa, S. (2021). “Welcome to our neighbourhood”: Collective confidence in contact facilitates successful mixing in residential settings. Group Processes & Intergroup Relations, 24(8), 1448–1466. 10.1177/1368430220961151

[bjso70068-bib-0076] Stürmer, S. , Benbow, A. E. F. , Siem, B. , Barth, M. , Bodansky, A. N. , & Lotz‐Schmitt, K. (2013). Psychological foundations of xenophilia: The role of major personality traits in predicting favorable attitudes toward cross‐cultural contact and exploration. Journal of Personality and Social Psychology, 105(5), 832–851. 10.1037/a0033488 23834640

[bjso70068-bib-0077] Swart, H. , Hewstone, M. , Christ, O. , & Voci, A. (2011). Affective mediators of intergroup contact: A three‐wave longitudinal study in South Africa. Journal of Personality and Social Psychology, 101(6), 1221–1238. 10.1037/a0024450 21728450

[bjso70068-bib-0078] Tartaglia, S. (2013). Different predictors of quality of life in urban environments. Social Indicators Research, 113(3), 1045–1053. 10.1007/s11205-012-0126-5

[bjso70068-bib-0079] The Challenge . (2019). British Integration Survey . The Challenge. https://www.belongnetwork.co.uk/resources/british‐integration‐survey‐2019/

[bjso70068-bib-0080] Titzmann, P. F. , Brenick, A. , & Silbereisen, R. K. (2015). Friendships fighting prejudice: A longitudinal perspective on adolescents' cross‐group friendships with immigrants. Journal of Youth and Adolescence, 44(6), 1318–1331. 10.1007/s10964-015-0256-6 25647141

[bjso70068-bib-0081] Tredoux, C. G. , & Dixon, J. A. (2009). Mapping the multiple contexts of racial isolation: The case of long street, Cape Town. Urban Studies, 46(4), 761–777. 10.1177/0042098009102128

[bjso70068-bib-0082] Tropp, L. R. , & Bianchi, R. A. (2006). Valuing diversity and interest in intergroup contact. Journal of Social Issues, 62(3), 533–551. 10.1111/j.1540-4560.2006.00472.x

[bjso70068-bib-0085] Tropp, L. R. , & Pettigrew, T. F. (2005). Relationships between intergroup contact and prejudice among minority and majority status groups. Psychological Science, 16(12), 951–957. 10.1111/j.1467-9280.2005.01643.x 16313659

[bjso70068-bib-0084] Tropp, L. R. , O'Brien, T. C. , & Migacheva, K. (2014). How peer norms of inclusion and exclusion predict children's interest in cross‐ethnic friendships. Journal of Social Issues, 70(1), 151–166. 10.1111/josi.12052

[bjso70068-bib-0083] Tropp, L. R. , O'Brien, T. C. , González Gutierrez, R. , Valdenegro, D. , Migacheva, K. , de Tezanos‐Pinto, P. , Berger, C. , & Cayul, O. (2016). How school norms, peer norms, and discrimination predict interethnic experiences among ethnic minority and majority youth. Child Development, 87(5), 1436–1451. 10.1111/cdev.12608 27684397

[bjso70068-bib-0086] Turner, R. N. , Dhont, K. , Hewstone, M. , Prestwich, A. , & Vonofakou, C. (2014). The role of personality factors in the reduction of intergroup anxiety and amelioration of outgroup attitudes via intergroup contact. European Journal of Personality, 28(2), 180–192. 10.1002/per.1927

[bjso70068-bib-0087] University of Essex, Institute for Social and Economic Research . (2024). *Understanding Society: Waves 1–14, 2009–2023 and Harmonised BHPS: Waves 1–18, 1991–2009: Vol. 19th Edition* [Data set].

[bjso70068-bib-0088] Van Assche, J. , Roets, A. , Dhont, K. , & Van Hiel, A. (2014). Diversity and out‐group attitudes in The Netherlands: The role of authoritarianism and social threat in the neighbourhood. Journal of Ethnic and Migration Studies, 40(9), 1414–1430. 10.1080/1369183X.2013.876895

[bjso70068-bib-0089] van de Vyver, J. , Houston, D. M. , Abrams, D. , & Vasiljevic, M. (2016). Boosting belligerence: How the July 7, 2005, London bombings affected liberals' moral foundations and prejudice. Psychological Science, 27(2), 169–177. 10.1177/0956797615615584 26674127 PMC4750069

[bjso70068-bib-0090] van Tubergen, F. (2015). Ethnic boundaries in Core discussion networks: A multilevel social network study of Turks and Moroccans in The Netherlands. Journal of Ethnic and Migration Studies, 41(1), 101–116. 10.1080/1369183x.2014.886955

[bjso70068-bib-0091] Vezzali, L. , Turner, R. , Capozza, D. , & Trifiletti, E. (2018). Does intergroup contact affect personality? A longitudinal study on the bidirectional relationship between intergroup contact and personality traits. European Journal of Social Psychology, 48(2), 159–173. 10.1002/ejsp.2313

[bjso70068-bib-0092] Wagner, U. , Christ, O. , Pettigrew, T. F. , Stellmacher, J. , & Wolf, C. (2006). Prejudice and minority proportion: Contact instead of threat effects. Social Psychology Quarterly, 69(4), 380–390. 10.1177/019027250606900406

[bjso70068-bib-0093] Wickes, R. , Zahnow, R. , White, G. , & Mazerolle, L. (2013). Ethnic diversity and its impact on community social cohesion and neighborly exchange. Journal of Urban Affairs, 36(1), 51–78. 10.1111/juaf.12015

