# Supplementary material for: A longitudinal multilevel analysis of individual‐ and contextual‐level predictors of cross‐ethnic friendships in the UK
Source: Br J Soc Psychol. 2026 Mar 17;65(2):e70068. doi: 10.1111/bjso.70068 (PMC12996426; doi:10.1111/bjso.70068)
Supplement: Supplementary file 1 — Data S1. [file BJSO-65-0-s001.docx]

**Overview of Online Supplementary Materials**

**Appendix S1 – Intercept only models**

**Appendix S1 Table S1 -** *ICC for the different geographical clustering variables*

**Appendix S2 – Measurement invariance, assumptions, and cumulative link mixed model**

**Appendix S2 Table S2 -** *Fit measures and change in fit measures for configural, metric, scalar and strict measurement invariance for neighbourhood belonging across time*

**Appendix S2 Table S3 -** *Full Results Table of Cumulative Link Mixed Model (CLMM)*

**Appendix S2 Figure S1 -** *Scatterplot of residuals vs fitted values of the main analysis*

**Appendix S3 – Partial multilevel models and separate group models**

**Appendix S3 Table S4 -** *Results main effects only model*

**Appendix S3 Table S5 -** *Results cross-level interaction model*

**Appendix S3 Table S6 -** *Results for Majority (White) participants*

**Appendix S3 Table S7 -** *Results for Asian participants*

**Appendix S3 Table S8 -** *Results for Black participants*

**Appendix S3 Table S9 -** *Results for Minority participants*

**Appendix S3 Table S10 -** *Results of fully-interacted three-group model*

**Appendix S4 – Full results for main models**

**Appendix S4 Table S11 -** *Full Results Table of Full Multilevel Model including Control Variables*

**Appendix S4 Figure S2 -** *Two-way interactions between group membership and control variables*

**Appendix S4 Figure S3 -** *Three-way interactions between group, local egalitarian policy support in the form of Brexit remain votes, and individual-level predictors*

**Appendix S4 Figure S4 -** *Three-way interactions between group, local egalitarian policy support in the form of absence of right-wing support, and individual-level predictors*

**Appendix S5 – Correlations**

**Appendix S5 Table S12 -** *Full Pearson correlation table T1*

**Appendix S5 Table S13 -** *Full Pearson correlation table T2*

**Appendix S5 Table S14 -** *Full Pearson correlation table T3*

**Appendix S5 Table S15 -** *Pearson correlation of time-varying variables between timepoints*

**References**

**Appendix S1**

**Intercept only models**

We ran several intercept-only models to examine the variance explained by each of the different geographical levels. Each intercept-only model included a random intercept for the participant level. In addition, we examined several different combinations of random intercepts for the different geographical levels. Table 1S gives an overview of the ICCs of the random intercepts in the different models. The most complete model including all nesting variables did not converge. Likelihood-ratio tests indicated that a model with two geographical levels fitted significantly better than a model with only one geographical level. Given the similar variance explained, we decided to select the model with LSOA and LAD as our baseline model. This way we account for the smallest as well as largest level of geographical clustering.

**Table S1**

*ICC for the different geographical clustering variables*

| Model | Random intercepts | ICC Participant | ICC LSOA | ICC MSOA | ICC LAD |
| --- | --- | --- | --- | --- | --- |
| 1.1 | LSOA | 0.26 | 0.19 | - | - |
| 1.2 | MSOA | 0.28 | - | 0.16 | - |
| 1.3 | LAD | 0.33 | - | - | 0.10 |
| 2.1 | LSOA + MSOA | 0.25 | 0.05 | 0.14 | - |
| 2.2 | LSOA + LAD | 0.26 | 0.08 | - | 0.10 |
| 2.3 | MSOA + LAD | 0.29 | - | 0.05 | 0.10 |
| 3 | LSOA + MSOA + LAD | Failed to converge | | | |

**Appendix S2**

**Measurement invariance**

Most of our variables used single item measures. The exception are the personality traits and neighbourhood belonging. Since neighbourhood belonging was a time-varying variable, assessed at all three timepoints, we examined measurement invariance across time to make sure that changes neighbourhood belonging across time weren’t due to measurement non-invariance. This was not possible for the personality traits as each has only been assessed at T1. Since our sample size is quite substantial, we examined ΔCFI and ΔRMSEA between the configural, metric, scalar, and strict invariance model. Instead of χ^2^ a change of < .01 in CFI and < .015 in RMSEA is indicative of invariance (Chen, 2007; Meade et al., 2008). As can be seen in Table S5, change in fit indices was minimal and below the cut-off criteria, thus indicating that measurement invariance across time was met.

**Table S2**

*Fit measures and change in fit measures for configural, metric, scalar and strict measurement invariance for neighbourhood belonging across time*

|  | CFI | ΔCFI | RMSEA | ΔRMSEA |
| --- | --- | --- | --- | --- |
| Configural | 0.910 |  | 0.069 |  |
| Metric | 0.909 | .001 | 0.067 | .002 |
| Scalar | 0.907 | .002 | 0.066 | .001 |
| Strict | 0.906 | .002 | 0.065 | .001 |

**Assumptions**

We checked the assumptions of our final multilevel effects model. Specifically, we visually inspected the residuals and random intercepts of the geographical levels for linearity and normality. The residuals and random intercepts approximated a normal distribution. With regards to linearity, the scatterplot of the residuals showed a striped pattern (see Figure S1), reflecting the ordinal nature of the outcome variable. We therefore re-ran our main model using a cumulative link mixed model (CLMM), treating our outcome intergroup friendships as an ordinal variable. Results can be seen in Table S8. Results were generally robust between the two models. Using the same *α* < .01 cutoff, there are three noteworthy differences. Firstly, the main effect of agreeableness, which was significant in the multilevel model, was not significant in the CLMM (*β* = .044, *p* = .033). Furthermore, a significant two-way interaction between openness and neighbourhood contact opportunities emerged in the CLMM (*β* = -.137, *p* = .001). Lastly, the significant three-way interaction between openness, anti-immigrant norms in the form of Brexit leave votes and group membership was no longer significant in the CLMM (*β* = -.145, *p* = .052).

We probed the simple slopes of the two- and three-way interactions for this model as well using the slopes at -2*SD, M,* and 2*SD* as low, average, and high respectively. Note that the simple slopes in a CLMM are in log-odds units. For the interaction between openness and contact opportunities, simple slopes indicated that all three slopes were significantly different from 0 (low *β* = .866, 95% CI [.698, 1.034]; average *β* = .711, 95% CI [.630, .793]; high *β* = .557, 95% CI [.408, .706]). For the interaction between neighbourhood belonging and contact opportunities, all three slopes were significantly different from 0 as well (low *β* = .484, 95% CI [.357, .611]; average *β* = .705, 95% CI [.624, .786]; high *β* = .927, 95% CI [.786, 1.067]). Finally, for the interaction between conscientiousness and anti-immigrant norms in the form of Brexit leave votes, only the slopes at low (*β* = -.287, 95% CI [-.462, -.113]) to average (*β* = -.194, 95% CI [-.301, -.086]) but not high (*β* = -.100, 95% CI [-.281, .081]) levels of conscientiousness were significant. Thus, the cross-level interactions in the CLMM are very similar to the cross-level interactions in the multilevel model.

The two-way interactions regarding group membership also show the same pattern as in the multilevel model. Neighbourhood belonging was positively related to intergroup friendships for majority group members (*β* = .083, 95% CI [.048, .117]) but negatively related to intergroup friendships for minority group members (*β* = -.326, 95% CI [-.538, -.115]). Furthermore, racial hate crime incidence was positively related to intergroup friendships for majority group members (*β* = .121, 95% CI [.055, .187]) but unrelated for minority group members (*β* = -.052, 95% CI [-.148, .045]).

Finally, we also explored the simple slopes for the three-way interactions. The effect of anti-immigrant norms in the form of Brexit leave votes depended on neuroticism and neighbourhood belonging, which differed as a function of group membership. For majority group members, the slopes of low (*β* = -.281, 95% CI [-.419, -.143]) to average (*β* = -.166, 95% CI [-.251, -.082]) but not high (*β* = -.052, 95% CI [-.185, .082]) levels of neuroticism were significantly different from 0. For minority group participants the pattern was reversed, with the slopes of average (*β* = -.217, 95% CI [-.393, -.041]) to high (*β* = -.557, 95% CI [-.888, -.226]) but not low (*β* = .122, 95% CI [-.181, .425]) levels of neuroticism being significantly different from 0. The same pattern emerged for neighbourhood belonging. For majority group members, the slopes of low (*β* = -.238, 95% CI [-.361, -.115]) to average (*β* = -.164, 95% CI [-.248, -.080]) but not high (*β* = -.090, 95% CI [-.223, .042]) levels of neighbourhood belonging were significantly different from 0. For minority group participants the pattern was reversed, with the slopes of average (*β* = -.225, 95% CI [-.401, -.048]) to high (*β* = -.473, 95% CI [-.766, -.180]) but not low (*β* = .024, 95% CI [-.238, .285]) levels of neighbourhood belonging being significantly different from 0.

The effect of anti-immigrant norms in the form of right-wing votes depended on neuroticism and political orientation, which differed as a function of group membership. Neuroticism did not impact the effect of right-wing votes for majority group members (low *β* = .107, 95% CI [-.014, .228]; average *β* = .019, 95% CI [-.048, .085]; high *β* = -.070, 95% CI [-.187, .047]). For minority group members however, the slopes for low (*β* = -.533, 95% CI [-.798, -.269]) but not average (*β* = -.138, 95% CI [-.284, .008]) or high (*β* = .257, 95% CI [-.022, .537]) levels of neuroticism were significantly different from 0. Finally, political orientation did not impact the effect of right-wing votes for majority group members (low *β* = -.063, 95% CI [-.183, .056]; average *β* = .016, 95% CI [-.050, .083]; high *β* = .096, 95% CI [-.023, .215]). For minority group members, the slopes for high (*β* = -.395, 95% CI [-.714, -.076]) but not average (*β* = -.130, 95% CI [-.274, .015]) or low (*β* = .136, 95% CI [-.091, .362]) levels of political orientation were significantly different from 0. These results differ slightly from the multilevel model, indicating that only more extreme values of neuroticism or political orientation shape the effect of absence of right-wing support on intergroup friendships for minority group members.

**Figure S1**

*Scatterplot of residuals vs fitted values of the main analysis*

*
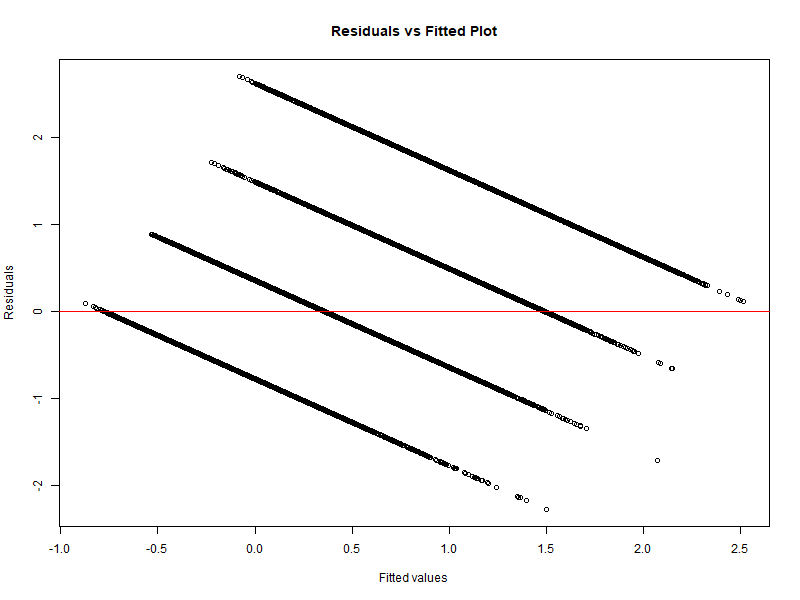
*

**Table S3**

*Full Results Table of Cumulative Link Mixed Model (CLMM)*

|  |  | | *estimate* | *se* | *z* | *p* |
| --- | --- | --- | --- | --- | --- | --- |
|  |  | *Time* | .033 | 0.016 | 2.056 | .040 |
|  |  | *Group Membership (ref. Majority)* | .173 | 0.205 | 0.844 | .399 |
| *Control* | | |  |  |  |  |
|  |  | Age | -.453 | 0.021 | -21.678 | < .001 |
|  |  | Gender (ref. male) | -.115 | 0.036 | -3.228 | .001 |
|  |  | Income | .015 | 0.014 | 1.083 | .279 |
|  |  | Education Post Secondary (ref. higher education) | -.167 | 0.050 | -3.349 | .001 |
|  |  | Education Secondary (ref. higher education) | -.380 | 0.037 | -10.212 | < .001 |
|  |  | Deprivation | -.036 | 0.025 | -1.461 | .144 |
|  |  | Population Density | .106 | 0.027 | 3.909 | < .001 |
| *Individual-Level* | | |  |  |  |  |
|  |  | Extraversion | .017 | 0.021 | 0.811 | .417 |
|  |  | Agreeableness | .044 | 0.021 | 2.133 | .033 |
|  |  | Openness | .177 | 0.021 | 8.279 | < .001 |
|  |  | Neuroticism | -.050 | 0.021 | -2.415 | .016 |
|  |  | Conscientiousness | .004 | 0.022 | 0.187 | .852 |
|  |  | Preference Conservative Party | -.109 | 0.020 | -5.476 | < .001 |
|  |  | Neighbourhood Belonging | .074 | 0.018 | 4.250 | < .001 |
|  |  | Perceived Racial Hate Crime Incidence | .074 | 0.017 | 4.466 | < .001 |
| *Contextual-Level* | | |  |  |  |  |
|  |  | Contact Opportunities | .840 | 0.055 | 15.388 | < .001 |
|  |  | Actual Racial Hate Crime Incidence | .124 | 0.034 | 3.598 | < .001 |
|  |  | Right-Wing Votes | .022 | 0.034 | 0.648 | .517 |
|  |  | Brexit LeaveVotes | -.163 | 0.043 | -3.769 | < .001 |
| *Cross-level interactions* | | |  |  |  |  |
|  | *Contact opportunities* | |  |  |  |  |
|  |  | x Extraversion | -.075 | 0.042 | -1.759 | .079 |
|  |  | x Agreeableness | .070 | 0.041 | 1.710 | .087 |
|  |  | x Openness | -.137 | 0.043 | -3.212 | .001 |
|  |  | x Neuroticism | -.035 | 0.041 | -0.856 | .392 |
|  |  | x Conscientiousness | .102 | 0.044 | 2.329 | .020 |
|  |  | x Preference Conservative Party | .086 | 0.039 | 2.197 | .028 |
|  |  | x Neighbourhood Belonging | .122 | 0.035 | 3.533 | < .001 |
|  |  | x Perceived Racial Hate Crime Incidence | -.058 | 0.028 | -2.102 | .036 |
|  | Actual Racial Hate Crime Incidence | |  |  |  |  |
|  |  | x Extraversion | .018 | 0.025 | 0.717 | .474 |
|  |  | x Agreeableness | -.027 | 0.025 | -1.046 | .295 |
|  |  | x Openness | -.008 | 0.026 | -0.305 | .761 |
|  |  | x Neuroticism | .023 | 0.025 | 0.926 | .355 |
|  |  | x Conscientiousness | -.013 | 0.026 | -0.517 | .605 |
|  |  | x Preference Conservative Party | -.021 | 0.023 | -0.893 | .372 |
|  |  | x Neighbourhood Belonging | .032 | 0.021 | 1.529 | .126 |
|  |  | x Perceived Racial Hate Crime Incidence | .016 | 0.019 | 0.884 | .377 |
|  | Right-Wing Votes | |  |  |  |  |
|  |  | x Extraversion | -.017 | 0.026 | -0.645 | .519 |
|  |  | x Agreeableness | -.005 | 0.026 | -0.183 | .855 |
|  |  | x Openness | .033 | 0.026 | 1.282 | .200 |
|  |  | x Neuroticism | -.044 | 0.025 | -1.747 | .081 |
|  |  | x Conscientiousness | -.047 | 0.027 | -1.726 | .084 |
|  |  | x Preference Conservative Party | .040 | 0.025 | 1.581 | .114 |
|  |  | x Neighbourhood Belonging | -.010 | 0.022 | -0.468 | .640 |
|  |  | x Perceived Racial Hate Crime Incidence | .012 | 0.020 | 0.575 | .565 |
|  | *Brexit Leave Votes* | |  |  |  |  |
|  |  | x Extraversion | -.034 | 0.028 | -1.229 | .219 |
|  |  | x Agreeableness | -.031 | 0.029 | -1.084 | .278 |
|  |  | x Openness | .021 | 0.029 | 0.713 | .476 |
|  |  | x Neuroticism | .057 | 0.027 | 2.087 | .037 |
|  |  | x Conscientiousness | .101 | 0.030 | 3.361 | .001 |
|  |  | x Preference Conservative Party | -.004 | 0.027 | -0.138 | .890 |
|  |  | x Neighbourhood Belonging | .037 | 0.024 | 1.490 | .136 |
|  |  | x Perceived Racial Hate Crime Incidence | -.004 | 0.023 | -0.160 | .873 |
| *Two-way Group membership interactions* | | |  |  |  |  |
|  | *Group (ref. Majority) x individual-level* | |  |  |  |  |
|  |  | x Extraversion | .147 | 0.139 | 1.052 | .293 |
|  |  | x Agreeableness | .075 | 0.131 | 0.568 | .570 |
|  |  | x Openness | -.034 | 0.143 | -0.239 | .811 |
|  |  | x Neuroticism | .032 | 0.130 | 0.246 | .806 |
|  |  | x Conscientiousness | .031 | 0.128 | 0.241 | .810 |
|  |  | x Preference Conservative Party | .121 | 0.128 | 0.943 | .346 |
|  |  | x Neighbourhood Belonging | -.386 | 0.110 | -3.520 | < .001 |
|  |  | x Perceived Racial Hate Crime Incidence | -.053 | 0.078 | -0.681 | .496 |
|  | *Group (ref. Majority) x contextual-level* | |  |  |  |  |
|  |  | x Contact Opportunities | -.213 | 0.086 | -2.489 | .013 |
|  |  | x Actual Racial Hate Crime Incidence | -.181 | 0.052 | -3.488 | < .001 |
|  |  | x Right-Wing Votes | -.171 | 0.076 | -2.268 | .023 |
|  |  | x Brexit Leave Votes | -.038 | 0.089 | -0.435 | .664 |
|  | *Group (ref. Majority) x control variables* | |  |  |  |  |
|  |  | x Age | .116 | 0.066 | 1.770 | .077 |
|  |  | x Gender | .097 | 0.095 | 1.014 | .311 |
|  |  | x Income | .232 | 0.087 | 2.664 | .008 |
|  |  | x Education 2 | .332 | 0.125 | 2.658 | .008 |
|  |  | x Education 3 | .208 | 0.110 | 1.893 | .058 |
|  |  | x Deprivation | .177 | 0.078 | 2.284 | .022 |
|  |  | x Population Density | -.032 | 0.049 | -0.648 | .517 |
|  |  | x Time | .147 | 0.139 | 1.052 | .293 |
| *Three-way group membership interactions* | | |  |  |  |  |
|  | *Contact opportunities x group (ref. Majority)* | |  |  |  |  |
|  |  | x Extraversion | .087 | 0.068 | 1.280 | .201 |
|  |  | x Agreeableness | -.136 | 0.065 | -2.098 | .036 |
|  |  | x Openness | .120 | 0.070 | 1.724 | .085 |
|  |  | x Neuroticism | .104 | 0.065 | 1.597 | .110 |
|  |  | x Conscientiousness | -.087 | 0.066 | -1.311 | .190 |
|  |  | x Preference Conservative Party | -.078 | 0.064 | -1.211 | .226 |
|  |  | x Neighbourhood Belonging | -.022 | 0.054 | -0.400 | .689 |
|  |  | x Perceived Racial Hate Crime Incidence | .053 | 0.041 | 1.292 | .196 |
|  | Actual Racial Hate Crime Incidence *x group (ref. Majority)* | |  |  |  |  |
|  |  | x Extraversion | -.028 | 0.042 | -0.656 | .512 |
|  |  | x Agreeableness | .058 | 0.044 | 1.331 | .183 |
|  |  | x Openness | -.003 | 0.045 | -0.062 | .951 |
|  |  | x Neuroticism | -.045 | 0.044 | -1.021 | .307 |
|  |  | x Conscientiousness | .000 | 0.045 | -0.006 | .995 |
|  |  | x Preference Conservative Party | .039 | 0.041 | 0.940 | .347 |
|  |  | x Neighbourhood Belonging | -.061 | 0.037 | -1.652 | .099 |
|  |  | x Perceived Racial Hate Crime Incidence | .001 | 0.029 | 0.043 | .966 |
|  | Right-Wing Votes *x group (ref. Majority)* | |  |  |  |  |
|  |  | x Extraversion | .016 | 0.057 | 0.271 | .786 |
|  |  | x Agreeableness | .010 | 0.062 | 0.167 | .868 |
|  |  | x Openness | .004 | 0.065 | 0.060 | .952 |
|  |  | x Neuroticism | .240 | 0.064 | 3.773 | < .001 |
|  |  | x Conscientiousness | .029 | 0.062 | 0.465 | .642 |
|  |  | x Preference Conservative Party | 173 | 0.065 | -2.649 | .008 |
|  |  | x Neighbourhood Belonging | 018 | 0.051 | -0.362 | .717 |
|  |  | x Perceived Racial Hate Crime Incidence | .009 | 0.037 | 0.257 | .797 |
|  | *Brexit Leave Votes x group (ref. Majority)* | |  |  |  |  |
|  |  | x Extraversion | -.030 | 0.067 | -0.446 | .655 |
|  |  | x Agreeableness | .131 | 0.070 | 1.861 | .063 |
|  |  | x Openness | -.145 | 0.074 | -1.945 | .052 |
|  |  | x Neuroticism | -.228 | 0.073 | -3.134 | .002 |
|  |  | x Conscientiousness | -.103 | 0.073 | -1.426 | .154 |
|  |  | x Preference Conservative Party | .063 | 0.071 | 0.880 | .379 |
|  |  | x Neighbourhood Belonging | -.160 | 0.060 | -2.676 | .007 |
|  |  | x Perceived Racial Hate Crime Incidence | -.005 | 0.044 | -0.111 | .911 |

*Note.* n individual-level = 18806; n LSOA level = 11181; n LAD level = 320

**Appendix S3**

**Stepwise approach**

We pre-registered a stepwise approach, in which we built up to a full model as reported in the main text. We made one deviation to the pre-registration: We decided to combine the Asian and Black participant groups into one minority group in order to simplify interpretation and facilitate clear comparisons between majority and minority group experiences. Here we report on all steps as pre-registered, including the full three-group model.

***Main effects only model***

We started with a main-effects only model. This model was conducted on the full sample, not accounting for group membership. Effects are thus for the entire sample and not specific groups.

**Table S4**

*Results main effects only model*

|  |  |  | *β* | *se* | *t* | *p* |
| --- | --- | --- | --- | --- | --- | --- |
|  |  | *Intercept* | .055 | 0.016 | 3.485 | .001 |
|  |  | *Time* | -.109 | 0.007 | -16.395 | < .001 |
| *Control* | | |  |  |  |  |
|  |  | Age | -.109 | 0.007 | -16.395 | < .001 |
|  |  | Gender (ref. male) | -.027 | 0.011 | -2.379 | .017 |
|  |  | Income | .007 | 0.005 | 1.378 | .168 |
|  |  | Education Post Secondary (ref. higher education) | .057 | 0.017 | 3.394 | .001 |
|  |  | Education Secondary (ref. higher education) | .077 | 0.012 | 6.367 | < .001 |
|  |  | Deprivation | -.010 | 0.007 | -1.461 | .144 |
|  |  | Population Density | .032 | 0.008 | 4.009 | < .001 |
| *Individual-Level* | | |  |  |  |  |
|  |  | Extraversion | .019 | 0.006 | 3.346 | .001 |
|  |  | Agreeableness | .005 | 0.006 | 0.786 | .432 |
|  |  | Openness | .059 | 0.006 | 9.584 | < .001 |
|  |  | Neuroticism | -.002 | 0.006 | -0.368 | .713 |
|  |  | Conscientiousness | .004 | 0.006 | 0.578 | .563 |
|  |  | Preference Conservative Party | -.025 | 0.006 | -4.407 | < .001 |
|  |  | Neighbourhood Belonging | .007 | 0.005 | 1.300 | .194 |
|  |  | Perceived Racial Hate Crime Incidence | .020 | 0.005 | 4.364 | < .001 |
| *Contextual-Level* | | |  |  |  |  |
|  |  | Contact Opportunities | .299 | 0.007 | 40.692 | < .001 |
|  |  | Actual Racial Hate Crime Incidence~~s~~ | .029 | 0.010 | 2.940 | .003 |
|  |  | Right-Wing Votes | -.003 | 0.011 | -0.301 | .764 |
|  |  | Brexit LeaveVotes | -.039 | 0.013 | -2.967 | .003 |

*Note*. n individual-level = 18806; n LSOA level = 11181; n LAD level = 320

***Cross-level interactions***

In the next step, we added the cross-level interactions between the individual-level variables and the contextual-level variables to the main-effects only model. Again, we do not yet include group membership in this model and hence effects reflect the effects for the entire sample.

**Table S5**

*Results cross-level interaction model*

|  |  |  | *β* | *se* | *t* | *p* |
| --- | --- | --- | --- | --- | --- | --- |
|  |  | *Intercept* | .065 | 0.016 | 4.096 | < .001 |
|  |  | *Time* | -.108 | 0.007 | -16.297 | < .001 |
| *Control* | | |  |  |  |  |
|  |  | Age | -.108 | 0.007 | -16.297 | < .001 |
|  |  | Gender (ref. male) | -.026 | 0.011 | -2.308 | .021 |
|  |  | Income | .007 | 0.005 | 1.361 | .173 |
|  |  | Education Post Secondary (ref. higher education) | .055 | 0.017 | 3.276 | .001 |
|  |  | Education Secondary (ref. higher education) | .075 | 0.012 | -6.181 | < .001 |
|  |  | Deprivation | -.010 | 0.007 | -1.452 | .146 |
|  |  | Population Density | .031 | 0.008 | 3.855 | < .001 |
| *Individual-Level* | | |  |  |  |  |
|  |  | Extraversion | .019 | 0.006 | 3.263 | .001 |
|  |  | Agreeableness | .006 | 0.006 | 1.023 | .306 |
|  |  | Openness | .059 | 0.006 | 9.557 | < .001 |
|  |  | Neuroticism | -.004 | 0.006 | -0.647 | .518 |
|  |  | Conscientiousness | .002 | 0.006 | 0.279 | .780 |
|  |  | Preference Conservative Party | -.026 | 0.006 | -4.408 | < .001 |
|  |  | Neighbourhood Belonging | .007 | 0.005 | 1.347 | .178 |
|  |  | Perceived Racial Hate Crime Incidence | .025 | 0.005 | 5.037 | < .001 |
| *Contextual-Level* | | |  |  |  |  |
|  |  | Contact Opportunities | .316 | 0.008 | 40.770 | < .001 |
|  |  | Actual Racial Hate Crime Incidence | .028 | 0.010 | 2.813 | .005 |
|  |  | Right-Wing Votes | -.004 | 0.011 | -0.414 | .679 |
|  |  | Brexit LeaveVotes | -.039 | 0.013 | -2.930 | .004 |
| *Cross-level interactions* | | |  |  |  |  |
|  | *Contact opportunities* | |  |  |  |  |
|  |  | x Extraversion | .018 | 0.007 | 2.635 | .008 |
|  |  | x Agreeableness | -.007 | 0.007 | -1.059 | .289 |
|  |  | x Openness | -.011 | 0.007 | -1.474 | .141 |
|  |  | x Neuroticism | .018 | 0.007 | 2.539 | .011 |
|  |  | x Conscientiousness | .017 | 0.007 | 2.373 | .018 |
|  |  | x Preference Conservative Party | .028 | 0.007 | 4.090 | < .001 |
|  |  | x Neighbourhood Belonging | .000 | 0.006 | 0.022 | .982 |
|  |  | x Perceived Racial Hate Crime Incidence | .017 | 0.005 | 3.683 | < .001 |
|  | *Actual Racial Hate Crime Incidence* | |  |  |  |  |
|  |  | x Extraversion | .004 | 0.007 | 0.522 | .602 |
|  |  | x Agreeableness | -.001 | 0.007 | -0.112 | .911 |
|  |  | x Openness | -.002 | 0.007 | -0.334 | .738 |
|  |  | x Neuroticism | -.001 | 0.007 | -0.130 | .896 |
|  |  | x Conscientiousness | -.004 | 0.007 | -0.552 | .581 |
|  |  | x Preference Conservative Party | .000 | 0.007 | 0.034 | .973 |
|  |  | x Neighbourhood Belonging | .008 | 0.006 | 1.342 | .180 |
|  |  | x Perceived Racial Hate Crime Incidence | -.004 | 0.005 | -0.886 | .376 |
|  | *Right-wing Votes* | |  |  |  |  |
|  |  | x Extraversion | -.004 | 0.008 | -0.447 | .655 |
|  |  | x Agreeableness | -.002 | 0.008 | -0.279 | .781 |
|  |  | x Openness | .011 | 0.008 | 1.390 | .164 |
|  |  | x Neuroticism | .001 | 0.008 | 0.121 | .904 |
|  |  | x Conscientiousness | -.012 | 0.008 | -1.453 | .146 |
|  |  | x Preference Conservative Party | .007 | 0.008 | 0.945 | .345 |
|  |  | x Neighbourhood Belonging | -.006 | 0.007 | -0.838 | .402 |
|  |  | x Perceived Racial Hate Crime Incidence | .005 | 0.006 | 0.778 | .437 |
|  | *Brexit LeaveVote* | |  |  |  |  |
|  |  | x Extraversion | -.009 | 0.009 | -1.021 | .307 |
|  |  | x Agreeableness | -.008 | 0.009 | -0.872 | .383 |
|  |  | x Openness | -.006 | 0.009 | -0.617 | .537 |
|  |  | x Neuroticism | .009 | 0.009 | 1.066 | .287 |
|  |  | x Conscientiousness | .027 | 0.009 | 2.865 | .004 |
|  |  | x Preference Conservative Party | .003 | 0.009 | 0.381 | .703 |
|  |  | x Neighbourhood Belonging | .001 | 0.008 | 0.090 | .928 |
|  |  | x Perceived Racial Hate Crime Incidence | -.006 | 0.007 | -0.851 | .395 |

*Note*. n individual=level = 18806; n LSOA level = 11181; n LAD level = 320; all estimates are based on standardised regression coefficients

***Separate group models***

Next, we ran the main-effects only and the cross-level interaction model separately for Asian, Black, and White participants. These models provide interesting information but should not be overinterpreted with regards to group differences. Presence or absence of significant effects in these models does not mean that these effects differ between groups since we did not formally test the group interactions (as we do in the main analysis). In addition, to facilitate comparisons between these analyses and the main analysis reported, we also extended the group separate models on a combined minority group sample (i.e., including Black and Asian participants). We ran the main-effects only and cross-level interaction model for the two minority groups combined. See Table S6-S9 for the results.

**Table S6**

*Results for Majority (White) participants*

|  |  |  | Main-effects only | | | | Cross-level interactions | | | |
| --- | --- | --- | --- | --- | --- | --- | --- | --- | --- | --- |
|  |  |  | *β* | *se* | *t* | *p* | *β* | *se* | *t* | *p* |
|  |  | *Intercept* | .058 | 0.017 | 3.449 | .001 | .075 | 0.017 | 4.422 | < .001 |
|  |  | *Time* | -.003 | 0.005 | -0.520 | .603 | -.002 | 0.005 | -0.467 | .640 |
| *Control* | | |  |  |  |  |  |  |  |  |
|  |  | Age | -.106 | 0.007 | -15.914 | < .001 | -.106 | 0.007 | -15.827 | < .001 |
|  |  | Gender (ref. male) | -.029 | 0.012 | -2.508 | .012 | -.030 | 0.012 | -2.559 | .011 |
|  |  | Income | .004 | 0.005 | 0.772 | .440 | .004 | 0.005 | 0.782 | .434 |
|  |  | Education Post Secondary (ref. Secondary) | .045 | 0.017 | 2.622 | .009 | .044 | 0.017 | 2.579 | .010 |
|  |  | Education Higher (ref. Secondary) | .081 | 0.012 | 6.647 | < .001 | .081 | 0.012 | 6.666 | < .001 |
|  |  | Deprivation | -.027 | 0.007 | -3.884 | < .001 | -.025 | 0.007 | -3.695 | < .001 |
|  |  | Population Density | .034 | 0.009 | 3.615 | < .001 | .034 | 0.009 | 3.575 | < .001 |
| *Individual-Level* | | |  |  |  |  |  |  |  |  |
|  |  | Extraversion | .008 | 0.006 | 1.304 | .192 | .005 | 0.007 | 0.765 | .445 |
|  |  | Agreeableness | .009 | 0.006 | 1.460 | .144 | .018 | 0.007 | 2.595 | .009 |
|  |  | Openness | .061 | 0.006 | 9.799 | < .001 | .050 | 0.007 | 6.989 | < .001 |
|  |  | Neuroticism | -.009 | 0.006 | -1.567 | .117 | -.015 | 0.007 | -2.221 | .026 |
|  |  | Conscientiousness | .002 | 0.006 | 0.333 | .739 | .006 | 0.007 | 0.842 | .400 |
|  |  | Preference Conservative Party | -.035 | 0.006 | -6.057 | < .001 | -.031 | 0.007 | -4.592 | < .001 |
|  |  | Neighbourhood Belonging | .013 | 0.005 | 2.413 | .016 | .023 | 0.006 | 3.878 | < .001 |
|  |  | Perceived Racial Hate Crime Incidence | .028 | 0.005 | 5.408 | < .001 | .026 | 0.005 | 4.853 | < .001 |
| *Contextual-Level* | | |  |  |  |  |  |  |  |  |
|  |  | Contact Opportunities | .241 | 0.018 | 13.704 | < .001 | .284 | 0.019 | 15.018 | < .001 |
|  |  | Actual Racial Hate Crime Incidence | .042 | 0.012 | 3.566 | < .001 | .038 | 0.012 | 3.189 | .001 |
|  |  | Right-Wing Votes | .002 | 0.011 | 0.202 | .840 | .002 | 0.011 | 0.189 | .850 |
|  |  | Brexit *Leave* Votes | -.048 | 0.014 | -3.377 | .001 | -.047 | 0.014 | -3.328 | .001 |
| *Cross-level interactions* | | |  |  |  |  |  |  |  |  |
|  | *Contact opportunities* | |  |  |  |  |  |  |  |  |
|  |  | x Extraversion |  |  |  |  | -.022 | 0.015 | -1.515 | .130 |
|  |  | x Agreeableness |  |  |  |  | .028 | 0.014 | 1.946 | .052 |
|  |  | x Openness |  |  |  |  | -.032 | 0.015 | -2.145 | .032 |
|  |  | x Neuroticism |  |  |  |  | -.016 | 0.014 | -1.107 | .268 |
|  |  | x Conscientiousness |  |  |  |  | .039 | 0.015 | 2.557 | .011 |
|  |  | x Preference Conservative Party |  |  |  |  | .019 | 0.014 | 1.352 | .176 |
|  |  | x Neighbourhood Belonging |  |  |  |  | .043 | 0.012 | 3.541 | < .001 |
|  |  | x Perceived Racial Hate Crime Incidence |  |  |  |  | -.016 | 0.010 | -1.657 | .098 |
|  | Actual Racial Hate Crime Incidence | |  |  |  |  |  |  |  |  |
|  |  | x Extraversion |  |  |  |  | .010 | 0.009 | 1.126 | .260 |
|  |  | x Agreeableness |  |  |  |  | -.010 | 0.009 | -1.155 | .248 |
|  |  | x Openness |  |  |  |  | -.001 | 0.009 | -0.115 | .908 |
|  |  | x Neuroticism |  |  |  |  | .009 | 0.009 | 0.963 | .336 |
|  |  | x Conscientiousness |  |  |  |  | -.010 | 0.009 | -1.121 | .262 |
|  |  | x Preference Conservative Party |  |  |  |  | -.010 | 0.008 | -1.205 | .228 |
|  |  | x Neighbourhood Belonging |  |  |  |  | .014 | 0.007 | 1.817 | .069 |
|  |  | x Perceived Racial Hate Crime Incidence |  |  |  |  | .007 | 0.007 | 1.072 | .284 |
|  | *Right-Wing Votes* | |  |  |  |  |  |  |  |  |
|  |  | x Extraversion |  |  |  |  | -.004 | 0.008 | -0.441 | .659 |
|  |  | x Agreeableness |  |  |  |  | -.000 | 0.009 | -0.041 | .967 |
|  |  | x Openness |  |  |  |  | .012 | 0.008 | 1.437 | .151 |
|  |  | x Neuroticism |  |  |  |  | -.011 | 0.008 | -1.386 | .166 |
|  |  | x Conscientiousness |  |  |  |  | -.015 | 0.009 | -1.743 | .081 |
|  |  | x Preference Conservative Party |  |  |  |  | .014 | 0.008 | 1.727 | .084 |
|  |  | x Neighbourhood Belonging |  |  |  |  | -.005 | 0.007 | -0.686 | .493 |
|  |  | x Perceived Racial Hate Crime Incidence |  |  |  |  | .003 | 0.007 | 0.454 | .650 |
|  | *Brexit Leave Votes* | |  |  |  |  |  |  |  |  |
|  |  | x Extraversion |  |  |  |  | -.012 | 0.009 | -1.294 | .196 |
|  |  | x Agreeableness |  |  |  |  | -.014 | 0.010 | -1.462 | .144 |
|  |  | x Openness |  |  |  |  | .001 | 0.010 | 0.093 | .926 |
|  |  | x Neuroticism |  |  |  |  | .018 | 0.009 | 1.960 | .050 |
|  |  | x Conscientiousness |  |  |  |  | .035 | 0.010 | 3.527 | < .001 |
|  |  | x Preference Conservative Party |  |  |  |  | -.001 | 0.009 | -0.115 | .908 |
|  |  | x Neighbourhood Belonging |  |  |  |  | .014 | 0.008 | 1.748 | .081 |
|  |  | x Perceived Racial Hate Crime Incidence |  |  |  |  | -.003 | 0.008 | -0.405 | .685 |

*Note*. N individual-level = 16603; n LSOA level = 9946; n LAD level = 320; all estimates are based on standardised regression coefficients

**Table S7**

*Results for Asian participants*

|  |  |  | Main-effects only | | | | Cross-level interactions | | | |
| --- | --- | --- | --- | --- | --- | --- | --- | --- | --- | --- |
|  |  |  | *β* | *se* | *t* | *p* | *β* | *se* | *t* | *p* |
|  |  | *Intercept* | .195 | 0.109 | 1.785 | .075 | .140 | 0.116 | 1.208 | .228 |
|  |  | *Time* | .033 | 0.023 | 1.396 | .163 | .033 | 0.023 | 1.414 | .157 |
| *Control* | | |  |  |  |  |  |  |  |  |
|  |  | Age | -.170 | 0.037 | -4.607 | < .001 | -.182 | 0.037 | -4.849 | < .001 |
|  |  | Gender (ref. male) | .009 | 0.050 | 0.187 | .851 | -.001 | 0.050 | -0.012 | .990 |
|  |  | Income | .136 | 0.041 | 3.353 | .001 | .140 | 0.041 | 3.414 | .001 |
|  |  | Education Post Secondary (ref. Secondary) | .194 | 0.069 | 2.821 | .005 | .200 | 0.069 | 2.885 | .004 |
|  |  | Education Higher (ref. Secondary) | .130 | 0.060 | 2.171 | .030 | .133 | 0.060 | 2.198 | .028 |
|  |  | Deprivation | .032 | 0.033 | 0.996 | .319 | .026 | 0.033 | 0.800 | .424 |
|  |  | Population Density | .036 | 0.025 | 1.407 | .160 | .036 | 0.026 | 1.414 | .158 |
| *Individual-Level* | | |  |  |  |  |  |  |  |  |
|  |  | Extraversion | .072 | 0.027 | 2.643 | .008 | .080 | 0.067 | 1.202 | .230 |
|  |  | Agreeableness | -.013 | 0.026 | -0.478 | .633 | -.011 | 0.062 | -0.179 | .858 |
|  |  | Openness | .043 | 0.029 | 1.480 | .139 | .035 | 0.069 | 0.510 | .610 |
|  |  | Neuroticism | .016 | 0.027 | 0.598 | .550 | .021 | 0.062 | 0.342 | .732 |
|  |  | Conscientiousness | .053 | 0.027 | 1.974 | .049 | .013 | 0.061 | 0.209 | .835 |
|  |  | Preference Conservative Party | .033 | 0.027 | 1.235 | .217 | .025 | 0.063 | 0.392 | .695 |
|  |  | Neighbourhood Belonging | -.061 | 0.021 | -2.962 | .003 | -.098 | 0.050 | -1.980 | .048 |
|  |  | Perceived Racial Hate Crime Incidence | -.009 | 0.014 | -0.619 | .536 | .014 | 0.033 | 0.419 | .675 |
| *Contextual-Level* | | |  |  |  |  |  |  |  |  |
|  |  | Contact Opportunities | .239 | 0.033 | 7.178 | < .001 | .255 | 0.037 | 6.950 | < .001 |
|  |  | Actual Racial Hate Crime Incidence | -.004 | 0.026 | -0.150 | .881 | -.001 | 0.027 | -0.052 | .959 |
|  |  | Right-Wing Votes | -.031 | 0.038 | -0.822 | .412 | -.064 | 0.042 | -1.531 | .127 |
|  |  | Brexit *Leave* Votes | -.057 | 0.048 | -1.201 | .231 | -.053 | 0.052 | -1.022 | .308 |
| *Cross-level interactions* | | |  |  |  |  |  |  |  |  |
|  | *Contact opportunities* | |  |  |  |  |  |  |  |  |
|  |  | x Extraversion |  |  |  |  | -.015 | 0.028 | -0.560 | .575 |
|  |  | x Agreeableness |  |  |  |  | -.002 | 0.026 | -0.082 | .935 |
|  |  | x Openness |  |  |  |  | .011 | 0.029 | 0.395 | .693 |
|  |  | x Neuroticism |  |  |  |  | .011 | 0.026 | 0.415 | .678 |
|  |  | x Conscientiousness |  |  |  |  | .016 | 0.026 | 0.627 | .531 |
|  |  | x Preference Conservative Party |  |  |  |  | -.001 | 0.027 | -0.047 | .963 |
|  |  | x Neighbourhood Belonging |  |  |  |  | .031 | 0.021 | 1.515 | .130 |
|  |  | x Perceived Racial Hate Crime Incidence |  |  |  |  | -.010 | 0.015 | -0.707 | .479 |
|  | Actual Racial Hate Crime Incidence | |  |  |  |  |  |  |  |  |
|  |  | x Extraversion |  |  |  |  | .003 | 0.020 | 0.146 | .884 |
|  |  | x Agreeableness |  |  |  |  | .028 | 0.019 | 1.462 | .144 |
|  |  | x Openness |  |  |  |  | -.041 | 0.021 | -1.943 | .052 |
|  |  | x Neuroticism |  |  |  |  | -.028 | 0.020 | -1.414 | .158 |
|  |  | x Conscientiousness |  |  |  |  | .013 | 0.021 | 0.630 | .529 |
|  |  | x Preference Conservative Party |  |  |  |  | .010 | 0.020 | 0.514 | .607 |
|  |  | x Neighbourhood Belonging |  |  |  |  | -.039 | 0.017 | -2.377 | .018 |
|  |  | x Perceived Racial Hate Crime Incidence |  |  |  |  | .001 | 0.011 | 0.108 | .914 |
|  | *Right-Wing Votes* | |  |  |  |  |  |  |  |  |
|  |  | x Extraversion |  |  |  |  | .002 | 0.032 | 0.051 | .959 |
|  |  | x Agreeableness |  |  |  |  | -.004 | 0.031 | -0.113 | .910 |
|  |  | x Openness |  |  |  |  | -.000 | 0.035 | -0.014 | .989 |
|  |  | x Neuroticism |  |  |  |  | .067 | 0.034 | 1.968 | .049 |
|  |  | x Conscientiousness |  |  |  |  | -.015 | 0.031 | -0.492 | .622 |
|  |  | x Preference Conservative Party |  |  |  |  | -.028 | 0.036 | -0.768 | .443 |
|  |  | x Neighbourhood Belonging |  |  |  |  | -.002 | 0.025 | -0.069 | .945 |
|  |  | x Perceived Racial Hate Crime Incidence |  |  |  |  | .010 | 0.016 | 0.621 | .534 |
|  | *Brexit Leave Votes* | |  |  |  |  |  |  |  |  |
|  |  | x Extraversion |  |  |  |  | -.059 | 0.038 | -1.543 | .123 |
|  |  | x Agreeableness |  |  |  |  | .075 | 0.038 | 1.987 | .047 |
|  |  | x Openness |  |  |  |  | -.057 | 0.040 | -1.437 | .151 |
|  |  | x Neuroticism |  |  |  |  | -.084 | 0.040 | -2.109 | .035 |
|  |  | x Conscientiousness |  |  |  |  | .005 | 0.038 | 0.124 | .901 |
|  |  | x Preference Conservative Party |  |  |  |  | .021 | 0.041 | 0.520 | .603 |
|  |  | x Neighbourhood Belonging |  |  |  |  | -.055 | 0.032 | -1.727 | .084 |
|  |  | x Perceived Racial Hate Crime Incidence |  |  |  |  | .002 | 0.020 | 0.095 | .925 |

*Note.* n individual-level = 1490; n LSOA level = 1163; n LAD level = 185; all estimates are based on standardised regression coefficients

**Table S8**

*Results for Black participants*

|  |  |  | Main-effects only | | | | Cross-level interactions | | | |
| --- | --- | --- | --- | --- | --- | --- | --- | --- | --- | --- |
|  |  |  | *β* | *se* | *t* | *p* | *β* | *se* | *t* | *p* |
|  |  | *Intercept* | .318 | 0.285 | 1.117 | .265 | .382 | 0.346 | 1.102 | .271 |
|  |  | *Time* | .045 | 0.036 | 1.261 | .208 | .059 | 0.036 | 1.661 | .097 |
| *Control* | | |  |  |  |  |  |  |  |  |
|  |  | Age | -.091 | 0.046 | -1.986 | .047 | -.095 | 0.046 | -2.064 | .039 |
|  |  | Gender (ref. male) | .006 | 0.070 | 0.091 | .927 | -.013 | 0.070 | -0.188 | .851 |
|  |  | Income | -.066 | 0.068 | -0.981 | .327 | -.066 | 0.068 | -0.972 | .331 |
|  |  | Education Post Secondary (ref. Secondary) | .043 | 0.100 | 0.426 | .670 | .018 | 0.101 | 0.179 | .858 |
|  |  | Education Higher (ref. Secondary) | -.049 | 0.076 | -0.651 | .515 | -.034 | 0.076 | -0.447 | .655 |
|  |  | Deprivation | .177 | 0.051 | 3.500 | .001 | .173 | 0.051 | 3.422 | .001 |
|  |  | Population Density | .053 | 0.031 | 1.715 | .087 | .041 | 0.031 | 1.342 | .180 |
| *Individual-Level* | | |  |  |  |  |  |  |  |  |
|  |  | Extraversion | .109 | 0.033 | 3.267 | .001 | .068 | 0.225 | 0.302 | .763 |
|  |  | Agreeableness | -.039 | 0.035 | -1.109 | .268 | .179 | 0.238 | 0.753 | .452 |
|  |  | Openness | .066 | 0.038 | 1.753 | .080 | .088 | 0.239 | 0.370 | .711 |
|  |  | Neuroticism | .086 | 0.039 | 2.214 | .027 | .022 | 0.263 | 0.084 | .933 |
|  |  | Conscientiousness | -.009 | 0.038 | -0.249 | .803 | .014 | 0.235 | 0.060 | .952 |
|  |  | Preference Conservative Party | .061 | 0.036 | 1.686 | .092 | .019 | 0.220 | 0.086 | .931 |
|  |  | Neighbourhood Belonging | .053 | 0.028 | 1.864 | .062 | .077 | 0.182 | 0.421 | .674 |
|  |  | Perceived Racial Hate Crime Incidence | .024 | 0.022 | 1.128 | .259 | -.220 | 0.148 | -1.483 | .138 |
| *Contextual-Level* | | |  |  |  |  |  |  |  |  |
|  |  | Contact Opportunities | .291 | 0.087 | 3.349 | .001 | .265 | 0.110 | 2.405 | .017 |
|  |  | Actual Racial Hate Crime Incidence | -.030 | 0.033 | -0.915 | .361 | -.064 | 0.038 | -1.687 | .092 |
|  |  | Right-Wing Votes | -.057 | 0.044 | -1.282 | .203 | -.200 | 0.063 | -3.174 | .002 |
|  |  | Brexit *Leave* Votes | .015 | 0.053 | 0.279 | .781 | .078 | 0.064 | 1.216 | .224 |
| *Cross-level interactions* | | |  |  |  |  |  |  |  |  |
|  | *Contact opportunities* | |  |  |  |  |  |  |  |  |
|  |  | x Extraversion |  |  |  |  | .037 | 0.075 | 0.497 | .620 |
|  |  | x Agreeableness |  |  |  |  | -.077 | 0.079 | -0.983 | .326 |
|  |  | x Openness |  |  |  |  | -.038 | 0.080 | -0.480 | .631 |
|  |  | x Neuroticism |  |  |  |  | .038 | 0.087 | 0.432 | .666 |
|  |  | x Conscientiousness |  |  |  |  | -.002 | 0.079 | -0.021 | .984 |
|  |  | x Preference Conservative Party |  |  |  |  | -.007 | 0.074 | -0.097 | .923 |
|  |  | x Neighbourhood Belonging |  |  |  |  | -.020 | 0.061 | -0.334 | .738 |
|  |  | x Perceived Racial Hate Crime Incidence |  |  |  |  | .076 | 0.049 | 1.549 | .122 |
|  | Actual Racial Hate Crime Incidence | |  |  |  |  |  |  |  |  |
|  |  | x Extraversion |  |  |  |  | -.021 | 0.023 | -0.910 | .363 |
|  |  | x Agreeableness |  |  |  |  | .004 | 0.027 | 0.159 | .874 |
|  |  | x Openness |  |  |  |  | .023 | 0.026 | 0.881 | .378 |
|  |  | x Neuroticism |  |  |  |  | -.013 | 0.028 | -0.471 | .638 |
|  |  | x Conscientiousness |  |  |  |  | -.008 | 0.026 | -0.301 | .764 |
|  |  | x Preference Conservative Party |  |  |  |  | .005 | 0.025 | 0.194 | .846 |
|  |  | x Neighbourhood Belonging |  |  |  |  | .002 | 0.022 | 0.085 | .932 |
|  |  | x Perceived Racial Hate Crime Incidence |  |  |  |  | .017 | 0.019 | 0.889 | .374 |
|  | *Right-Wing Votes* | |  |  |  |  |  |  |  |  |
|  |  | x Extraversion |  |  |  |  | -.019 | 0.035 | -0.536 | .592 |
|  |  | x Agreeableness |  |  |  |  | .028 | 0.048 | 0.577 | .564 |
|  |  | x Openness |  |  |  |  | .105 | 0.047 | 2.226 | .026 |
|  |  | x Neuroticism |  |  |  |  | .090 | 0.042 | 2.124 | .034 |
|  |  | x Conscientiousness |  |  |  |  | .016 | 0.047 | 0.347 | .728 |
|  |  | x Preference Conservative Party |  |  |  |  | -.124 | 0.046 | -2.704 | .007 |
|  |  | x Neighbourhood Belonging |  |  |  |  | -.028 | 0.032 | -0.872 | .383 |
|  |  | x Perceived Racial Hate Crime Incidence |  |  |  |  | -.011 | 0.022 | -0.505 | .614 |
|  | *Brexit Leave Votes* | |  |  |  |  |  |  |  |  |
|  |  | x Extraversion |  |  |  |  | .036 | 0.043 | 0.842 | .400 |
|  |  | x Agreeableness |  |  |  |  | .002 | 0.049 | 0.032 | .975 |
|  |  | x Openness |  |  |  |  | -.125 | 0.051 | -2.461 | .014 |
|  |  | x Neuroticism |  |  |  |  | -.059 | 0.049 | -1.212 | .226 |
|  |  | x Conscientiousness |  |  |  |  | .000 | 0.052 | 0.004 | .997 |
|  |  | x Preference Conservative Party |  |  |  |  | .052 | 0.049 | 1.059 | .290 |
|  |  | x Neighbourhood Belonging |  |  |  |  | -.025 | 0.037 | -0.693 | .488 |
|  |  | x Perceived Racial Hate Crime Incidence |  |  |  |  | -.008 | 0.027 | -0.304 | .762 |

*Note*. The model including interactions did not converge. Changing the random effects structure to include the MSOA level instead of the LSOA and LAD level resulted in model convergence. n individual-level = 713; n LSOA level = 657; n LAD level = 110; n MSOA = 525; all estimates are based on standardised regression coefficients

**Table S9**

*Results for minority group participants*

|  |  |  | Main-effects only | | | | Cross-level interactions | | | |
| --- | --- | --- | --- | --- | --- | --- | --- | --- | --- | --- |
|  |  |  | *β* | *se* | *t* | *p* | *β* | *se* | *t* | *p* |
|  |  | *Intercept* | .234 | 0.091 | 2.567 | .010 | .198 | 0.097 | 2.047 | .041 |
|  |  | *Time* | .037 | 0.020 | 1.864 | .063 | .037 | 0.020 | 1.871 | .062 |
| *Control* | | |  |  |  |  |  |  |  |  |
|  |  | Age | -.135 | 0.029 | -4.694 | < .001 | -.142 | 0.029 | -4.936 | < .001 |
|  |  | Gender (ref. male) | -.011 | 0.041 | -0.262 | .794 | -.013 | 0.041 | -0.329 | .742 |
|  |  | Income | .091 | 0.035 | 2.614 | .009 | .091 | 0.035 | 2.609 | .009 |
|  |  | Education Post Secondary (ref. Secondary) | .142 | 0.057 | 2.495 | .013 | .137 | 0.057 | 2.405 | .016 |
|  |  | Education Higher (ref. Secondary) | .056 | 0.047 | 1.200 | .230 | .059 | 0.047 | 1.242 | .214 |
|  |  | Deprivation | .085 | 0.026 | 3.233 | .001 | .082 | 0.027 | 3.080 | .002 |
|  |  | Population Density | .046 | 0.019 | 2.361 | .018 | .044 | 0.019 | 2.274 | .023 |
| *Individual-Level* | | |  |  |  |  |  |  |  |  |
|  |  | Extraversion | .091 | 0.021 | 4.255 | < .001 | .070 | 0.062 | 1.114 | .265 |
|  |  | Agreeableness | -.017 | 0.021 | -0.808 | .419 | .038 | 0.058 | 0.647 | .518 |
|  |  | Openness | .054 | 0.023 | 2.343 | .019 | .045 | 0.064 | 0.706 | .480 |
|  |  | Neuroticism | .043 | 0.022 | 1.929 | .054 | .010 | 0.058 | 0.173 | .863 |
|  |  | Conscientiousness | .023 | 0.022 | 1.054 | .292 | .021 | 0.057 | 0.361 | .718 |
|  |  | Preference Conservative Party | .043 | 0.022 | 1.967 | .049 | .023 | 0.058 | 0.392 | .695 |
|  |  | Neighbourhood Belonging | -.023 | 0.017 | -1.386 | .166 | -.122 | 0.046 | -2.642 | .008 |
|  |  | Perceived Racial Hate Crime Incidence | .002 | 0.012 | 0.207 | .836 | -.002 | 0.031 | -0.065 | .948 |
| *Contextual-Level* | | |  |  |  |  |  |  |  |  |
|  |  | Contact Opportunities | .257 | 0.026 | 9.707 | < .001 | .269 | 0.030 | 9.063 | < .001 |
|  |  | Actual Racial Hate Crime Incidence | -.014 | 0.021 | -0.662 | .509 | -.017 | 0.022 | -0.775 | .439 |
|  |  | Right-Wing Votes | -.034 | 0.030 | -1.114 | .267 | -.068 | 0.035 | -1.965 | .050 |
|  |  | Brexit *Leave* Votes | -.023 | 0.038 | -0.617 | .538 | -.017 | 0.041 | -0.419 | .676 |
| *Cross-level interactions* | | |  |  |  |  |  |  |  |  |
|  | *Contact opportunities* | |  |  |  |  |  |  |  |  |
|  |  | x Extraversion |  |  |  |  | .006 | 0.024 | 0.245 | .807 |
|  |  | x Agreeableness |  |  |  |  | -.024 | 0.023 | -1.057 | .291 |
|  |  | x Openness |  |  |  |  | .000 | 0.025 | -0.002 | .999 |
|  |  | x Neuroticism |  |  |  |  | .023 | 0.023 | 1.005 | .315 |
|  |  | x Conscientiousness |  |  |  |  | .003 | 0.023 | 0.154 | .878 |
|  |  | x Preference Conservative Party |  |  |  |  | -.002 | 0.023 | -0.089 | .929 |
|  |  | x Neighbourhood Belonging |  |  |  |  | .044 | 0.018 | 2.460 | .014 |
|  |  | x Perceived Racial Hate Crime Incidence |  |  |  |  | .000 | 0.013 | 0.019 | .985 |
|  | Actual Racial Hate Crime Incidence | |  |  |  |  |  |  |  |  |
|  |  | x Extraversion |  |  |  |  | -.003 | 0.015 | -0.189 | .850 |
|  |  | x Agreeableness |  |  |  |  | .014 | 0.015 | 0.919 | .358 |
|  |  | x Openness |  |  |  |  | -.014 | 0.016 | -0.846 | .398 |
|  |  | x Neuroticism |  |  |  |  | -.017 | 0.016 | -1.093 | .274 |
|  |  | x Conscientiousness |  |  |  |  | -.003 | 0.016 | -0.163 | .870 |
|  |  | x Preference Conservative Party |  |  |  |  | .005 | 0.015 | 0.305 | .760 |
|  |  | x Neighbourhood Belonging |  |  |  |  | -.020 | 0.013 | -1.544 | .123 |
|  |  | x Perceived Racial Hate Crime Incidence |  |  |  |  | .002 | 0.010 | 0.179 | .858 |
|  | *Right-Wing Votes* | |  |  |  |  |  |  |  |  |
|  |  | x Extraversion |  |  |  |  | .000 | 0.023 | 0.011 | .991 |
|  |  | x Agreeableness |  |  |  |  | .006 | 0.026 | 0.238 | .812 |
|  |  | x Openness |  |  |  |  | .021 | 0.027 | 0.781 | .435 |
|  |  | x Neuroticism |  |  |  |  | .084 | 0.026 | 3.183 | .001 |
|  |  | x Conscientiousness |  |  |  |  | -.004 | 0.025 | -0.154 | .878 |
|  |  | x Preference Conservative Party |  |  |  |  | -.056 | 0.027 | -2.031 | .042 |
|  |  | x Neighbourhood Belonging |  |  |  |  | -.010 | 0.019 | -0.520 | .603 |
|  |  | x Perceived Racial Hate Crime Incidence |  |  |  |  | .005 | 0.013 | 0.369 | .712 |
|  | *Brexit Leave Votes* | |  |  |  |  |  |  |  |  |
|  |  | x Extraversion |  |  |  |  | -.022 | 0.027 | -0.801 | .423 |
|  |  | x Agreeableness |  |  |  |  | .031 | 0.029 | 1.062 | .288 |
|  |  | x Openness |  |  |  |  | -.062 | 0.031 | -2.022 | .043 |
|  |  | x Neuroticism |  |  |  |  | -.083 | 0.030 | -2.750 | .006 |
|  |  | x Conscientiousness |  |  |  |  | -.001 | 0.030 | -0.044 | .965 |
|  |  | x Preference Conservative Party |  |  |  |  | .023 | 0.030 | 0.765 | .444 |
|  |  | x Neighbourhood Belonging |  |  |  |  | -.051 | 0.023 | -2.196 | .028 |
|  |  | x Perceived Racial Hate Crime Incidence |  |  |  |  | -.003 | 0.016 | -0.187 | .851 |

*Note*. n individual-level = 2203; n LSOA level = 1748; n LAD level = 201; all estimates are based on standardised regression coefficients

**Full three-group model**

Finally, we included group membership (Asian, Black, White) as a moderator to the model with the cross-level interactions. The reference group was White participants. Main effects and cross-level interactions thus reflect the effects for White participants. See Table S10 for the results.

**Table S10**

*Results of fully-interacted three-group model*

|  |  |  | *β* | *se* | *t* | *p* |
| --- | --- | --- | --- | --- | --- | --- |
|  |  | *Intercept* | .074 | 0.017 | 4.360 | < .001 |
|  |  | *Time* | -.003 | 0.005 | -0.492 | .623 |
|  |  | *Group Asian (ref. White)* | .072 | 0.084 | 0.853 | .393 |
|  |  | *Group Black (ref. White)* | .137 | 0.299 | 0.458 | .647 |
| *Control* | | |  |  |  |  |
|  |  | Age | -.106 | 0.007 | -15.224 | < .001 |
|  |  | Gender (ref. male) | -.030 | 0.012 | -2.479 | .013 |
|  |  | Income | .004 | 0.005 | 0.806 | .420 |
|  |  | Education Post Secondary (ref. Secondary) | .044 | 0.018 | 2.453 | .014 |
|  |  | Education Higher (ref. Secondary) | .080 | 0.013 | 6.278 | < .001 |
|  |  | Deprivation | -.023 | 0.007 | -3.245 | .001 |
|  |  | Population Density | .034 | 0.010 | 3.531 | < .001 |
| *Individual-Level* | | |  |  |  |  |
|  |  | Extraversion | .006 | 0.007 | 0.796 | .426 |
|  |  | Agreeableness | .019 | 0.007 | 2.588 | .010 |
|  |  | Openness | .050 | 0.008 | 6.653 | < .001 |
|  |  | Neuroticism | -.015 | 0.007 | -2.075 | .038 |
|  |  | Conscientiousness | .006 | 0.008 | 0.853 | .394 |
|  |  | Preference Conservative Party | -.030 | 0.007 | -4.267 | < .001 |
|  |  | Neighbourhood Belonging | .022 | 0.006 | 3.553 | < .001 |
|  |  | Perceived Racial Hate Crime Incidence | .026 | 0.006 | 4.709 | < .001 |
| *Contextual-Level* | | |  |  |  |  |
|  |  | Contact Opportunities | .290 | 0.019 | 15.117 | < .001 |
|  |  | Actual Racial Hate Crimes Incidence | .040 | 0.012 | 3.379 | .001 |
|  |  | Right-Wing Votes | .003 | 0.011 | 0.289 | .773 |
|  |  | Brexit Leave Votes | -.044 | 0.014 | -3.147 | .002 |
| *Cross-level interactions* | | |  |  |  |  |
|  | *Contact opportunities* | |  |  |  |  |
|  |  | x Extraversion | -.022 | 0.015 | -1.462 | .144 |
|  |  | x Agreeableness | .029 | 0.015 | 1.985 | .047 |
|  |  | x Openness | -.032 | 0.015 | -2.067 | .039 |
|  |  | x Neuroticism | -.016 | 0.015 | -1.075 | .282 |
|  |  | x Conscientiousness | .039 | 0.016 | 2.436 | .015 |
|  |  | x Preference Conservative Party | .019 | 0.014 | 1.344 | .179 |
|  |  | x Neighbourhood Belonging | .041 | 0.013 | 3.247 | .001 |
|  |  | x Perceived Racial Hate Crime Incidence | -.016 | 0.010 | -1.544 | .123 |
|  | *Actual Racial Hate Crimes Incidence* | |  |  |  |  |
|  |  | x Extraversion | .010 | 0.009 | 1.139 | .255 |
|  |  | x Agreeableness | -.010 | 0.009 | -1.120 | .263 |
|  |  | x Openness | -.002 | 0.009 | -0.162 | .871 |
|  |  | x Neuroticism | .009 | 0.009 | 0.997 | .319 |
|  |  | x Conscientiousness | -.010 | 0.009 | -1.064 | .287 |
|  |  | x Preference Conservative Party | -.009 | 0.008 | -1.103 | .270 |
|  |  | x Neighbourhood Belonging | .013 | 0.008 | 1.695 | .090 |
|  |  | x Perceived Racial Hate Crime Incidence | .007 | 0.007 | 1.008 | .314 |
|  | *Right-Wing Votes* | |  |  |  |  |
|  |  | x Extraversion | -.004 | 0.009 | -0.460 | .646 |
|  |  | x Agreeableness | .001 | 0.009 | 0.063 | .950 |
|  |  | x Openness | .011 | 0.009 | 1.284 | .199 |
|  |  | x Neuroticism | -.012 | 0.009 | -1.377 | .168 |
|  |  | x Conscientiousness | -.016 | 0.009 | -1.699 | .089 |
|  |  | x Preference Conservative Party | .015 | 0.009 | 1.683 | .092 |
|  |  | x Neighbourhood Belonging | -.005 | 0.008 | -0.643 | .521 |
|  |  | x Perceived Racial Hate Crime Incidence | .003 | 0.007 | 0.473 | .636 |
|  | *Brexit Leave Votes* | |  |  |  |  |
|  |  | x Extraversion | -.012 | 0.010 | -1.211 | .226 |
|  |  | x Agreeableness | -.014 | 0.010 | -1.448 | .148 |
|  |  | x Openness | .002 | 0.010 | 0.183 | .854 |
|  |  | x Neuroticism | .018 | 0.009 | 1.966 | .049 |
|  |  | x Conscientiousness | .035 | 0.010 | 3.347 | .001 |
|  |  | x Preference Conservative Party | -.002 | 0.009 | -0.172 | .864 |
|  |  | x Neighbourhood Belonging | .014 | 0.008 | 1.698 | .089 |
|  |  | x Perceived Racial Hate Crime Incidence | -.004 | 0.008 | -0.471 | .638 |
| *Two-way Group membership interactions: Asian participants (ref. White)* | | |  |  |  |  |
|  | *Group x individual-level* | |  |  |  |  |
|  |  | x Extraversion | .074 | 0.054 | 1.381 | .167 |
|  |  | x Agreeableness | -.014 | 0.050 | -0.286 | .775 |
|  |  | x Openness | -.014 | 0.055 | -0.248 | .804 |
|  |  | x Neuroticism | .015 | 0.050 | 0.295 | .768 |
|  |  | x Conscientiousness | -.003 | 0.050 | -0.066 | .947 |
|  |  | x Preference Conservative Party | .034 | 0.050 | 0.681 | .496 |
|  |  | x Neighbourhood Belonging | -.140 | 0.043 | -3.262 | .001 |
|  |  | x Perceived Racial Hate Crime Incidence | -.019 | 0.030 | -0.628 | .530 |
|  | *Group x contextual-level* | |  |  |  |  |
|  |  | Contact Opportunities | -.034 | 0.034 | -1.012 | .311 |
|  |  | Actual Racial Hate Crimes Incidence | -.047 | 0.021 | -2.209 | .027 |
|  |  | Right-Wing Votes | -.046 | 0.030 | -1.514 | .130 |
|  |  | Brexit Leave Votes | -.050 | 0.037 | -1.357 | .175 |
|  | *Group x control* | |  |  |  |  |
|  |  | Age | -.054 | 0.030 | -1.828 | .068 |
|  |  | Gender (ref. male) | .039 | 0.041 | 0.953 | .341 |
|  |  | Income | .157 | 0.035 | 4.456 | < .001 |
|  |  | Education Post Secondary (ref. Secondary) | .136 | 0.059 | 2.314 | .021 |
|  |  | Education Higher (ref. Secondary) | .036 | 0.050 | 0.722 | .470 |
|  |  | Deprivation | .052 | 0.025 | 2.056 | .040 |
|  |  | Population Density | -.005 | 0.021 | -0.243 | .808 |
|  |  | Time | .037 | 0.021 | 1.798 | .072 |
| *Two-way Group membership interactions: Black participants (ref. White)* | | |  |  |  |  |
|  | *Group x individual-level* | |  |  |  |  |
|  |  | x Extraversion | .022 | 0.193 | 0.115 | .908 |
|  |  | x Agreeableness | .167 | 0.205 | 0.815 | .415 |
|  |  | x Openness | .053 | 0.203 | 0.261 | .794 |
|  |  | x Neuroticism | .044 | 0.226 | 0.196 | .844 |
|  |  | x Conscientiousness | -.056 | 0.202 | -0.277 | .781 |
|  |  | x Preference Conservative Party | -.003 | 0.189 | -0.018 | .986 |
|  |  | x Neighbourhood Belonging | .090 | 0.162 | 0.553 | .580 |
|  |  | x Perceived Racial Hate Crime Incidence | -.252 | 0.134 | -1.882 | .060 |
|  | *Group x contextual-level* | |  |  |  |  |
|  |  | Contact Opportunities | -.017 | 0.097 | -0.175 | .861 |
|  |  | Actual Racial Hate Crimes Incidence | -.097 | 0.035 | -2.795 | .005 |
|  |  | Right-Wing Votes | -.203 | 0.055 | -3.708 | < .001 |
|  |  | Brexit Leave Votes | .079 | 0.056 | 1.401 | .161 |
|  | *Group x control* | |  |  |  |  |
|  |  | Age | .017 | 0.039 | 0.438 | .661 |
|  |  | Gender (ref. male) | -.002 | 0.060 | -0.038 | .970 |
|  |  | Income | -.085 | 0.061 | -1.393 | .164 |
|  |  | Education Post Secondary (ref. Secondary) | .000 | 0.088 | 0.002 | .998 |
|  |  | Education Higher (ref. Secondary) | -.098 | 0.065 | -1.498 | .134 |
|  |  | Deprivation | .200 | 0.044 | 4.594 | < .001 |
|  |  | Population Density | .005 | 0.028 | 0.179 | .858 |
|  |  | Time | 0.058 | 0.033 | 1.779 | .075 |
| *Three-way group membership interactions: Asian participants (ref. White)* | | |  |  |  |  |
|  | *Contact opportunities x group* | |  |  |  |  |
|  |  | x Extraversion | .004 | 0.027 | 0.149 | .881 |
|  |  | x Agreeableness | -.034 | 0.025 | -1.338 | .181 |
|  |  | x Openness | .043 | 0.027 | 1.576 | .115 |
|  |  | x Neuroticism | .035 | 0.025 | 1.371 | .170 |
|  |  | x Conscientiousness | -.017 | 0.026 | -0.667 | .505 |
|  |  | x Preference Conservative Party | -.015 | 0.025 | -0.602 | .547 |
|  |  | x Neighbourhood Belonging | -.012 | 0.022 | -0.573 | .567 |
|  |  | x Perceived Racial Hate Crime Incidence | .007 | 0.016 | 0.454 | .650 |
|  | *Actual Racial Hate Crimes Incidence x group* | |  |  |  |  |
|  |  | x Extraversion | -.002 | 0.019 | -0.124 | .901 |
|  |  | x Agreeableness | .034 | 0.018 | 1.865 | .062 |
|  |  | x Openness | -.037 | 0.020 | -1.872 | .061 |
|  |  | x Neuroticism | -.027 | 0.019 | -1.456 | .145 |
|  |  | x Conscientiousness | .022 | 0.020 | 1.101 | .271 |
|  |  | x Preference Conservative Party | .020 | 0.018 | 1.076 | .282 |
|  |  | x Neighbourhood Belonging | -.046 | 0.016 | -2.791 | .005 |
|  |  | x Perceived Racial Hate Crime Incidence | -.007 | 0.012 | -0.567 | .570 |
|  | *Right-Wing Votes x group* | |  |  |  |  |
|  |  | x Extraversion | .012 | 0.027 | 0.449 | .653 |
|  |  | x Agreeableness | -.011 | 0.026 | -0.410 | .682 |
|  |  | x Openness | -.004 | 0.029 | -0.127 | .899 |
|  |  | x Neuroticism | .077 | 0.028 | 2.725 | .006 |
|  |  | x Conscientiousness | -.005 | 0.026 | -0.183 | .855 |
|  |  | x Preference Conservative Party | -.054 | 0.030 | -1.814 | .070 |
|  |  | x Neighbourhood Belonging | .003 | 0.023 | 0.139 | .890 |
|  |  | x Perceived Racial Hate Crime Incidence | .006 | 0.016 | 0.391 | .696 |
|  | *Brexit Leave Votes x group* | |  |  |  |  |
|  |  | x Extraversion | -.053 | 0.032 | -1.629 | .103 |
|  |  | x Agreeableness | .096 | 0.032 | 3.054 | .002 |
|  |  | x Openness | -.064 | 0.033 | -1.924 | .054 |
|  |  | x Neuroticism | -.091 | 0.033 | -2.727 | .006 |
|  |  | x Conscientiousness | -.026 | 0.032 | -0.815 | .415 |
|  |  | x Preference Conservative Party | .031 | 0.033 | 0.939 | .348 |
|  |  | x Neighbourhood Belonging | -.065 | 0.028 | -2.295 | .022 |
|  |  | x Perceived Racial Hate Crime Incidence | .003 | 0.019 | 0.176 | .860 |
| *Three-way group membership interactions: Black participants (ref. White)* | | |  |  |  |  |
|  | *Contact opportunities x group* | |  |  |  |  |
|  |  | x Extraversion | .075 | 0.066 | 1.133 | .257 |
|  |  | x Agreeableness | -.111 | 0.069 | -1.608 | .108 |
|  |  | x Openness | -.014 | 0.070 | -0.201 | .841 |
|  |  | x Neuroticism | .053 | 0.076 | 0.697 | .486 |
|  |  | x Conscientiousness | -.020 | 0.069 | -0.289 | .773 |
|  |  | x Preference Conservative Party | -.012 | 0.065 | -0.189 | .850 |
|  |  | x Neighbourhood Belonging | -.075 | 0.055 | -1.356 | .175 |
|  |  | x Perceived Racial Hate Crime Incidence | .092 | 0.045 | 2.025 | .043 |
|  | *Actual Racial Hate Crimes Incidence*  *x group* | |  |  |  |  |
|  |  | x Extraversion | -.035 | 0.023 | -1.533 | .125 |
|  |  | x Agreeableness | .018 | 0.025 | 0.696 | .487 |
|  |  | x Openness | .026 | 0.025 | 1.043 | .297 |
|  |  | x Neuroticism | -.023 | 0.026 | -0.887 | .375 |
|  |  | x Conscientiousness | .000 | 0.025 | 0.019 | .985 |
|  |  | x Preference Conservative Party | .020 | 0.024 | 0.831 | .406 |
|  |  | x Neighbourhood Belonging | -.010 | 0.021 | -0.499 | .618 |
|  |  | x Perceived Racial Hate Crime Incidence | .011 | 0.019 | 0.576 | .564 |
|  | *Right-Wing Votes x group* | |  |  |  |  |
|  |  | x Extraversion | -.030 | 0.031 | -0.952 | .341 |
|  |  | x Agreeableness | .041 | 0.042 | 0.981 | .326 |
|  |  | x Openness | .087 | 0.041 | 2.136 | .033 |
|  |  | x Neuroticism | .101 | 0.037 | 2.749 | .006 |
|  |  | x Conscientiousness | .018 | 0.041 | 0.446 | .656 |
|  |  | x Preference Conservative Party | -.149 | 0.040 | -3.742 | < .001 |
|  |  | x Neighbourhood Belonging | -.027 | 0.029 | -0.948 | .343 |
|  |  | x Perceived Racial Hate Crime Incidence | -.005 | 0.021 | -0.251 | .802 |
|  | *Brexit Leave Votes x group* | |  |  |  |  |
|  |  | x Extraversion | .054 | 0.038 | 1.440 | .150 |
|  |  | x Agreeableness | .005 | 0.042 | 0.120 | .904 |
|  |  | x Openness | -.120 | 0.044 | -2.707 | .007 |
|  |  | x Neuroticism | -.077 | 0.042 | -1.837 | .066 |
|  |  | x Conscientiousness | -.037 | 0.045 | -0.835 | .404 |
|  |  | x Preference Conservative Party | .055 | 0.042 | 1.303 | .192 |
|  |  | x Neighbourhood Belonging | -.037 | 0.034 | -1.102 | .270 |
|  |  | x Perceived Racial Hate Crime Incidence | -.013 | 0.026 | -0.507 | .612 |

*Note*. n individual-level = 18806; n LSOA level = 11181; n LAD level = 320; all estimates are based on standardised regression coefficients

**Appendix S4**

**Full Results Main Analysis**

The main text did not include the results for the control variables and their group membership interactions, or the three-way interactions between individual-level predictors, contextual-level predictors and group membership. See Table S11 for the full results of the multilevel model and Figure S2-S4 for a visual representation of the significant two- and three-way interactions.

**Table S11**

*Full Results Table of Full Multilevel Model including Control Variables*

|  |  | | *β* | *se* | *t* | *p* |
| --- | --- | --- | --- | --- | --- | --- |
|  |  | *Intercept* | .074 | 0.017 | 4.372 | < .001 |
|  |  | *Time* | -.003 | 0.005 | -0.503 | .615 |
|  |  | *Group Membership (ref. Majority)* | .069 | 0.075 | 0.919 | .358 |
| *Control* | | |  |  |  |  |
|  |  | Age | -.106 | 0.007 | -15.206 | < .001 |
|  |  | Gender (ref. male) | -.030 | 0.012 | -2.475 | .013 |
|  |  | Income | .004 | 0.005 | 0.796 | .426 |
|  |  | Education Post Secondary (ref. Secondary) | .044 | 0.018 | 2.447 | .014 |
|  |  | Education Higher (ref. Secondary) | .080 | 0.013 | 6.265 | < .001 |
|  |  | Deprivation | -.022 | 0.007 | -3.188 | .001 |
|  |  | Population Density | .034 | 0.010 | 3.530 | < .001 |
| *Individual-Level* | | |  |  |  |  |
|  |  | Extraversion | .006 | 0.007 | 0.787 | .431 |
|  |  | Agreeableness | .019 | 0.007 | 2.583 | .010 |
|  |  | Openness | .050 | 0.008 | 6.622 | < .001 |
|  |  | Neuroticism | -.015 | 0.007 | -2.071 | .038 |
|  |  | Conscientiousness | .007 | 0.008 | 0.858 | .391 |
|  |  | Preference Conservative Party | -.030 | 0.007 | -4.232 | < .001 |
|  |  | Neighbourhood Belonging | .022 | 0.006 | 3.567 | < .001 |
|  |  | Perceived Racial Hate Crime Incidence | .026 | 0.006 | 4.674 | < .001 |
| *Contextual-Level* | | |  |  |  |  |
|  |  | Contact Opportunities | .290 | 0.019 | 15.158 | < .001 |
|  |  | Actual Racial Hate Crime Incidence | .041 | 0.012 | 3.458 | .001 |
|  |  | Right-Wing Votes | .003 | 0.011 | 0.300 | .765 |
|  |  | Brexit Leave Votes | -.044 | 0.014 | -3.131 | .002 |
| *Cross-level interactions* | | |  |  |  |  |
|  | *Contact opportunities* | |  |  |  |  |
|  |  | x Extraversion | -.023 | 0.015 | -1.479 | .139 |
|  |  | x Agreeableness | .029 | 0.015 | 1.976 | .048 |
|  |  | x Openness | -.032 | 0.015 | -2.088 | .037 |
|  |  | x Neuroticism | -.016 | 0.015 | -1.075 | .282 |
|  |  | x Conscientiousness | .039 | 0.016 | 2.443 | .015 |
|  |  | x Preference Conservative Party | .019 | 0.014 | 1.366 | .172 |
|  |  | x Neighbourhood Belonging | .041 | 0.013 | 3.260 | .001 |
|  |  | x Perceived Racial Hate Crime Incidence | -.016 | 0.010 | -1.580 | .114 |
|  | *Actual Racial Hate Crime Incidence* | |  |  |  |  |
|  |  | x Extraversion | .010 | 0.009 | 1.151 | .250 |
|  |  | x Agreeableness | -.010 | 0.009 | -1.104 | .270 |
|  |  | x Openness | -.001 | 0.009 | -0.148 | .883 |
|  |  | x Neuroticism | .009 | 0.009 | 1.004 | .316 |
|  |  | x Conscientiousness | -.010 | 0.009 | -1.055 | .291 |
|  |  | x Preference Conservative Party | -.009 | 0.008 | -1.107 | .268 |
|  |  | x Neighbourhood Belonging | .013 | 0.008 | 1.708 | .088 |
|  |  | x Perceived Racial Hate Crime Incidence | .007 | 0.007 | 1.005 | .315 |
|  | *Right-Wing Votes* | |  |  |  |  |
|  |  | x Extraversion | -.004 | 0.009 | -0.450 | .652 |
|  |  | x Agreeableness | .001 | 0.009 | 0.074 | .941 |
|  |  | x Openness | .011 | 0.009 | 1.293 | .196 |
|  |  | x Neuroticism | -.012 | 0.009 | -1.365 | .172 |
|  |  | x Conscientiousness | -.016 | 0.009 | -1.708 | .088 |
|  |  | x Preference Conservative Party | .015 | 0.009 | 1.680 | .093 |
|  |  | x Neighbourhood Belonging | -.005 | 0.008 | -0.639 | .523 |
|  |  | x Perceived Racial Hate Crime Incidence | .003 | 0.007 | 0.483 | .629 |
|  | *Brexit Leave Votes* | |  |  |  |  |
|  |  | x Extraversion | -.012 | 0.010 | -1.221 | .222 |
|  |  | x Agreeableness | -.014 | 0.010 | -1.441 | .150 |
|  |  | x Openness | .002 | 0.010 | 0.172 | .864 |
|  |  | x Neuroticism | .018 | 0.009 | 1.959 | .050 |
|  |  | x Conscientiousness | .035 | 0.010 | 3.358 | .001 |
|  |  | x Preference Conservative Party | -.002 | 0.009 | -0.163 | .871 |
|  |  | x Neighbourhood Belonging | .014 | 0.008 | 1.686 | .092 |
|  |  | x Perceived Racial Hate Crime Incidence | -.004 | 0.008 | -0.480 | .632 |
| *Two-way Group membership interactions* | | |  |  |  |  |
|  | *Group (ref. Majority) x individual-level* | |  |  |  |  |
|  |  | x Extraversion | .060 | 0.051 | 1.178 | .239 |
|  |  | x Agreeableness | .030 | 0.047 | 0.640 | .522 |
|  |  | x Openness | -.004 | 0.052 | -0.086 | .931 |
|  |  | x Neuroticism | .008 | 0.047 | 0.160 | .873 |
|  |  | x Conscientiousness | .007 | 0.047 | 0.158 | .875 |
|  |  | x Preference Conservative Party | .033 | 0.047 | 0.701 | .483 |
|  |  | x Neighbourhood Belonging | -.161 | 0.040 | -3.992 | < .001 |
|  |  | x Perceived Racial Hate Crime Incidence | -.033 | 0.028 | -1.162 | .245 |
|  | *Group (ref. Majority) x contextual-level* | |  |  |  |  |
|  |  | x Contact Opportunities | -.027 | 0.031 | -0.886 | .375 |
|  |  | x Actual Racial Hate Crime Incidence | -.063 | 0.019 | -3.337 | .001 |
|  |  | x Right-Wing Votes | -.068 | 0.027 | -2.471 | .013 |
|  |  | x Brexit Leave Votes | -.015 | 0.032 | -0.463 | .643 |
|  | *Group (ref. Majority) x control variables* | |  |  |  |  |
|  |  | x Age | -.031 | 0.024 | -1.291 | .197 |
|  |  | x Gender | .016 | 0.034 | 0.462 | .644 |
|  |  | x Income | .092 | 0.030 | 3.021 | .003 |
|  |  | x Education Post Secondary (ref. Secondary) | .088 | 0.050 | 1.755 | .079 |
|  |  | x Education Higher (ref. Secondary) | -.025 | 0.040 | -0.623 | .534 |
|  |  | x Deprivation | .104 | 0.022 | 4.767 | < .001 |
|  |  | x Population Density | .004 | 0.018 | 0.241 | .810 |
|  |  | x Time | .044 | 0.018 | 2.506 | .012 |
| *Three-way group membership interactions* | | |  |  |  |  |
|  | *Contact opportunities x group (ref. Majority)* | |  |  |  |  |
|  |  | x Extraversion | .028 | 0.024 | 1.163 | .245 |
|  |  | x Agreeableness | -.056 | 0.023 | -2.399 | .016 |
|  |  | x Openness | .031 | 0.025 | 1.218 | .223 |
|  |  | x Neuroticism | .046 | 0.024 | 1.966 | .049 |
|  |  | x Conscientiousness | -.032 | 0.024 | -1.344 | .179 |
|  |  | x Preference Conservative Party | -.016 | 0.023 | -0.675 | .500 |
|  |  | x Neighbourhood Belonging | .003 | 0.020 | 0.160 | .873 |
|  |  | x Perceived Racial Hate Crime Incidence | .017 | 0.015 | 1.126 | .260 |
|  | *Actual Racial Hate Crime Incidence x group (ref. Majority)* | |  |  |  |  |
|  |  | x Extraversion | -.012 | 0.015 | -0.770 | .441 |
|  |  | x Agreeableness | .023 | 0.016 | 1.445 | .148 |
|  |  | x Openness | -.012 | 0.017 | -0.721 | .471 |
|  |  | x Neuroticism | -.022 | 0.016 | -1.380 | .167 |
|  |  | x Conscientiousness | .005 | 0.016 | 0.310 | .757 |
|  |  | x Preference Conservative Party | .017 | 0.015 | 1.089 | .276 |
|  |  | x Neighbourhood Belonging | -.028 | 0.014 | -2.042 | .041 |
|  |  | x Perceived Racial Hate Crime Incidence | -.005 | 0.011 | -0.416 | .677 |
|  | *Right-Wing Votes x group (ref. Majority)* | |  |  |  |  |
|  |  | x Extraversion | .002 | 0.021 | 0.102 | .919 |
|  |  | x Agreeableness | .004 | 0.023 | 0.195 | .846 |
|  |  | x Openness | .014 | 0.023 | 0.591 | .555 |
|  |  | x Neuroticism | .094 | 0.023 | 4.113 | < .001 |
|  |  | x Conscientiousness | .008 | 0.022 | 0.341 | .733 |
|  |  | x Preference Conservative Party | -.071 | 0.024 | -2.999 | .003 |
|  |  | x Neighbourhood Belonging | -.007 | 0.018 | -0.382 | .702 |
|  |  | x Perceived Racial Hate Crime Incidence | .003 | 0.013 | 0.260 | .795 |
|  | *Brexit Leave Votes x group (ref. Majority)* | |  |  |  |  |
|  |  | x Extraversion | -.010 | 0.024 | -0.409 | .682 |
|  |  | x Agreeableness | .050 | 0.025 | 1.987 | .047 |
|  |  | x Openness | -.072 | 0.027 | -2.689 | .007 |
|  |  | x Neuroticism | -.095 | 0.026 | -3.630 | < .001 |
|  |  | x Conscientiousness | -.035 | 0.026 | -1.335 | .182 |
|  |  | x Preference Conservative Party | .026 | 0.026 | 1.017 | .309 |
|  |  | x Neighbourhood Belonging | -.069 | 0.022 | -3.151 | .002 |
|  |  | x Perceived Racial Hate Crime Incidence | -.002 | 0.016 | -0.113 | .910 |
|  |  |  |  |  |  |  |
| Variance Components | | | Variance | | R^2^ |  |
|  |  | Random Intercept LAD | .016  .031  .234  .545 | |  |  |
|  |  | Random Intercept LSOA |  |  |  |  |
|  |  | Random Intercept Participant |  |  |  |  |
|  |  | Residual |  |  |  |  |
|  |  | Random Effects (combined) |  |  | .447 |  |
|  |  | Fixed Effects |  |  | .162 |  |

*Note*. N individual-level = 18806; n LSOA level = 11181; n LAD level = 320; all estimates are based on standardised regression coefficients

**Figure S2**

*Two-way interactions between group membership and control variables*


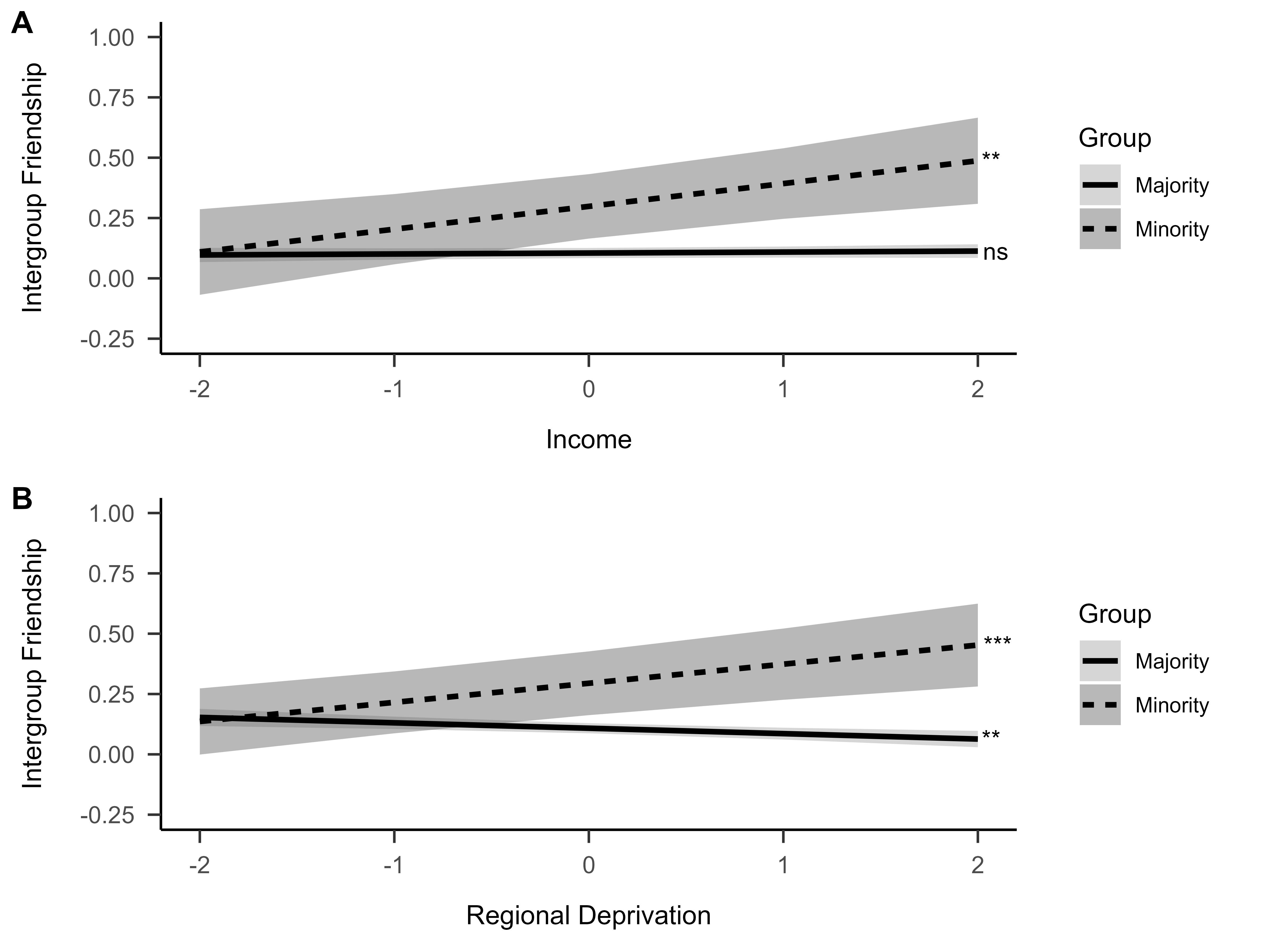


*Note*. The x-axis shows standardised predictor values, and the y-axis shows model-predicted intergroup friendship scores. Significance indicates whether slopes are significantly different from 0 at **p* < .05, ***p* < .01, ****p* < .001

**Three-way interactions full model**

We reported on the cross-level and two-way interactions with group membership in the main text. In addition to the effects reported, there were some three-way interactions, indicating that individual- and neighbourhood-level variables interact in unique ways for ethnic majority and minority group members. Firstly, the effect of local anti-immigrant norms differed as a function of individual-level variables and group membership. Neighbourhood belonging (Figure S3, panel A), neuroticism (Figure S3, panel B), and openness (Figure S3, panel C) weakened the effect of Brexit leave votes for majority group members. Simple slopes indicated that the effect of Brexit leave votes was only significant at low to average levels and not at high levels of neighbourhood belonging (low *β* = -.071, 95% CI [-.112, -.030]; average *β* = -.043, 95% CI [-.070, -.015]; high *β* = -.014, 95% CI [-.058, .031]), neuroticism (low *β* = -.080, 95% CI [-.127, -.034]; average *β* = -.043, 95% CI [-.070, -.016]; high *β* = -.006, 95% CI [-.051, .039]), and openness (low *β* = -.047, 95% CI [-.098, .004]; average *β* = -.043, 95% CI [-.071, -.016]; high *β* = -.040, 95% CI [-.085, .006]) for majority group participants. For minority group members, the pattern was reversed. Average to high levels of neighbourhood belonging (low *β* = -.040, 95% CI [-.054, .135]; average *β* = -.069, 95% CI [-.131, -.007]; high *β* = -.178, 95% CI [-.284, -.072]), neuroticism (low *β* = .080, 95% CI [-.022, .196]; average *β* = -.066, 95% CI [-.127, -.004]; high *β* = -.218, 95% CI [-.337, -.100]), and openness (low *β* = .083, 95% CI [-.038, .203]; average *β* = -.058, 95% CI [-.120, .004]; high *β* = -.198, 95% CI [-.308, -.088]) was related to a stronger effect of Brexit leave votes.

Neuroticism (Figure S4, panel A) and political orientation (Figure S4, panel B) moderated the effect of right-wing party votes, but only among minority group members. For majority group members, the simple slopes were uniformly non-significant both for neuroticism (low: β = .029, 95% CI [-.012, .069]; average: β = .005, 95% CI [-.017, .027]; high: β = -.018, 95% CI [-.058, .021]) and political orientation (low: β = -.025, 95% CI [-.065, .016]; average: β =.004, 95% CI [-.018, .027]; high: β = .033, 95% CI [-.007, .073]). In contrast, among minority group members, neuroticism significantly moderated the effect: right-wing votes predicted less intergroup friendships at low (β = -.227, 95% CI [-.321, -.132]) and average (β = -.063, 95% CI [-.116, -.010]) levels, but not at high levels (β = .101, 95% CI [-.000, .202]). Political orientation also exerted a moderating effect: somewhat unexpectedly, stronger Conservative preference amplified the relation. Here, the effect right-wing votes was significant at average (β = -.059, 95% CI [-.111, -.007]) and high (β = -.172, 95% CI [-.288, -.056]) levels of Conservative preference, but not at low levels (β = .053, 95% CI [-.029, .135]).

**Figure S3**

*Three-way interactions between group, local anti-immigrant norms in the form of Brexit leave votes, and individual-level predictors*


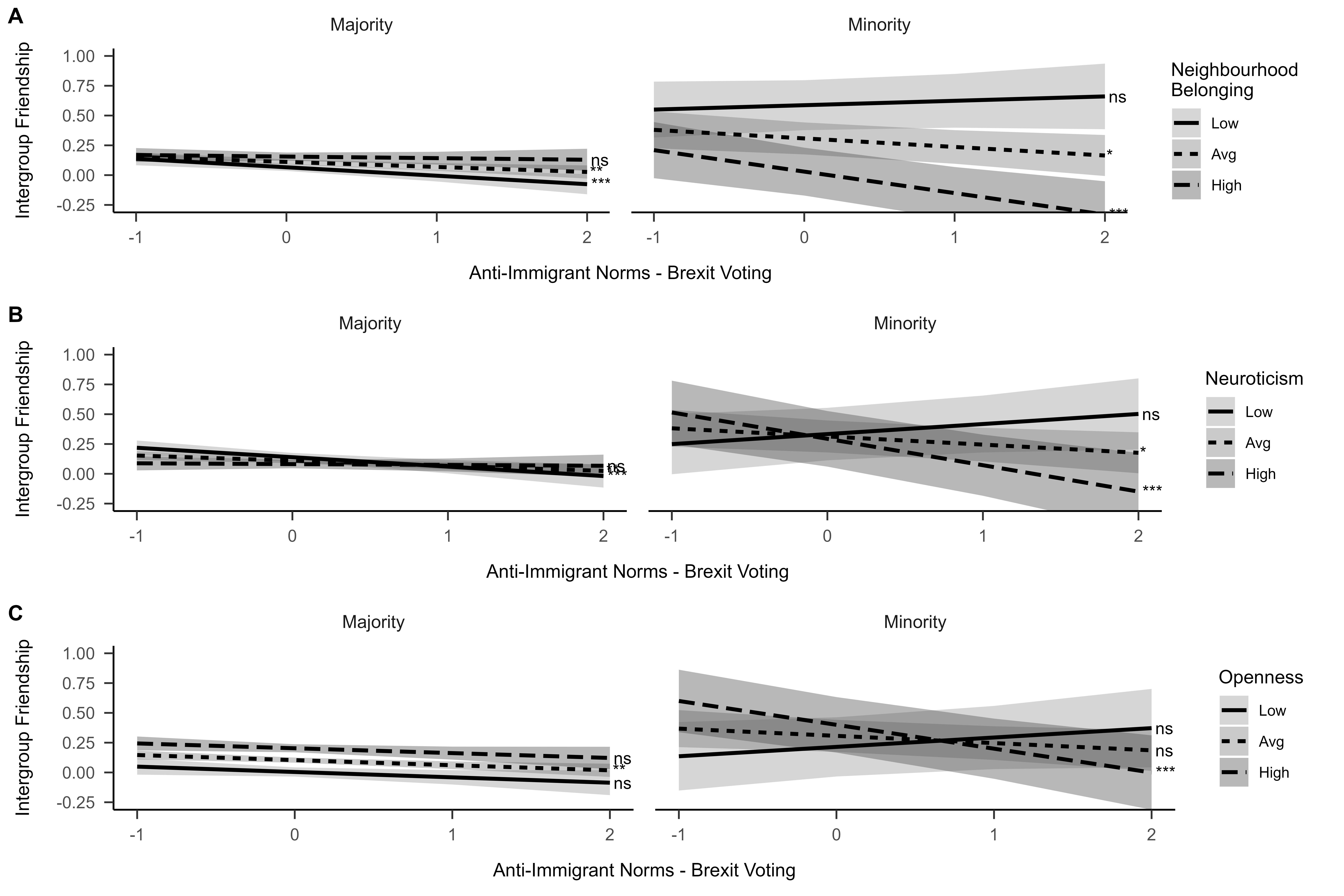


*Note*. The x-axis shows standardised predictor values, and the y-axis shows model-predicted intergroup friendship scores. Significance indicates whether slopes are significantly different from 0 at **p* < .05, ***p* < .01, ****p* < .001

**Figure S4**

*Three-way interactions between group, local anti-immigrant norms in the form of right-wing votes, and individual-level predictors*


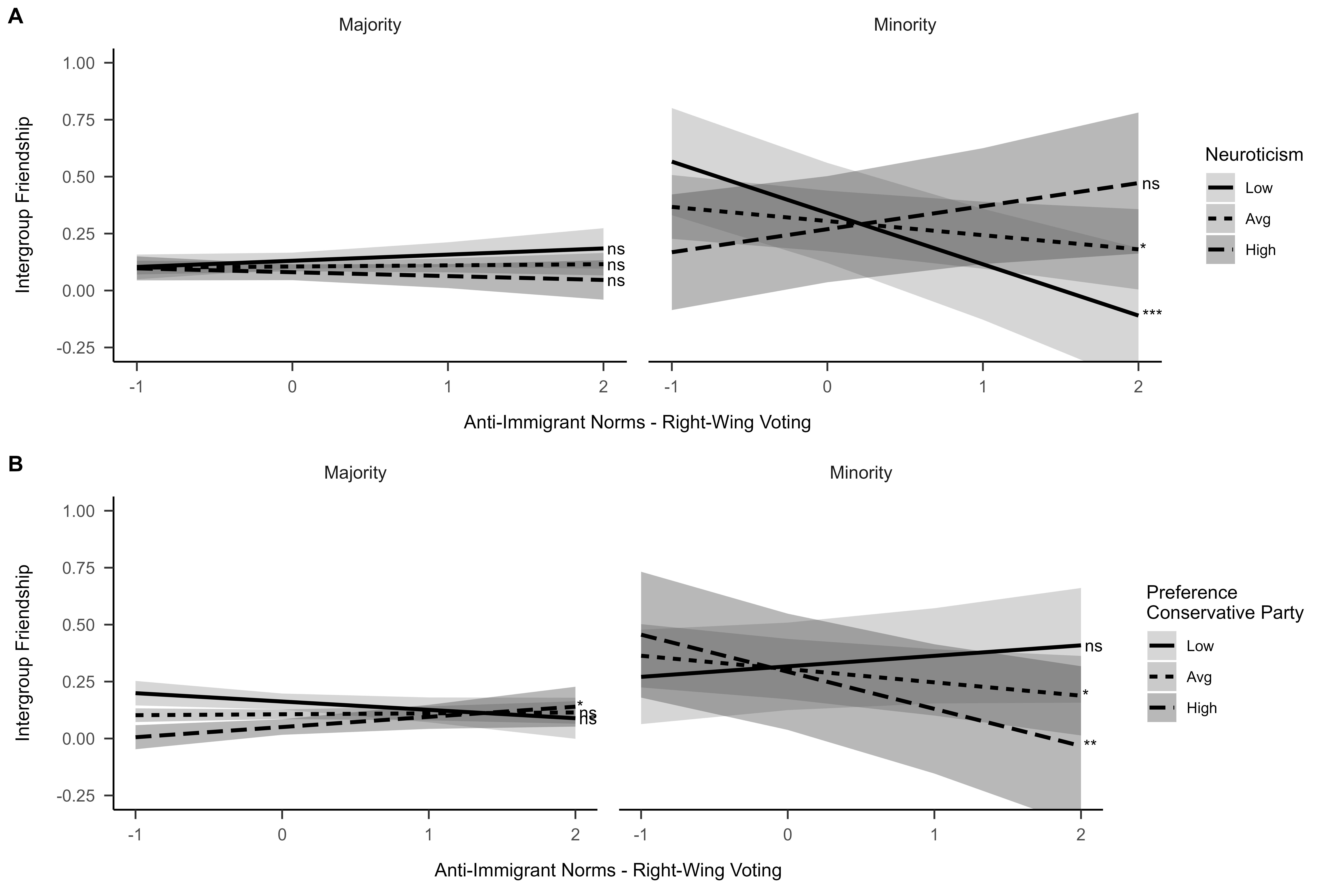


*Note*. The x-axis shows standardised predictor values, and the y-axis shows model-predicted intergroup friendship scores. Significance indicates whether slopes are significantly different from 0 at **p* < .05, ***p* < .01, ****p* < .001

**Appendix S5**

**Correlations**

We looked at the correlations between variables at each time point (see Table S12-S14). In addition, we examined the autocorrelations of time-varying variables (see Table S15).

**Table S12**

*Full Pearson correlation table T1*

|  |  | *M* | | SD | | 1 | 2 | 3 | 4 | 5 | 6 | 7 | 8 | 9 | 10 | 11 | 12 | 13 | 14 | 15 | 16 | 17 |
| --- | --- | --- | --- | --- | --- | --- | --- | --- | --- | --- | --- | --- | --- | --- | --- | --- | --- | --- | --- | --- | --- | --- |
|  | 1 Intergroup Friendship T1 | 1.77 | | 0.89 | |  |  |  |  |  |  |  |  |  |  |  |  |  |  |  |  |  |
| **Time-invariant** | |  |  | |  | |  |  |  |  |  |  |  |  |  |  |  |  |  |  |  |  |
|  | 2 Contact Opportunities | 0.16 | | 0.24 | | **.37** |  |  |  |  |  |  |  |  |  |  |  |  |  |  |  |  |
|  | 3 Brexit Leave Vote | 0.53 | | 0.10 | | **-.18** | **-.35** |  |  |  |  |  |  |  |  |  |  |  |  |  |  |  |
|  | 4 Openness | 4.71 | | 1.25 | | **.08** | **.02** | **-.08** |  |  |  |  |  |  |  |  |  |  |  |  |  |  |
|  | 5 Agreeableness | 5.60 | | 1.02 | | .01 | **.02** | **.02** | **.19** |  |  |  |  |  |  |  |  |  |  |  |  |  |
|  | 6 Extraversion | 4.63 | | 1.30 | | **.03** | **-.05** | .01 | **.24** | **.15** |  |  |  |  |  |  |  |  |  |  |  |  |
|  | 7 Neuroticism | 3.59 | | 1.41 | | -.01 | **-.03** | **.02** | **-.11** | **-.07** | **-.18** |  |  |  |  |  |  |  |  |  |  |  |
|  | 8 Conscientiousness | 5.47 | | 1.07 | | -.01 | **-.03** | **.02** | **.18** | **.30** | **.18** | **-.17** |  |  |  |  |  |  |  |  |  |  |
|  | 9 Political Orientation | -0.43 | | 3.70 | | **-.09** | **-.15** | **.06** | **-.03** | **-.02** | .01 | **-.06** | **.07** |  |  |  |  |  |  |  |  |  |
| **Time-varying** | |  |  | |  | |  |  |  |  |  |  |  |  |  |  |  |  |  |  |  |  |
|  | 10 Neighbourhood Belonging T1 | 3.52 | | 0.71 | | **-.05** | **-.08** | .01 | **.05** | **.18** | **.16** | **-.14** | **.16** | **.06** |  |  |  |  |  |  |  |  |
|  | 11 Perceived Racial Hate Crimes T1 | 1.27 | | 0.51 | | **.11** | **.22** | **-.08** | .00 | **-.02** | **-.01** | **.05** | **-.06** | **-.11** | **-.11** |  |  |  |  |  |  |  |
|  | 12 Right-Wing Votes T1 | 0.05 | | 0.02 | | **-.11** | **-.21** | **.69** | **-.05** | **.02** | **.01** | .01 | .01 | **.02** | -.01 | **-.02** |  |  |  |  |  |  |
|  | 13 Actual Racial Hate Crimes T1 | 1950 | | 2558 | | **.25** | **.57** | **-.50** | **.03** | -.01 | **-.03** | -.01 | **-.04** | **-.09** | **-.04** | **.17** | **-.30** |  |  |  |  |  |
| **Control Variables** | |  |  | |  | |  |  |  |  |  |  |  |  |  |  |  |  |  |  |  |  |
|  | 14 Age | 43.42 | | 16.60 | | **-.14** | **-.11** | .01 | **-.03** | **.06** | **-.07** | **-.14** | **.13** | **.15** | **.26** | **-.13** | **-.02** | **-.05** |  |  |  |  |
|  | 15 Income | 1500 | | 1531 | | .00 | **-.02** | **-.07** | **.03** | **-.03** | **-.01** | **-.12** | **.09** | **.07** | **.07** | **-.06** | **-.06** | **.05** | **.19** |  |  |  |
|  | 16 Population Density | 39.74 | | 41.37 | | **.22** | **.46** | **-.42** | **.03** | -.01 | **-.01** | .00 | **-.05** | **-.16** | **-.11** | **.23** | **-.26** | **.55** | **-.15** | **-.02** |  |  |
|  | 17 Deprivation | 3.15 | | 1.40 | | **-.11** | **-.28** | -.01 | **.03** | -.01 | **.02** | **-.03** | **.06** | **.20** | **.16** | **-.26** | **-.08** | **-.19** | **.18** | **.14** | **-.35** |  |

*Note*. n = 18488; boldface correlations indicate statistical significance at *p* ≤ .05

**Table S13**

*Full Pearson correlation table T2*

|  |  | *M* | | *SD* | | 1 | 2 | 3 | 4 | 5 | 6 | 7 | 8 | 9 | 10 | 11 | 12 | 13 | 14 | 15 | 16 | 17 |
| --- | --- | --- | --- | --- | --- | --- | --- | --- | --- | --- | --- | --- | --- | --- | --- | --- | --- | --- | --- | --- | --- | --- |
|  | 1 Intergroup Friendship T2 | 1.79 | | 0.90 | |  |  |  |  |  |  |  |  |  |  |  |  |  |  |  |  |  |
| **Time-invariant** | |  |  | |  | |  |  |  |  |  |  |  |  |  |  |  |  |  |  |  |  |
|  | 2 Contact Opportunities |  | |  | | **.39** |  |  |  |  |  |  |  |  |  |  |  |  |  |  |  |  |
|  | 3 Brexit Leave Vote |  | |  | | **-.17** | **-.33** |  |  |  |  |  |  |  |  |  |  |  |  |  |  |  |
|  | 4 Openness |  | |  | | **.09** | **.03** | **-.08** |  |  |  |  |  |  |  |  |  |  |  |  |  |  |
|  | 5 Agreeableness |  | |  | | **.02** | **.02** | .02 | **.19** |  |  |  |  |  |  |  |  |  |  |  |  |  |
|  | 6 Extraversion |  | |  | | .01 | **-.04** | .01 | **.24** | **.15** |  |  |  |  |  |  |  |  |  |  |  |  |
|  | 7 Neuroticism |  | |  | | -.01 | **-.03** | .01 | **-.12** | **-.06** | **-.18** |  |  |  |  |  |  |  |  |  |  |  |
|  | 8 Conscientiousness |  | |  | | **-.02** | **-.04** | **.02** | **.18** | **.30** | **.19** | **-.17** |  |  |  |  |  |  |  |  |  |  |
|  | 9 Political Orientation |  | |  | | **-.10** | **-.14** | **.06** | **-.04** | -.02 | **.00** | **-.06** | **.07** |  |  |  |  |  |  |  |  |  |
| **Time-varying** | |  |  | |  | |  |  |  |  |  |  |  |  |  |  |  |  |  |  |  |  |
|  | 10 Neighbourhood Belonging T2 | 3.61 | | 0.72 | | **-.05** | **-.08** | .01 | **.04** | **.13** | **.14** | **-.10** | **.14** | **.05** |  |  |  |  |  |  |  |  |
|  | 11 Perceived Racial Hate Crimes T2 | 1.23 | | 0.47 | | **.12** | **.20** | **-.09** | -.01 | **-.02** | **-.02** | **.04** | **-.06** | **-.09** | **-.11** |  |  |  |  |  |  |  |
|  | 12 Right-Wing Votes T2 | 0.05 | | 0.02 | | **-.11** | **-.20** | **.69** | **-.05** | **.02** | .02 | .01 | .01 | **.02** | -.01 | **-.02** |  |  |  |  |  |  |
|  | 13 Actual Racial Hate Crimes T2 | 2364 | | 3630 | | **.25** | **.56** | **48** | **.03** | -.01 | **-.02** | -.01 | **-.04** | **-.09** | **-.05** | **.16** | **-.30** |  |  |  |  |  |
| **Control Variables** | |  |  | |  | |  |  |  |  |  |  |  |  |  |  |  |  |  |  |  |  |
|  | 14 Age |  | |  | | **-.14** | **-,14** | **.02** | **-.02** | **.04** | **-.06** | **-.14** | **.11** | **.15** | **.22** | **-.12** | **.00** | **-.06** |  |  |  |  |
|  | 15 Income |  | |  | | .01 | .00 | **-.04** | .01 | **-.02** | **-.01** | **-.05** | **.03** | **.03** | .01 | **-.02** | **-.04** | **.04** | **.05** |  |  |  |
|  | 16 Population Density |  | |  | | **.22** | **.45** | **-.42** | **.03** | .00 | .00 | .01 | **-.06** | **-.17** | **-.11** | **.22** | **-.26** | **.56** | **-.16** | -.01 |  |  |
|  | 17 Deprivation |  | |  | | **-.13** | **-.29** | .01 | **.02** | -.01 | .01 | **-.03** | **.07** | **.19** | **.17** | **-.24** | **-.07** | **-.20** | **.18** | **.07** | **-.36** |  |

*Note*. n = 13410; boldface correlations indicate statistical significance at *p* ≤ .05

**Table S14**

*Full Pearson correlation table T3*

|  |  | *M* | *SD* | | 1 | | 2 | | 3 | | 4 | | 5 | | 6 | | 7 | | 8 | | 9 | | 10 | | 11 | | 12 | | 13 | | 14 | | 15 | | 16 | | 17 | |  |
| --- | --- | --- | --- | --- | --- | --- | --- | --- | --- | --- | --- | --- | --- | --- | --- | --- | --- | --- | --- | --- | --- | --- | --- | --- | --- | --- | --- | --- | --- | --- | --- | --- | --- | --- | --- | --- | --- | --- | --- |
|  | 1 Intergroup Friendship T3 | 1.73 | 0.84 | |  | |  | |  | |  | |  | |  | |  | |  | |  | |  | |  | |  | |  | |  | |  | |  | |  | |  |
| **Time-invariant** | |  | |  | |  | |  | |  | |  | |  | |  | |  | |  | |  | |  | |  | |  | |  | |  | |  | |  | |  | |
|  | 2 Contact Opportunities |  |  | | **.40** | |  | |  | |  | |  | |  | |  | |  | |  | |  | |  | |  | |  | |  | |  | |  | |  | |  |
|  | 3 Brexit Leave Vote |  |  | | **-.17** | | **-.32** | |  | |  | |  | |  | |  | |  | |  | |  | |  | |  | |  | |  | |  | |  | |  | |  |
|  | 4 Openness |  |  | | **.08** | | **.03** | | **-.08** | |  | |  | |  | |  | |  | |  | |  | |  | |  | |  | |  | |  | |  | |  | |  |
|  | 5 Agreeableness |  |  | | .01 | | **.02** | | **.02** | | **.19** | |  | |  | |  | |  | |  | |  | |  | |  | |  | |  | |  | |  | |  | |  |
|  | 6 Extraversion |  |  | | **.02** | | **-.04** | | .01 | | **.24** | | **.16** | |  | |  | |  | |  | |  | |  | |  | |  | |  | |  | |  | |  | |  |
|  | 7 Neuroticism |  |  | | -.01 | | **-.03** | | .02 | | **-.11** | | **-.06** | | **-.18** | |  | |  | |  | |  | |  | |  | |  | |  | |  | |  | |  | |  |
|  | 8 Conscientiousness |  |  | | -.01 | | **-.03** | | **.03** | | **.17** | | **.30** | | **.19** | | **-.16** | |  | |  | |  | |  | |  | |  | |  | |  | |  | |  | |  |
|  | 9 Political Orientation |  |  | | **-.10** | | **-.13** | | **.05** | | **-.05** | | **-.02** | | .00 | | **-.06** | | **.05** | |  | |  | |  | |  | |  | |  | |  | |  | |  | |  |
| **Time-varying** | |  | |  | |  | |  | |  | |  | |  | |  | |  | |  | |  | |  | |  | |  | |  | |  | |  | |  | |  | |
|  | 10 Neighbourhood Belonging T3 | 3.54 | 0.75 | | **-.03** | | **-.06** | | -.01 | | **.06** | | **.14** | | **.15** | | **-.11** | | **.13** | | **.04** | |  | |  | |  | |  | |  | |  | |  | |  | |  |
|  | 11 Perceived Racial Hate Crimes T3 | 1.27 | 0.50 | | **.13** | | **.19** | | **-.05** | | .00 | | -.02 | | **-.02** | | **.04** | | **-.03** | | **-.09** | | **-.11** | |  | |  | |  | |  | |  | |  | |  | |  |
|  | 12 Right-Wing Votes T3 | 0.05 | 0.02 | | **-.11** | | **-.19** | | **.69** | | **-.06** | | .02 | | .01 | | .01 | | .01 | | .02 | | **-.03** | | .00 | |  | |  | |  | |  | |  | |  | |  |
|  | 13 Actual Racial Hate Crimes T3 | 3201 | 4819 | | **.26** | | **.55** | | **-.46** | | **.03** | | -.01 | | **-.02** | | -.01 | | **-.03** | | **-.07** | | **-.04** | | **.16** | | **-.29** | |  | |  | |  | |  | |  | |  |
| **Control Variables** | |  | |  | |  | |  | |  | |  | |  | |  | |  | |  | |  | |  | |  | |  | |  | |  | |  | |  | |  | |
|  | 14 Age |  |  | | **-.15** | | **-.12** | | -.00 | | .00 | | **.05** | | **-.05** | | **-.15** | | **.11** | | **.14** | | **.23** | | **-.13** | | -.01 | | **-.05** | |  | |  | |  | |  | |  |
|  | 15 Income |  |  | | .01 | | .01 | | **-.09** | | **.03** | | **-.04** | | .00 | | **-.09** | | **.04** | | **.06** | | .01 | | **-.03** | | **-.08** | | **.07** | | **.04** | |  | |  | |  | |  |
|  | 16 Population Density |  |  | | **.24** | | **.44** | | **-.39** | | **.05** | | -.01 | | -.01 | | .00 | | **-.06** | | **-.15** | | **-.09** | | **.22** | | **-.25** | | **.54** | | **-.14** | | -.01 | |  | |  | |  |
|  | 17 Deprivation |  |  | | **-.13** | | **-.26** | | **-.03** | | .01 | | -.01 | | .01 | | **-.02** | | **.06** | | **.18** | | **.14** | | **-.22** | | **-.08** | | **-.18** | | **.17** | | **.11** | | **-.34** | |  | |  |

*Note*. n = 10532; boldface correlations indicate statistical significance at *p* ≤ .05

**Table S15**

*Pearson correlation of time-varying variables between timepoints*

|  | T1 → T2 | T2 → T3 |
| --- | --- | --- |
| Intergroup Friendship | **.45** | **.49** |
| Neighbourhood Belonging | **.62** | **.60** |
| Perceived Racial Hate Crimes | **.26** | **.25** |
| Right-Wing Votes | **.94** | **.95** |
| Actual Racial Hate Crimes | **.95** | **.96** |

*Note*. n = 9497-18488 using pairwise deletion; boldface correlations indicate statistical significance at *p* ≤ .05

**References OSM**

Chen, F. F. (2007). Sensitivity of goodness of fit indexes to lack of measurement invariance. *Structural Equation Modeling: A Multidisciplinary Journal*, *14*(3), 464–504. https://doi.org/10.1080/10705510701301834

Meade, A. W., Johnson, E. C., & Braddy, P. W. (2008). Power and sensitivity of alternative fit indices in tests of measurement invariance. *Journal of Applied Psychology*, *93*(3), 568–592. https://doi.org/10.1037/0021-9010.93.3.568
